# Theoretical study on the influence of OH group position on the free radical scavenging ability of tryptamine derivatives†

**DOI:** 10.1039/d5ra01364j

**Published:** 2025-04-11

**Authors:** Dinh Quy Huong, Duong Tuan Quang, Le Quoc Thang, Nhan Thi Thanh Dang, Nguyen Le My Linh, Nguyen Minh Thong, Nguyen Minh Tam, Quan V. Vo, Pham Cam Nam

**Affiliations:** a Department of Chemistry, University of Education, Hue University Hue Vietnam dqhuong@hueuni.edu.vn; b The University of Danang-University of Science and Education Danang Vietnam; c Faculty of Basic Sciences, University of Phan Thiet 225 Nguyen Thong Phan Thiet City Binh Thuan Vietnam; d Faculty of Chemical Technology-Environment, The University of Danang-University of Technology and Education Danang Vietnam; e Department of Chemical Engineering, The University of Danang – University of Science and Technology Danang Vietnam

## Abstract

A density functional theory (DFT) study investigated the influence of hydroxyl (OH) groups on the free radical scavenging capacity of tryptamine derivatives (OH-TAs). Stable forms of OH-TAs were evaluated in both gaseous and aqueous environments. While neutral forms predominate in the gas phase, cationic forms (protonated at the NH_2_ group) are favored in aqueous solution. Frontier molecular orbital (FMO) and molecular electrostatic potential (MEP) analyses identified active sites. Electron-donating regions are primarily located on the benzene and pyrrole rings, whereas electron-accepting areas are concentrated on the OH and NH groups. In water, the lowest unoccupied molecular orbitals (LUMOs) of OH-TAs shift and localize on the NH_3_^+^ group. Calculated global reactivity descriptors indicate that 9-OH-TA exhibits the highest free radical scavenging efficiency. Molecular electrostatic potential (MEP) analysis further reveals that the hydrogen atoms in the –OH groups of the OH-TAs are the most favorable sites for nucleophilic attacks. Thermodynamic parameters, including bond dissociation energy (BDE), ionization potential (IP), and proton affinity (PA), were calculated to assess antioxidant properties. Reaction mechanism analyses reveal that hydrogen atom transfer (HAT) is the preferred pathway in the gas phase, while the single electron transfer (SET) mechanism dominates in aqueous solutions at physiological pH (7.4). Among the studied derivatives, 9-OH-TA demonstrates the highest efficiency in scavenging HOO˙ radicals, with total rate constants of 1.9 × 10^7^ M^−1^ s^−1^ in the gas phase and 5.2 × 10^7^ M^−1^ s^−1^ in water.

## Abstract 2

The position of the hydroxyl group significantly influences the free radical scavenging activity of tryptamine derivatives.

## Graphical abstract

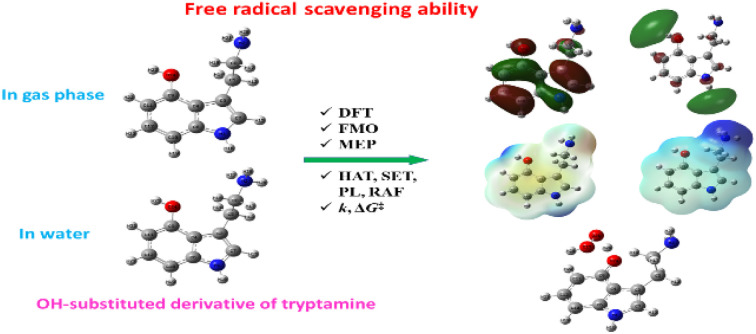

## 1. Introduction

Free radicals are molecules or molecular fragments containing one or more unpaired electrons in their valence shell, making them highly reactive.^1^ These species can either accept or donate electrons,^2^ acting as oxidizing or reducing agents. Antioxidants, even at low concentrations, effectively inhibit oxidative damage to cells by scavenging these free radicals.^3^ The pursuit of potent antioxidants has become a focal point of scientific research worldwide, as demonstrated by the significant rise in related publications in recent years.

**Fig. 1:**
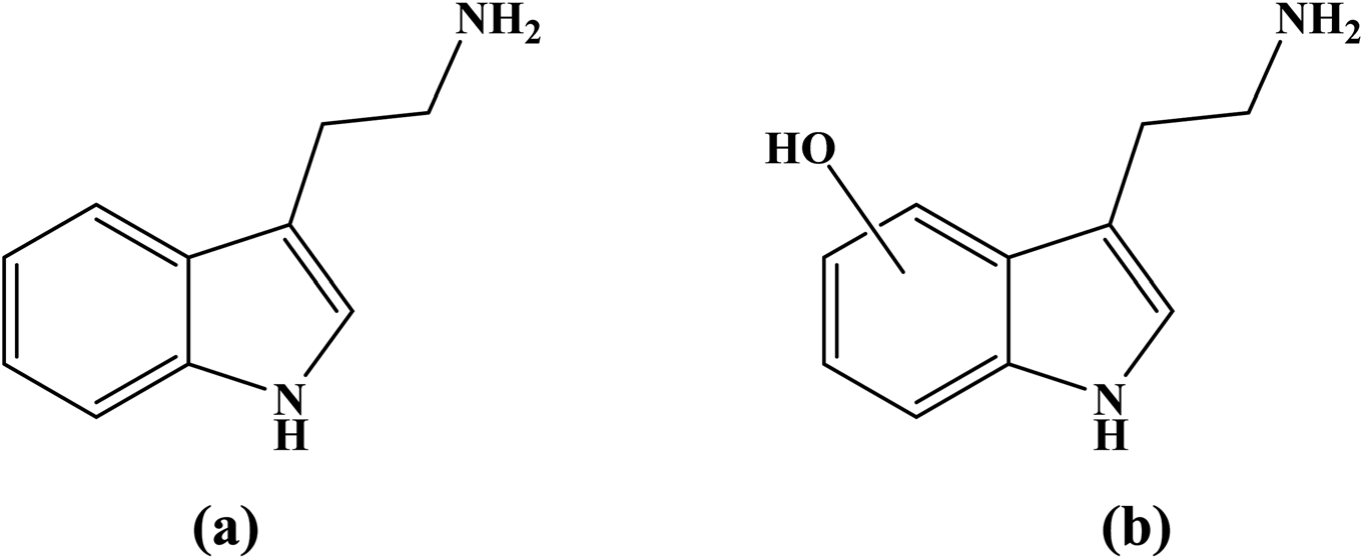
Structure of (a) tryptamine and (b) its OH-containing derivatives.

Tryptamine (3-[2-aminoethyl]indole) (Fig. 1) is a neurotransmitter that plays a significant role in regulating human behavioral and physiological states.^4^ Additionally, in plants, tryptamine serves as a precursor in metabolic pathways leading to the synthesis of indole alkaloids, such as vincristine and vinblastine, which are extensively studied for their applications in medicine and pharmacology.^5^ Notably, tryptamine is also recognized for its potent free radical scavenging properties. A computational kinetics study has evaluated its antioxidant activity against hydroxyl (HO˙) and hydroperoxyl (HOO˙) radicals in water at 298 K. The findings indicate that tryptamine is a more effective scavenger of hydroxyl radicals than hydroperoxyl radicals, exhibiting a higher rate constant for hydroxyl radical scavenging.^6^

Several derivatives of tryptamine demonstrate remarkable antioxidant activity, with melatonin (*N*-acetyl-5-methoxytryptamine) standing out as a particular example. Melatonin has garnered significant interest due to its diverse biological functions, including immune enhancement, anti-inflammatory properties, a role in maintaining mitochondrial homeostasis, and the inhibition of cancer progression. Furthermore, melatonin exhibits many characteristics of an ideal antioxidant, interacting with free radicals primarily through electron transfer and hydrogen atom transfer mechanisms, thus effectively mitigating oxidative stress.^7^

Another significant derivative is serotonin (11-OH-tryptamine), a hydroxyl-containing compound regarded as the oxidized form of biogenic amine neurotransmitters. Studies on the electronic properties and chemical reactivity of serotonin in both gas and solution phases have revealed its notable antioxidant capacity.^8^ Furthermore, serotonin exhibits substantial radical scavenging activity against peroxide anion (O_2_^−^) and hydrogen peroxide (H_2_O_2_).^9^ The free radical scavenging activity of serotonin is primarily concentrated at the hydroxyl (OH) group position. The presence of the OH group, characterized by its low bond dissociation energy, significantly enhances the free radical scavenging potential of derivatives.^10^

Recent studies have highlighted the critical role of the positional arrangement of hydroxyl (OH) groups in determining the antioxidant capacity of organic compounds. For instance, research by A. Barzegar explored the molecular characteristics of myricetin, including its antioxidant activities, OH group number and position–activity relationships, and radical scavenging mechanisms.^11^ The study predicted the sequential activity of OH groups for hydrogen atom abstraction as 4′-OH > 3′-OH ≈ 5′-OH > 3-OH > 7-OH > 5-OH, correlating with activity primarily in the B-ring, followed by the pyrone C-ring and then the A-ring. These findings provide valuable insights into the radical scavenging mechanisms of flavonoid antioxidants and aid in designing novel antioxidant compounds.^12^

Similarly, P. Trouillas and colleagues employed quantum-chemical methods to investigate the reactivity of two flavonoids, quercetin and taxifolin, focusing on the 3-OH group and its surroundings.^12^ Their analysis of theoretical bond dissociation energy (BDE) values for all OH sites revealed the significance of the B-ring and the 3-OH group specifically when a 2,3-double bond is present, as in quercetin. The study showed that the 3-OH quercetin radical exhibits a high spin density on the C-2 atom, explaining the C-ring opening process observed during redox reactions and metabolization.

In a study conducted by H. M. Ali and colleagues, the 2,2-diphenyl-1-picrylhydrazyl assay was employed to assess the free radical scavenging activity of polyphenols.^13^ The findings revealed that polyphenols containing a secondary hydroxyl group at the *ortho* or *para* positions (*e.g.*, catechol and hydroquinone) exhibited remarkable free radical scavenging activity, exceeding 97%. In contrast, *meta* isomers such as resorcinol displayed significantly lower activity. This disparity was attributed to the strong electron-donating properties of hydroxyl groups located at the *ortho* and *para* positions, which stabilize the resulting phenoxyl radicals through inductive and resonance effects. This stabilization reduces the O–H bond dissociation energy, thereby enhancing the radical scavenging efficiency. The study provided important insights into the correlation between molecular structure and antioxidant activity.

Most recently, our investigation into hydroxyl-containing derivatives of hydroxycoumarin revealed that variations in the positional arrangement of OH groups significantly affected the antioxidant capacity of these derivatives.^14^ Among them, 6-OH-hydroxycoumarin emerged as the most potent antioxidant, exhibiting the highest rate constant in its reaction with the HOO˙ free radicals. This finding further underscores the importance of OH group positioning in modulating antioxidant activity.

This study aims to enhance the antioxidant activity of tryptamine by investigating its hydroxyl (OH)-substituted derivatives (denoted as OH-TAs) (Fig. 1). Specifically, it will determine the optimal OH group position for maximizing free radical scavenging efficiency. Density functional theory (DFT) calculations, a well-established method for characterizing antioxidant properties, will be employed.

This study will thoroughly examine key structural features influencing radical scavenging activity, including solvent effects. Frontier molecular orbitals and molecular electrostatic potential (MEP) analysis will identify reactive regions. Four primary radical scavenging mechanisms-hydrogen atom transfer (HAT), proton loss (PL), single electron transfer (SET), and radical adduct formation (RAF) will be investigated, as these mechanisms have demonstrated high efficacy in some tryptamine derivatives with significant antioxidant potential.^15^

## 2. Methods

In this study, all DFT calculations were carried out using the Gaussian 16 software.^16^ Molecular geometries were optimized, and thermodynamic calculations were conducted at the M06-2X/6-311++G(d,p) level of theory.^17^ Global reactivity descriptors, including the highest occupied molecular orbital energy (*E*_HOMO_), the lowest unoccupied molecular orbital energy (*E*_LUMO_), LUMO–HOMO energy gap (Δ*E*_L–H_), hardness (*η*), and electronegativity (*χ*), were evaluated to compare the reactivity of OH-TAs in the gas phase and water. These parameters were calculated using the appropriate equations.^18^

1 Δ*E*_L–H_ = *E*_LUMO_ – *E*_HOMO_

2
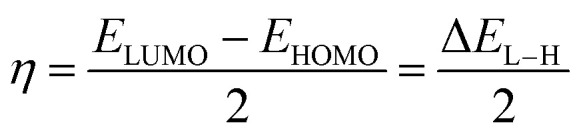
3
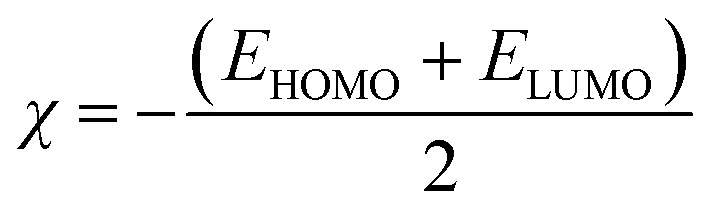


The SMD (solvation model based on density) is a universal implicit solvation model widely employed in quantum chemistry to account for solvent effects on the electronic structure of molecules. By treating the solvent as a continuous polarizable medium rather than explicitly modeling individual solvent molecules, SMD provides an efficient and accurate representation of solvation. In this study, the SMD polarizable continuum solvation model was utilized to optimize molecular geometries and to describe reactions occurring in aqueous solutions.^19^

The hydroperoxyl radical (HOO˙) is a type of reactive oxygen species (ROS) characterized by its strong oxidizing ability, actively participating in various chemical reactions, including the formation of oxidation products and the decomposition of organic compounds.^20^ In this study, HOO˙ was selected as a representative radical to assess the free radical scavenging activity of hydroxyl derivatives of tryptamine.

Free radical scavenging occurs through several mechanisms, with hydrogen atom transfer (HAT), proton loss (PL), single electron transfer (SET), and radical adduct formation (RAF) being among the most prominent pathways in organic molecules exhibiting antioxidant properties.^21^ These mechanisms are evaluated based on key thermodynamic parameters, including bond dissociation enthalpy (BDE), ionization potential (IP), and proton affinity (PA).^22^

In the HAT mechanism, the antioxidant molecule donates a hydrogen atom to neutralize HOO˙ free radical.^23^ This process is represented as:

4 HO-TA + HOO˙ → TAO˙ + HOOH

An essential parameter in this mechanism is the bond dissociation enthalpy (BDE),^24^ which reflects the strength of the bond broken during hydrogen donation. The BDE is calculated using the enthalpy of the hydrogen radical *H*(H˙), the enthalpy of the antioxidant radical *H*(TAO˙), and the enthalpy of the antioxidant molecule *H*(H-OTA), as expressed by the equation:^25^

5 BDE(H-OTA) = *H*(H˙) + *H*(TAO˙) − *H*(H-OTA)

In the PL mechanism, the antioxidant molecule donates a proton (H^+^), resulting in the formation of its anionic species. This process can be described as:^26^

6 H-OTA + HOO˙ → TAO^−^ + HOOH˙^+^

Proton affinity (PA) which relates to the PL mechanism is defined by the following equation:^27^

7 PA = *H*(TAO^−^)+ *H*(H^+^) − *H*(H-OTA)

SET mechanism involves a process, in which the antioxidant donates an electron to the free radical. This mechanism can be represented as follows:^28^

8 H-OTA + HOO˙ → H-OTA˙^+^ + HOO^−^

The key thermodynamic parameter associated with the single electron transfer mechanism is ionization potential (IP). This parameter is calculated using the following equation:

9 IP = *H*(H-OTA˙^+^) + *H*(e^−^) − *H*(H-OTA)

Here, *H* represents the enthalpy of each species at 298 K. The enthalpy values for the electron and proton in the gas phase are 0.75 kcal mol^−1^ and 1.48 kcal mol^−1^, respectively, whereas their corresponding values in water are −18.51 kcal mol^−1^ and −252.15 kcal mol^−1^.^29^

Additionally, the presence of multiple bonds in OH-TA molecules allows for radical adduct formation (RAF) to occur. This process typically takes place at carbon positions within the benzene and pyrrole rings. These reactions can be represented as follows:^30^

10 H-OTA + HOO˙ → [H-OTA-OOH]˙

The Gibbs free energy change (Δ*G*°) of a reaction serves as a critical parameter for assessing the feasibility of various reaction mechanisms. It can be determined using the following equations:^31^

11 Δ*G*°(HAT) = *G*(TAO˙) + *G*(HOOH) − *G*(HO-TA) − *G*(HOO˙)

12 Δ*G*°(SET) = *G*(H-OTA˙^+^) + *G*(HOO^−^) − *G*(H-OTA) − *G*(HOO˙)

13 Δ*G*°(PL) = *G*(TAO^−^) + *G*(HOOH˙^+^) − *G*(H-OTA) − *G*(HOO˙)

14 Δ*G*°(RAF) = *G*([H-OTA-OOH]˙) − *G*(H-OTA) − *G*(HOO˙)

Here, *G* denotes the Gibbs free energy of each species at 298.15 K.

The reaction kinetics between OH-TA derivatives and free radicals are analyzed by determining the rate constant (*k*) and the Gibbs free energy of activation (Δ*G*^‡^) using the Eyringpy software and transition state theory.^32^ Detailed calculations of the rate constants are presented in Table S1.†

## 3. Results and discussion

### 3.1. Stable conformation of OH-TAs

**Fig. 2:**
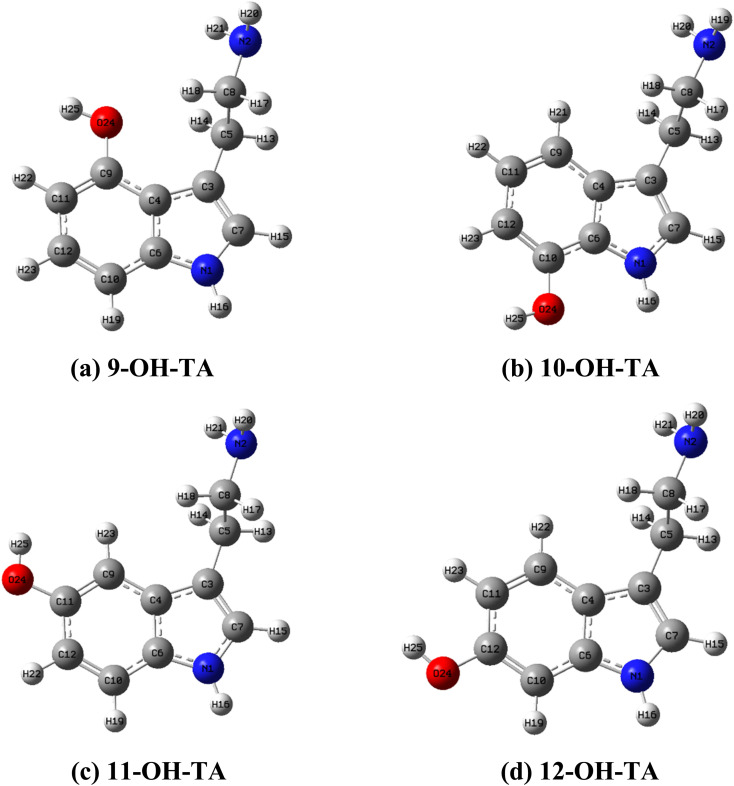
Optimized structures of (a) 9-OH-TA, (b) 10-OH-TA, (c) 11-OH-TA, and (d) 12-OH-TA in the gas phase.

Due to the rotational flexibility of their substituent groups, the OH-TAs can adopt various conformations. To identify the most stable structure, multiple OH-TA conformations will be optimized using the M06-2X functional with the 6-311++G(d,p) basis set. Fig. 2 shows the most stable gas phase structures of the OH-TA derivatives, with hydroxyl groups at different positions. These optimized structures are used for subsequent gas-phase analyses.

In aqueous environments, the protonation and deprotonation processes play a critical role in determining the molecular forms of OH-TA compounds. These molecules contain two acidic functional groups: an aliphatic amino group and an aromatic hydroxyl group. The distribution of these species in aqueous solutions at a given pH is governed by their p*K*_a_ values. The distribution of molecular forms was assessed by analyzing their proportions using calculated p*K*_a_ values (details in Fig. S1 and Table S2†).

**Fig. 3:**
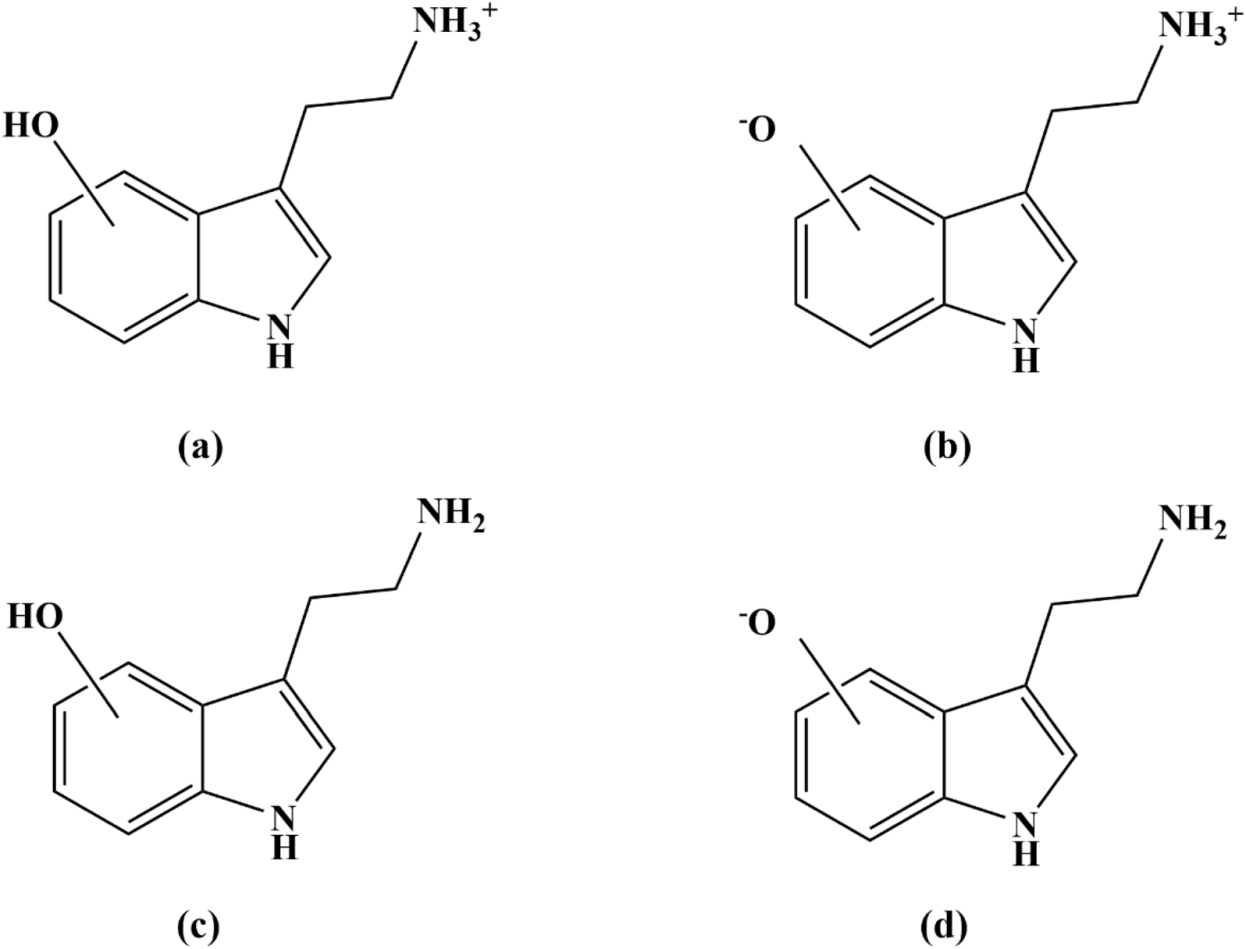
(a) Cationic, (b) zwitterionic, (c) neutral, and (d) anionic forms of OH-TA derivatives.

**Fig. 4:**
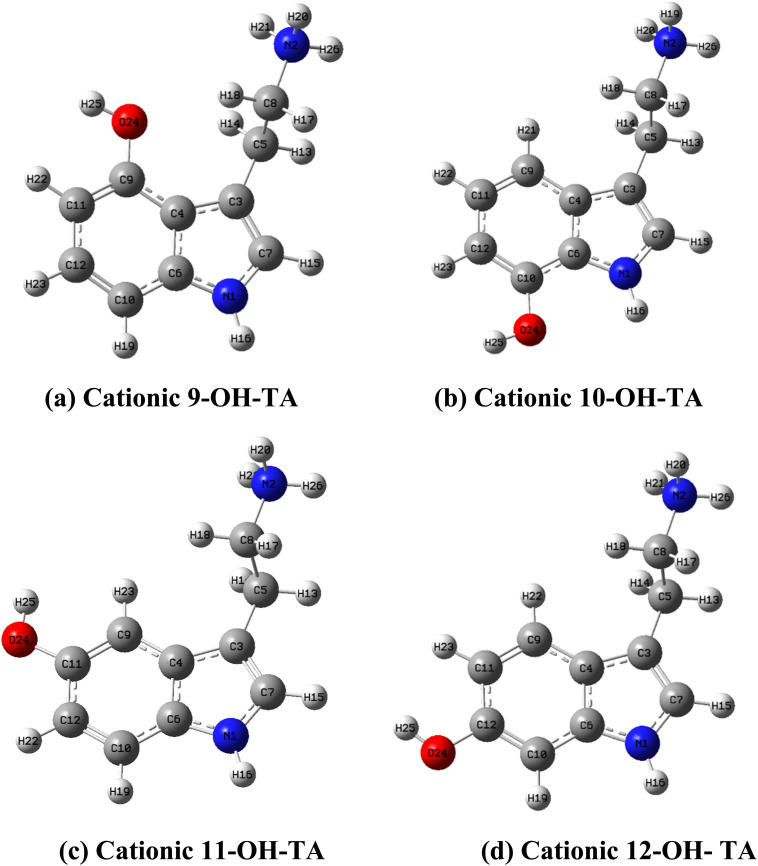
Optimized configurations of (a) cationic 9-OH-TA, (b) cationic 10-OH-TA, (c) cationic 11-OH-TA, and (d) cationic 12-OH-TA in water.

The analysis reveals that OH-TA derivatives can exist in four distinct forms – cationic, neutral, zwitterionic, and anionic – depending on the solution's pH (Fig. 3). Notably, at physiological pH (pH 7.4), the cationic form is predominant, comprising over 97% of the species. This finding aligns with previous studies on OH-TA derivatives.^33–35^ Consequently, the cationic form is commonly chosen for investigations into the properties of OH-TA compounds in aqueous environments (Fig. 4).

### 3.2. Frontier molecular orbital analysis

**Fig. 5:**
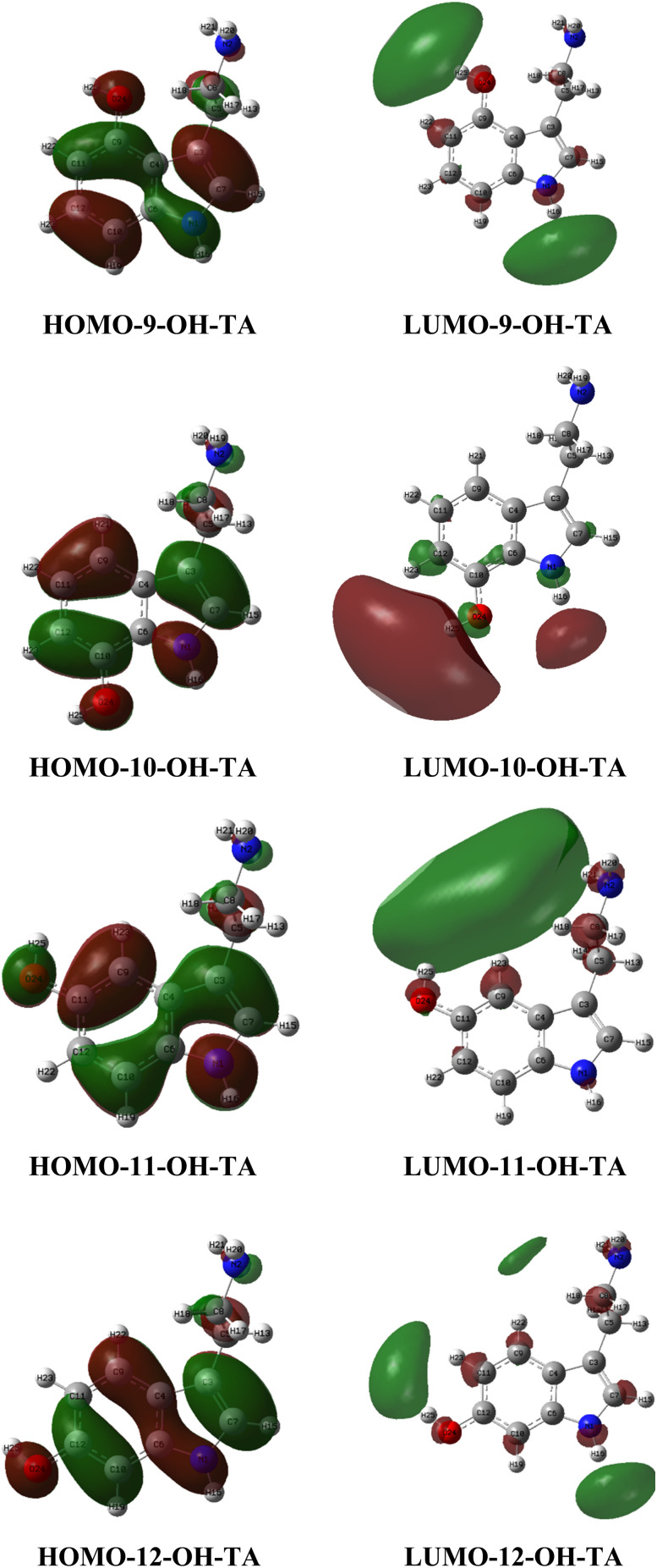
HOMO and LUMO plots of OH-TA derivatives in the gas phase.

The chemical reactivity of a molecule, especially in electron transfer processes, is primarily governed by its frontier molecular orbitals, which consist of the highest occupied molecular orbital (HOMO) and the lowest unoccupied molecular orbital (LUMO).^36^ The HOMO represents regions of high electron density, indicating the electron-donating sites of a molecule, while the LUMO highlights areas with low electron density, serving as potential electron-accepting sites. As illustrated in Fig. 5, the electron-donating regions of OH-TAs are primarily localized within the benzene and pyrrole rings. In contrast, the strongest electron-accepting regions are predominantly distributed across the hydroxyl (OH) and amine (NH) groups.

**Fig. 6:**
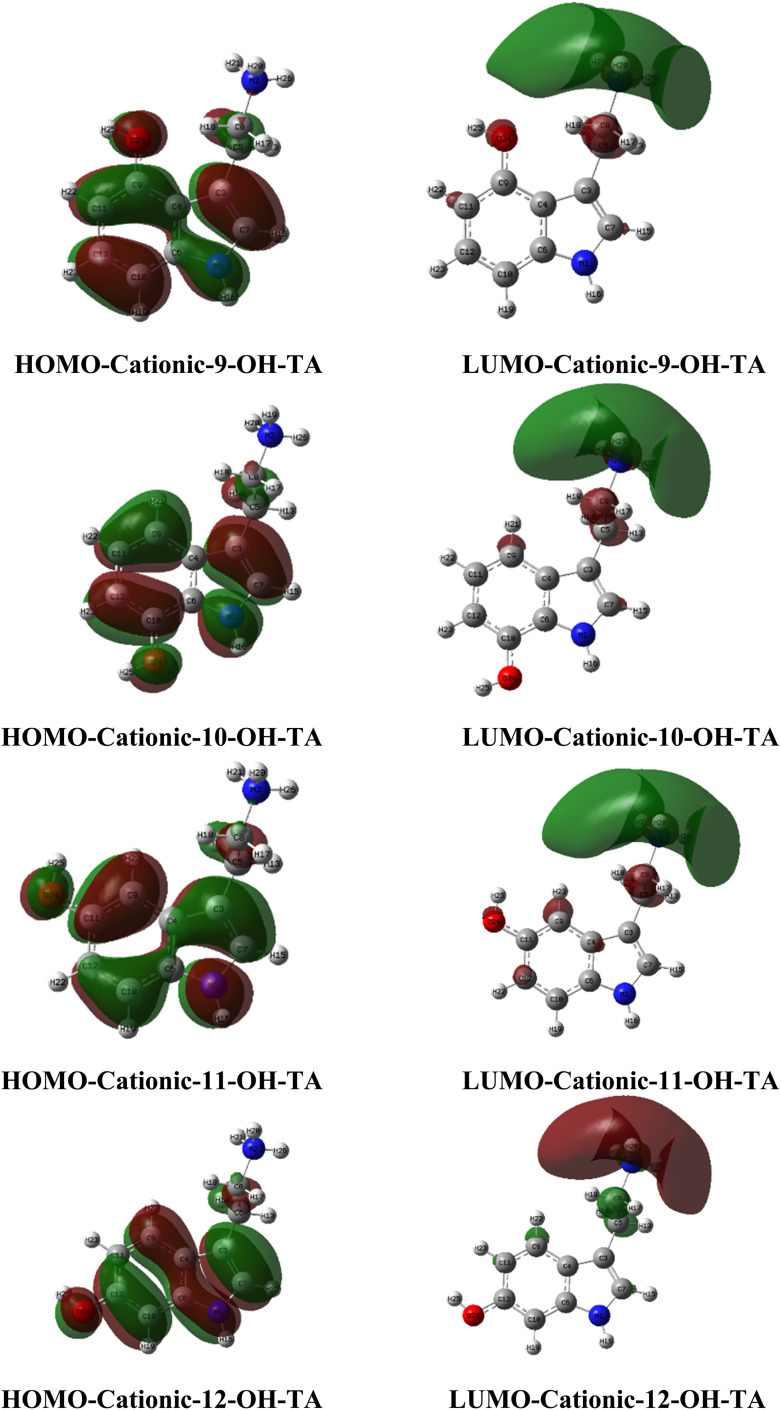
HOMO and LUMO visualization of OH-TAs in water.

In aqueous environments, the HOMO positions of OH-TAs exhibit less variability compared to their distribution in the gas phase. Conversely, the LUMOs of OH-TAs in water are predominantly localized at the NH_3_^+^ groups (Fig. 6).

Global reactivity descriptor parameters are quantitative measures used in theoretical studies to predict and understand the chemical reactivity of molecules.^37–41^ A comprehensive analysis of the global reactivity descriptor parameters is provided in Tables 1 and S3.† Among the investigated TA derivatives, 9-OH-TA is consistently identified as the most effective free radical scavenger.

**Table 1:** Global reactivity descriptor parameters of OH-TA derivatives at the M06-2X/6-311++G(d,p)

Parameters	Phase	9-OH-TA	10-OH-TA	11-OH-TA	12-OH-TA
*E*_HOMO_ (eV)	Gas	−6.7	−6.7	−6.9	−6.7
	Water	−6.8	−6.9	−7.0	−6.9
*E*_LUMO_ (eV)	Gas	−0.2	−0.2	−0.2	−0.2
	Water	−0.1	−0.1	−0.1	−0.1
Δ*E*_L–H_ (eV)	Gas	6.4	6.5	6.6	6.5
	Water	6.7	6.8	6.9	6.8
*η* (eV)	Gas	3.2	3.3	3.3	3.2
	Water	3.4	3.4	3.5	3.4
*χ* (eV)	Gas	3.4	3.5	3.6	3.4
	Water	3.5	3.5	3.6	3.5

### 3.3. Molecular electrostatic potential

**Fig. 7:**
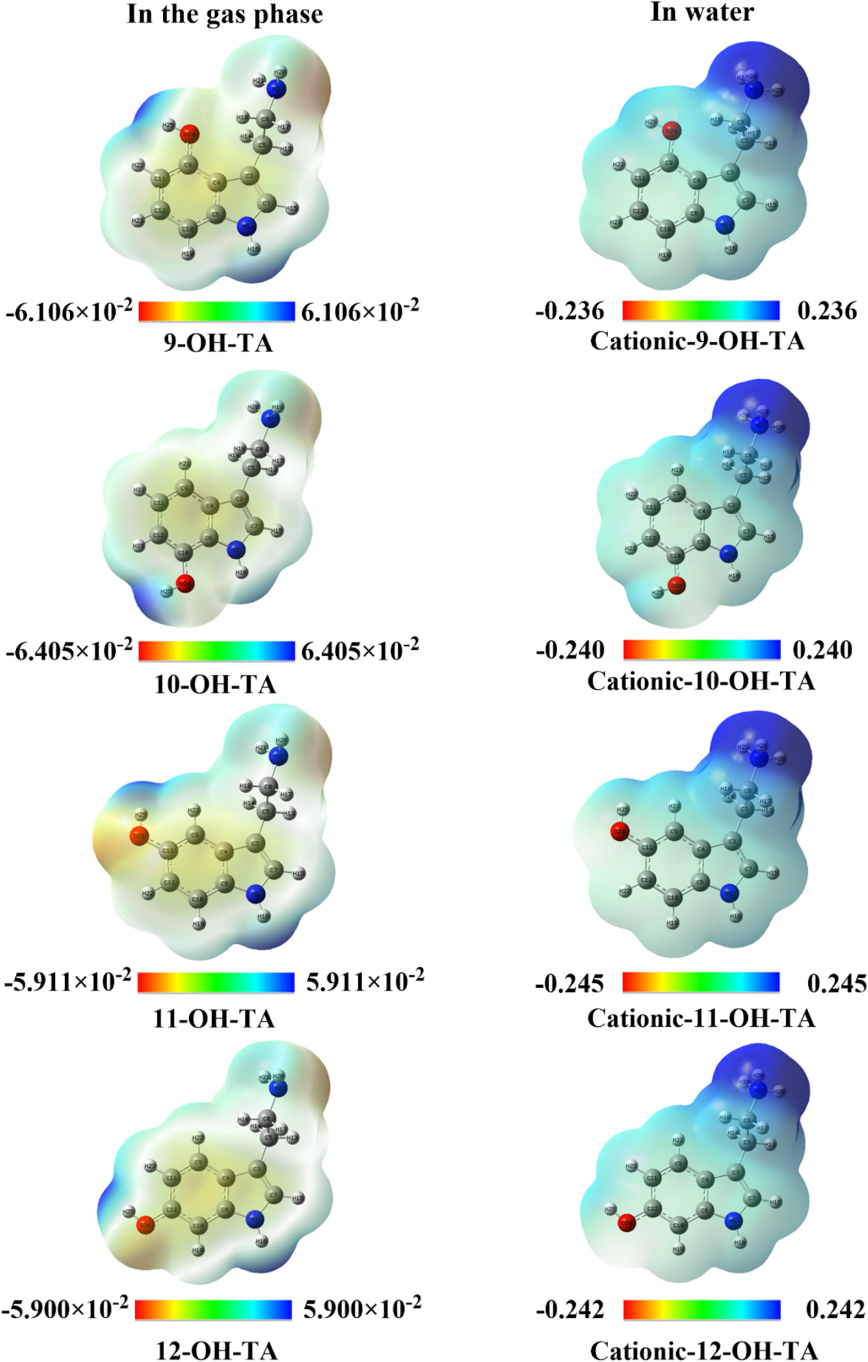
Molecular electrostatic potential of OH-TA derivatives in gas phase and water.

The molecular electrostatic potential (MEP) is a visualization of the electrostatic potential generated by the charge distribution within a molecule.^42^ Regions of negative electrostatic potential (depicted in red on MEP maps) are electron-rich and favorable for electrophilic attacks, while regions of positive potential (shown in blue) can attract nucleophilic species, facilitating radical-quenching mechanisms.^25^ MEP analysis is instrumental in elucidating the reactivity patterns of OH-TA derivatives in the gas phase and water (Fig. 7).

In Fig. 7, the blue regions on the MEP maps are predominantly localized around the hydroxyl (–OH) groups, indicating sites of significant electrophilic reactivity.^43^ These regions highlight the strongest electron-attracting tendencies and correspond to the hydrogen atom transfer sites, where OH-TA derivatives donate a hydrogen atom to stabilize radicals. The positive potential values for the blue regions are quantified as 6.106 × 10^−2^ a.u. for 9-OH-TA, 6.405 × 10^−2^ a.u. for 10-OH-TA, 5.911 × 10^−2^ a.u. for 11-OH-TA, and 5.900 × 10^−2^ a.u. for 12-OH-TA in the gas phase. These values suggest that the hydrogen atoms in the –OH groups of 9-OH-TA and 10-OH-TA are the most favorable sites for nucleophilic attacks.

In the aqueous phase (as shown in Fig. 7), the MEP maps of OH-TA derivatives reveal a shift in the positive potential region, which becomes concentrated around the NH_3_^+^ group. This shift indicates a preference for nucleophilic attack at this site in water. Furthermore, the increased blue region values for 11-OH-TA and 12-OH-TA suggest enhanced electrophilic activity in the NH_3_^+^ group under these conditions.

### 3.4. Thermodynamic parameters of OH-TA derivatives in the gas phase and water

Thermodynamic parameters are critical in evaluating the antioxidant properties of a compound, as they provide insights into the reaction mechanism and the compound's antioxidant efficacy.^44^ Depending on the mechanism under consideration, such as hydrogen atom transfer (HAT), single electron transfer (SET), or proton loss (PL), different thermodynamic parameters are considered.

In the HAT mechanism, the bond dissociation enthalpy (BDE) is a key determinant. Bonds with lower BDE values exhibit a greater propensity to donate hydrogen atoms, thereby enhancing the compound's antioxidant potential. For OH-TA derivative molecules, the O24–H, N1–H, and N2–H bonds are identified as having strong hydrogen-donating abilities. Consequently, the BDE values of these bonds are calculated in both gas and aqueous phases to assess their reactivity under varying environmental conditions.

As presented in Table 2, the BDE(O24–H) values range from 79.9 to 83.0 kcal mol^−1^, whereas the BDE(N1–H) values are higher, ranging from 89.3 to 93.3 kcal mol^−1^, and the BDE(N2–H) is 98.2 kcal mol^−1^ in the gas phase. This indicates that the O24–H bonds consistently exhibit lower BDEs compared to the N1–H and N2–H bonds in the gas phase.

**Table 2:** BDE, IP, and PA values of OH-TA derivatives in the gas phase and water

Compound	Position	Gas			Water		
		BDE	IP	PA	BDE	IP	PA
9-OH-TA	O24–H	80.8	168.0	342.6	81.3	107.2	36.1
	N1–H	89.3		347.0	90.4		43.2
	N2–H	98.2		399.0	95.1		29.7
10-OH-TA	O24–H	79.9	170.8	342.5	81.9	109.8	35.2
	N1–H	93.3		349.3	92.0		42.8
	N2–H	98.2		395.2	97.5		29.3
11-OH-TA	O24–H	83.0	173.5	351.2	83.2	110.8	39.1
	N1–H	91.2		346.4	92.5		43.7
	N2–H	98.2		393.4	98.0		29.3
12-OH-TA	O24–H	82.4	167.4	347.7	82.4	106.5	38.0
	N1–H	89.3		347.6	90.1		44.1
	N2–H	98.2		394.3	94.1		29.3

In aqueous environments, the BDE values for O24–H and N1–H bonds tend to increase, while the BDE of the N2–H bond decreases. The BDE(N2–H) values for OH-TA derivatives in water range from 94.1 to 98.0 kcal mol^−1^. Despite the reduction in BDE for N2–H in water, the BDE (O24–H) remains significantly lower, ranging from 81.3 to 83.2 kcal mol^−1^. Among the derivatives, 9-OH-TA and 10-OH-TA have the lowest BDE(O24–H) values, at 81.3 and 81.9 kcal mol^−1^. These findings suggest that under the HAT mechanism, 9-OH-TA and 10-OH-TA are more capable of donating hydrogen atoms from the O24–H position than other derivatives, underscoring their superior antioxidant potential at this site.

For the SET mechanism, the ionization potential (IP) is a key parameter to evaluate.^45^ In the gas phase, the IP values of 9-OH-TA, 10-OH-TA, 11-OH-TA, and 12-OH-TA are 168.0, 170.8, 173.5, and 167.4 kcal mol^−1^, respectively. These values decrease significantly in the aqueous phase, where they are 107.2, 109.8, 110.8, and 106.5 kcal mol^−1^, respectively. Among the derivatives, 9-OH-TA and 12-OH-TA exhibit the lowest IP values in both gas and aqueous phases, indicating that these compounds are more inclined to donate electrons compared to the others. This suggests that 9-OH-TA and 12-OH-TA possess enhanced electron-donating abilities, which may contribute to their antioxidant activity through the SET mechanism.

Proton affinity (PA) is a critical parameter for evaluating the proton loss (PL) mechanism, where a lower PA value indicates a higher propensity for proton donation.^46^ As presented in Table 2 in the gas phase, the PA values for 9-OH-TA are 342.6; 347.0, and 399.0 kcal mol^−1^, corresponding to the O24–H, N1–H, and N2–H bonds, respectively. For 10-OH-TA, the PA values are 342.5; 349.3, and 395.2 kcal mol^−1^, while for 11-OH-TA, the values are 351.2; 346.4, and 393.4 kcal mol^−1^ for the same bonds. Similarly, 12-OH-TA shows PA values of 347.7; 347.6, and 394.3 kcal mol^−1^ for the O24–H; N1–H, and N2–H bonds, respectively. In an aqueous environment, the PA values decrease significantly, ranging from 302.7 to 306.5 kcal mol^−1^ for the N1–H bonds, from 364.0 to 369.3 kcal mol^−1^ for the N2–H bonds, and from 306.4 to 312.1 kcal mol^−1^ for the O24–H bonds. This substantial reduction in PA values indicates that the PL mechanism is more favorable in an aqueous environment for OH-TA derivatives, enhancing their proton-donating capabilities.

### 3.5. HOO˙ free radical scavenging activity

The OH-TA derivatives are evaluated for their free radical scavenging activity against the hydroperoxyl radical (HOO˙) using multiple mechanisms: HAT, SET, PL, and RAF. The Gibbs free energy change (Δ*G*°) is used as the criterion to determine the feasibility of these mechanisms (Table 3).

**Table 3:** Δ*G*° values of reactions according to different mechanisms

Mechanism	Position	9-OH-TA		10-OH-TA		11-OH-TA		12-OH-TA	
		Gas	Water	Gas	Water	Gas	Water	Gas	Water
HAT	N1–H	3.9	0.8	7.7	2.3	5.6	2.6	3.7	0.3
	N2–H	13.0	5.3	12.9	7.7	12.9	9.0	12.8	4.5
	O24–H	−4.5	−8.3	−5.3	−7.3	−2.1	−6.4	−2.6	−6.8
SET		145.0	20.4	147.8	22.9	150.8	23.8	144.5	19.6
PL	N1–H	194.8	59.9	197.0	59.7	194.3	60.3	195.2	60.6
	N2–H	245.6	46.4	242.4	45.8	240.9	46.1	241.8	46.1
	O24–H	191.0	52.9	190.8	52.4	199.4	56.1	195.5	55.0
RAF	C7	3.3	291.2	4.8	287.4	3.7	309.1	2.3	285.5
	C9	8.5	279.0	11.1	294.1	7.5	293.3	10.8	294.2
	C10	12.3	287.7	9.3	293.7	12.3	295.6	10.8	295.2
	C11	16.4	292.2	17.2	300.2	13.6	318.6	15.0	300.2
	C12	15.4	295.6	14.0	298.4	15.5	297.4	11.7	13.3

For the HAT mechanism, potential reaction sites include N1–H, N2–H, and O24–H. At the N1–H site, Δ*G*° values range from 3.7 to 7.7 kcal mol^−1^ in the gas phase and 0.3 to 2.6 kcal mol^−1^ in water, indicating non-spontaneous reactions. Similarly, at the N2–H site, Δ*G*° values are 12.8–13.0 kcal mol^−1^ in the gas phase and 4.5–9.0 kcal mol^−1^ in water, confirming the non-spontaneity of these reactions. In contrast, reactions at the O24–H position exhibit negative Δ*G*° values for 9-OH-TA, 10-OH-TA, 11-OH-TA, and 12-OH-TA, with values of −4.5, −5.3, −2.1, and −2.6 kcal mol^−1^, respectively, in the gas phase, and −8.3, −7.3, −6.4, and −6.8 kcal mol^−1^ in water. These results indicate that reactions at the O24–H position are thermodynamically favorable in both phases. Among the derivatives, 10-OH-TA is the most favorable in the gas phase, while 9-OH-TA is the most favorable in water.

For the SET mechanism, Δ*G*° values range from 144.5 to 150.8 kcal mol^−1^ in the gas phase and 19.6 to 23.8 kcal mol^−1^ in water. Although the values are significantly lower in water, the reactions remain non-spontaneous due to positive Δ*G*° values.

Under the PL mechanism, the proton transfer from OH-TA to HOO˙ results in the formation of the anionic form of OH-TA and HOOH˙^+^ in the gas phase. This mechanism is investigated at the N1–H, N2–H, and O24–H positions. Δ*G*° values at N1–H range from 194.3 to 197.0 kcal mol^−1^, at N2–H from 240.9 to 245.6 kcal mol^−1^, and at O24–H from 190.8 to 199.4 kcal mol^−1^ in the gas phase. In aqueous solution, the Δ*G*° values become less positive, indicating a slight thermodynamic improvement. Nonetheless, the Δ*G*° values confirm that the proton transfer reaction remains thermodynamically unfavorable in both solvents.

Lastly, for the RAF mechanism, addition reactions at positions C7, C9, C10, C11, and C12 are considered. While Δ*G*° values are higher in water compared to the gas phase, they remain positive across both environments, suggesting that reactions *via* the RAF mechanism are not thermodynamically favorable.

### 3.6. Reaction kinetics of OH-TA derivatives with HOO˙ free radicals according to hydrogen atom transfer mechanism in the gas phase

**Fig. 8:**
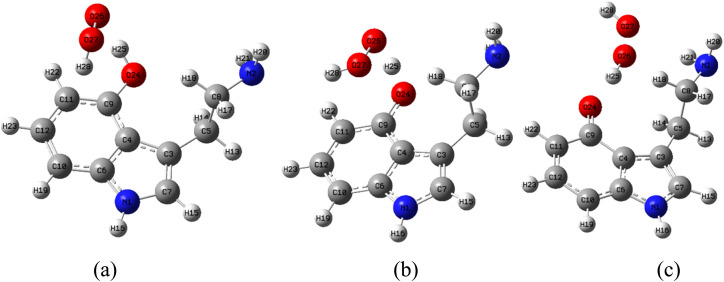
(a) Inter 1, (b) TS, (c) Inter 2 in the HAT reaction between 9-OH-TA and HOO˙ in the gas phase.

The kinetics of the hydrogen atom transfer (HAT) reaction between OH-TA derivatives and HOO˙ radicals at the O24–H bond are investigated in the gas phase due to the negative values of Δ*G*°. In the HAT mechanism, the reaction pathway involves the formation of a first intermediate state (Inter 1) *via* a transition state (TS), followed by the generation of a second intermediate (Inter 2) and culminating in the final product. Representative structures of the species participating in the reaction with free radicals at the O24–H bond in the gas phase *via* the HAT mechanism are depicted in Fig. 8.

**Fig. 9:**
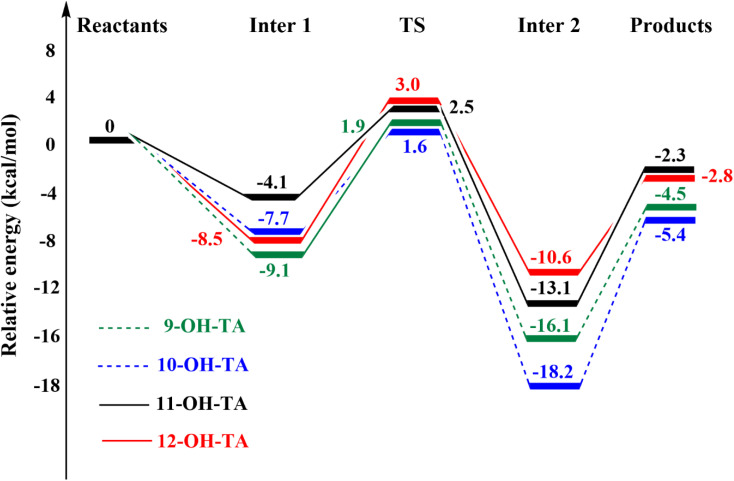
PES of HAT reactions between OH-TAs and HOO˙ in the gas phase.

The potential energy surfaces (PES) of the reactions between OH-TA derivatives and HOO˙ free radicals in the gas phase are illustrated in Fig. 9. Initially, the antioxidant compounds react with HOO˙ free radicals to form the Inter 1, with relative energy values (compared to the reactants) of −9.1, −7.7, −4.1, and −8.5 kcal mol^−1^ for 9-OH-TA, 10-OH-TA, 11-OH-TA, and 12-OH-TA in the gas, respectively. The reactions then proceed through transition states with relative energy values of 1.9, 1.6, 2.5, and 3.0 kcal mol^−1^, corresponding to the same derivatives. Subsequently, the Inter 2 is formed with energy values of −16.1, −18.2, −13.1, and −10.6 kcal mol^−1^ for 9-OH-TA, 10-OH-TA, 11-OH-TA, and 12-OH-TA, respectively. The reactions ultimately yield products consisting of antioxidant-derived free radicals and HOOH.

Key kinetic parameters, including the Gibbs free energy of activation (Δ*G*^‡^) and rate constants (*k*), are computed using conventional transition state theory (TST) under standard state conditions of 1 M and a temperature of 298.15 K.^47^ The results of these kinetic studies are summarized in Table 4.

**Table 4:** Kinetic results of HAT reactions at O24–H bond for OH-TA derivatives in the gas phase

Compound	*k* (M^−1^ s^−1^)	Δ*G*^‡^ (kcal mol^−1^)	Eck
9-OH-TA	1.9 × 10^7^	11.2	114.3
10-OH-TA	3.5 × 10^6^	11.0	16.2
11-OH-TA	1.9 × 10^6^	11.8	31.5
12-OH-TA	1.3 × 10^7^	11.5	148.8

In the gas phase, the activation Gibbs free energies for the reactions between OH-TA derivatives and HOO˙ *via* the HAT mechanism ranged from 11.0 to 11.8 kcal mol^−1^, while the corresponding Eckart transmission coefficients varied between 16.2 and 148.8. Among the compounds studied, 9-OH-TA exhibited the highest rate constant, 1.9 × 10^7^ M^−1^ s^−1^, followed by 12-OH-TA (1.3 × 10^7^ M^−1^ s^−1^), 10-OH-TA (3.5 × 10^6^ M^−1^ s^−1^), and 11-OH-TA (1.9 × 10^6^ M^−1^ s^−1^). These results indicate that, in the gas phase, 9-OH-TA reacts most rapidly with the HOO˙ radical at the O24–H bond *via* the HAT mechanism.

### 3.7. Reaction kinetics of OH-TA derivatives with HOO˙ free radicals in water

**Fig. 10:**
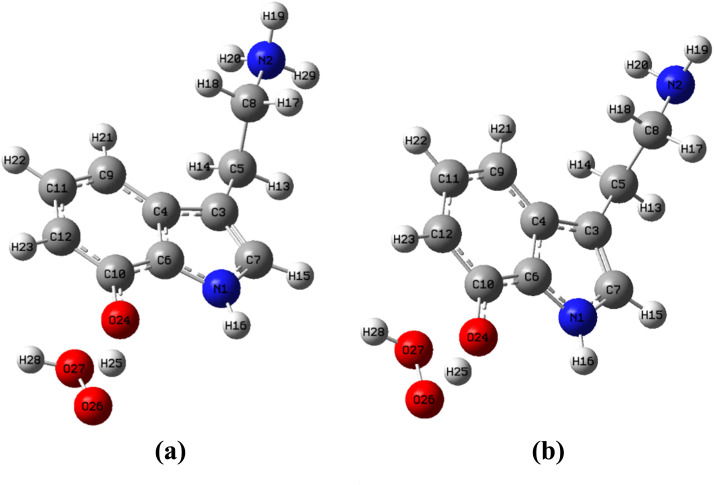
(a) TS of cationic form, (b) TS of neutral form in the HAT reaction between 10-OH-TA and HOO˙ in water.

In an aqueous environment, two reaction mechanisms are explored: the hydrogen atom transfer (HAT) mechanism and the single electron transfer (SET) mechanism. For the HAT mechanism, two forms of OH-TA derivatives – cationic and neutral – are examined in their reactions with the HOO˙ radical, as these derivatives possess active O24–H bonds. The representative transition state (TS) structures associated with the reaction at the O24–H bond *via* the HAT mechanism in water are depicted in Fig. 10.

The rate constants for OH-TA derivatives reacting with HOO˙ *via* the HAT mechanism (*k*_HAT_) in water are calculated using the following equation:^48^

15 *k*_HAT_ = *k*_(HAT-cationic)_ + *k*_(HAT-neutral)_ = *f*_cationic_ × *k*_app(HAT-cationic)_ + *f*_neutral_ × *k*_app(HAT-neutral)_

Here, *f* and *k*_app_ represent the mole fraction and rate constant of the various forms of OH-TA derivatives in water.

As can be seen in Table 5, the rate constant values calculated for the HAT mechanism indicate that the cationic forms of OH-TA derivatives exhibit higher rate constants than their neutral species when reacting with HOO˙. Given the high molar ratio of the cationic form, it is the dominant contributor to the HAT reaction rate in water. At pH 7.4, the HAT rate constants (*k*_HAT_) for 9-OH-TA, 10-OH-TA, 11-OH-TA, and 12-OH-TA are 6.2 × 10^6^, 1.8 × 10^5^, 8.4 × 10^4^, and 3.6 × 10^5^ M^−1^ s^−1^, respectively.

**Table 5:** Kinetic results of HAT reactions at O24–H bond for OH-TA derivatives in water

Compound	Form	*f*	*k*_app(HAT)_ (M^−1^ s^−1^)	*k*_(HAT)_ (M^−1^ s^−1^)	*k*_HAT_ = Σ*k*_(HAT)_ (M^−1^ s^−1^)
9-OH-TA	Cationic	0.9785	6.3 × 10^6^	6.2 × 10^6^	6.2 × 10^6^
	Neutral	0.0045	6.0 × 10^6^	2.7 × 10^4^	
10-OH-TA	Cationic	0.989	1.8 × 10^5^	1.8 × 10^5^	1.8 × 10^5^
	Neutral	0.0041	1.7 × 10^5^	7.0 × 10^2^	
11-OH-TA	Cationic	0.9882	8.5 × 10^4^	8.4 × 10^4^	8.4 × 10^4^
	Neutral	0.0041	8.0 × 10^4^	3.3 × 10^2^	
12-OH-TA	Cationic	0.9881	3.6 × 10^5^	3.6 × 10^5^	3.6 × 10^5^
	Neutral	0.0043	3.4 × 10^5^	1.5 × 10^3^	

In the single electron transfer (SET) mechanism, all four forms of OH-TA derivatives in water − cationic, neutral, zwitterionic, and anionic − are assessed for their electron-donating abilities in reactions with the HOO˙ radical. The SET rate constant (*k*_SET_) for the investigated compounds is determined using the following equation:^49^

16 *k*_SET_ = *k*_(SET-cationic)_ + *k*_(SET-neutral)_ + *k*_(SET-zwitterionic)_ + *k*_(SET-anionic)_ = *f*_cationic_ × *k*_app(SET-cationic)_ + *f*_neutral_ × *k*_app(SET-neutral)_ + *f*_zwitterionic_ × *k*_app(SET-zwitterionic)_ + *f*_anionic_ × *k*_app(SET- anionic)_

The calculated rate constants for the reactions between different forms of OH-TA derivatives and the HOO˙ radical are summarized in Table 6. As shown in the table, although the cationic form constitutes the majority (over 97%) of the species in water, its rate constant with the HOO˙ radical *via* the SET mechanism is nearly negligible. While the anionic form exhibits a relatively high rate constant for the SET mechanism, OH-TA derivatives are virtually absent in this form at pH 7.4. The zwitterionic form plays a crucial role in determining the free radical scavenging activity of OH-TA derivatives due to its electron-donating capacity. The SET rate constants (*k*_SET_) for the reaction with HOO˙ are 4.6 × 10^7^, 1.7 × 10^7^, 2.0 × 10^7^, and 2.1 × 10^7^ M^−1^ s^−1^ for 9-OH-TA, 10-OH-TA, 11-OH-TA, and 12-OH-TA, respectively. Among the studied derivatives, 9-OH-TA demonstrates the most efficient electron-donating ability in water.

**Table 6:** Rate constants (*k*_SET_ and *k*_overall_) of the reactions between OH-TA derivatives with HOO˙

Compound	Form	*f*	*k*_app(SET)_ (M^−1^ s^−1^)	*k*_(SET)_ (M^−1^ s^−1^)	*k*_SET_ = Σ*k*_(SET)_ (M^−1^ s^−1^)	*k*_overall_ (M^−1^ s^−1^)
9-OH-TA	Cationic	0.9785	4.8 × 10^−3^	4.7 × 10^−3^	4.6 × 10^7^	5.2 × 10^7^
	Zwitterionic	0.0169	2.7 × 10^9^	4.6 × 10^7^		
	Neutral	0.0045	8.4 × 10^−1^	3.8 × 10^−3^		
	Anionic	0.0001	2.7 × 10^9^	2.7 × 10^5^		
10-OH-TA	Cationic	0.9890	2.8 × 10^−5^	2.8 × 10^−5^	1.7 × 10^7^	1.7 × 10^7^
	Zwitterionic	0.0069	2.5 × 10^9^	1.7 × 10^7^		
	Neutral	0.0041	6.2 × 10^−3^	2.5 × 10^−5^		
	Anionic	0.0000	2.6 × 10^9^	0.0		
11-OH-TA	Cationic	0.9882	6.7 × 10^−6^	6.6 × 10^−6^	2.0 × 10^7^	2.0 × 10^7^
	Zwitterionic	0.0077	2.6 × 10^9^	2.0 × 10^7^		
	Neutral	0.0041	7.4 × 10^−4^	3.0 × 10^−6^		
	Anionic	0.0000	2.7 × 10^9^	0.0		
12-OH-TA	Cationic	0.9881	2.6 × 10^−2^	2.6 × 10^−2^	2.1 × 10^7^	2.1 × 10^7^
	Zwitterionic	0.0076	2.7 × 10^9^	2.1 × 10^7^		
	Neutral	0.0043	2.7	1.2 × 10^−2^		
	Anionic	0.0000	2.7 × 10^9^	0.0		

Based on the calculated values of *k*_HAT_ and *k*_SET_, the total rate constant (*k*_overall_) for the reactions of the studied OH-TA derivatives with the HOO˙ radical in water is determined using the following equation:^50^

17 *k*_overall_ = *k*_SET_ + *k*_HAT_

At pH 7.4 in water, the total rate constants for 9-OH-TA, 10-OH-TA, 11-OH-TA, and 12-OH-TA are calculated to be 5.2 × 10^7^, 1.7 × 10^7^, 2.0 × 10^7^, and 2.1 × 10^7^ M^−1^ s^−1^, respectively. Among these, 9-OH-TA exhibits the highest efficiency as a scavenger of the HOO˙ radical.

In the gas phase, the reaction between the studied derivatives and the HOO˙ radical predominantly follows the HAT mechanism. However, in water, the reaction primarily proceeds *via* the SET mechanism.

## 4. Conclusions

Density functional theory was used to investigate the free radical scavenging activity of hydroxyl-substituted tryptamine derivatives. The analysis included frontier molecular orbital analysis, molecular electrostatic potential mapping, thermodynamic calculations, and an investigation of reaction mechanisms. The findings revealed that the position of the hydroxyl group significantly influenced the free radical scavenging efficiency of OH-TAs. In the gas phase, 9-OH-TA, 10-OH-TA, 11-OH-TA, and 12-OH-TA effectively scavenged HOO˙ radicals *via* the hydrogen atom transfer mechanism, with rate constants of 1.9 × 10^7^ M^−1^ s^−1^, 3.5 × 10^6^ M^−1^ s^−1^, 1.9 × 10^6^ M^−1^ s^−1^, 1.3 × 10^7^ M^−1^ s^−1^, respectively. In contrast, in water, these derivatives predominantly react with free radicals *via* the single electron transfer (SET) mechanism in their zwitterionic forms. Among the derivatives, 9-OH-TA exhibits the highest HOO˙ radical scavenging efficiency in polar media, with a rate constant of 5.2 × 10^7^ M^−1^ s^−1^.

## Data availability

The data supporting this article have been included as part of the ESI.†

## Author contributions

Dinh Quy Huong: conceptualization, methodology, validation, data curation, formal analysis, visualization, writing – original draft, writing – review & editing. Duong Tuan Quang: supervision, writing – review & editing. Le Quoc Thang: conceptualization, methodology. Nhan Thi Thanh Dang: validation, data curation. Nguyen Le My Linh: formal analysis, visualization. Nguyen Minh Thong: data curation, formal analysis, visualization. Nguyen Minh Tam: software, writing – review & editing. Quan V. Vo: software, writing – review & editing. Pham Cam Nam: supervision, writing – review & editing.

## Conflicts of interest

There are no conflicts of interest to declare.

## Supplementary Material

RA-015-D5RA01364J-s001
