# Supplementary material for: Theoretical study on the influence of OH group position on the free radical scavenging ability of tryptamine derivatives
Source: RSC Adv. 2025 Apr 11;15(15):11417–30. doi: 10.1039/d5ra01364j (PMC11986808; doi:10.1039/d5ra01364j)
Supplement: RA-015-D5RA01364J-s001 [file RA-015-D5RA01364J-s001.pdf]

Supplementary Information (SI) for RSC Advances.
This journal is © The Royal Society of Chemistry 2025

Supplementary Data File

Theoretical study on the influence of OH group position on the free radical scavenging ability of Tryptamine derivatives

Dinh Quy Huong,[*a] Duong Tuan Quang,[a] Le Quoc Thang,[a] Dang Thi Thanh Nhan,[a] Nguyen Le My Linh,[a] Nguyen Minh Thong,[b] Nguyen Minh Tam,[c] Quan V. Vo,[d] Pham Cam Nam[e]

[a]Department of Chemistry, University of Education, Hue University, Hue, Vietnam.

[b]The University of Danang-University of Science and Education, Danang, Vietnam.

[c]Faculty of Basic Sciences, University of Phan Thiet, 225 Nguyen Thong, Phan Thiet City, Binh Thuan, Vietnam

[d]Faculty of Chemical Technology-Environment, The University of Danang-University of Technology and Education, Danang, Vietnam

[e]Department of Chemical Engineering, The University of Danang – University of Science and Technology, Danang, Vietnam.

*: Corresponding author: dqhuong@hueuni.edu.vn

List of supporting information

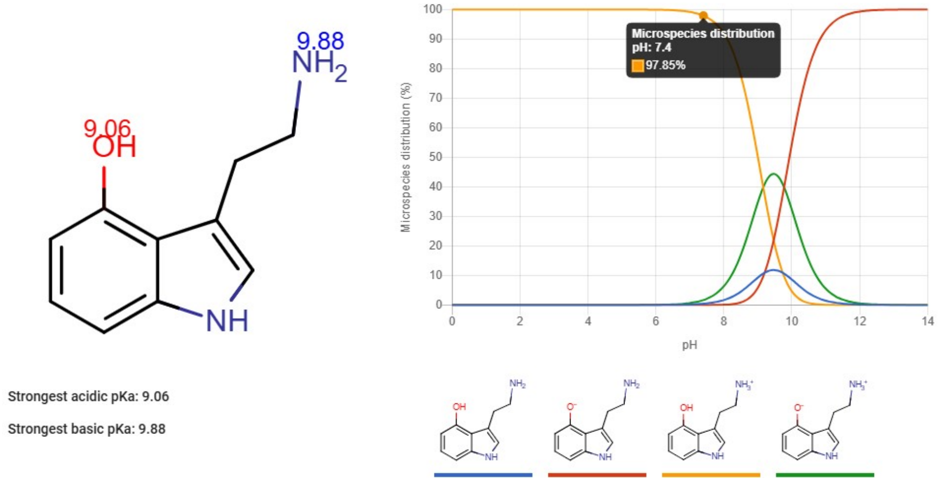

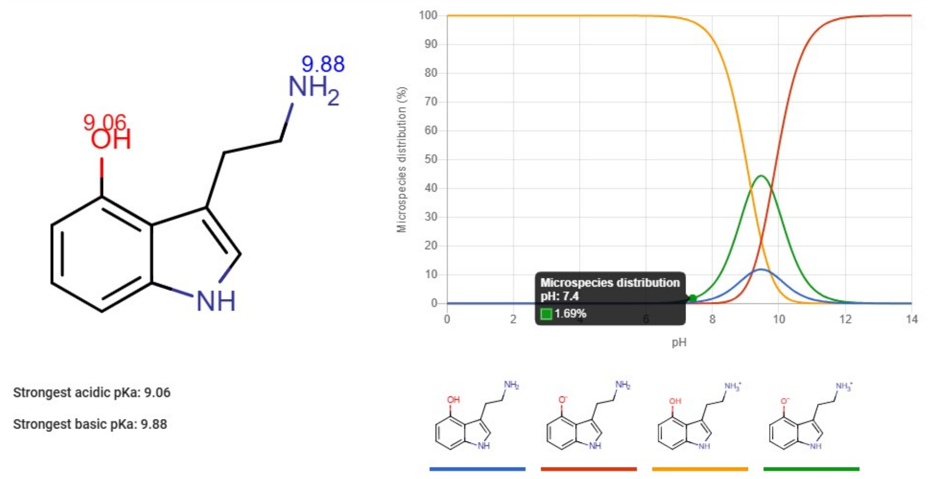
9.88
9.06
Microspecies distribution
pH: 7.4
1.69%
Microspecies distribution (%)
pH
Strongest acidic pKa: 9.06
Strongest basic pKa: 9.88

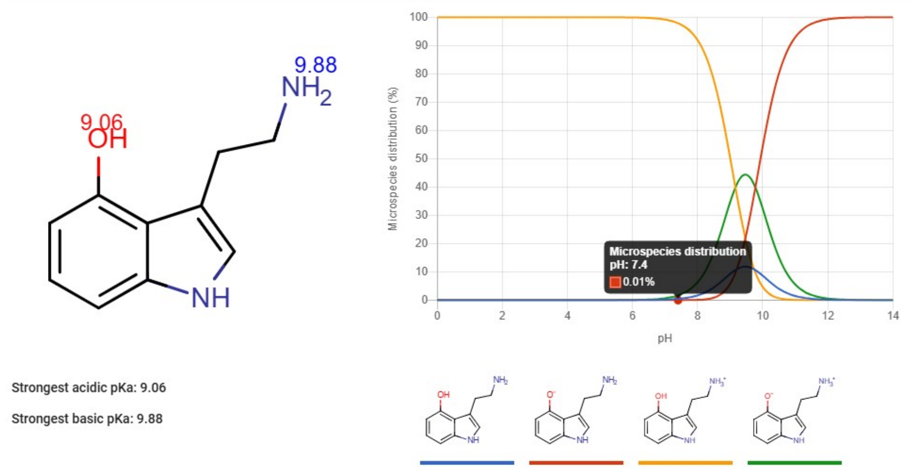
9.88
9.06
Microspecies distribution
pH: 7.4
0.01%
Microspecies distribution (%)
pH
Strongest acidic pKa: 9.06
Strongest basic pKa: 9.88

(a) 9-OH-TA

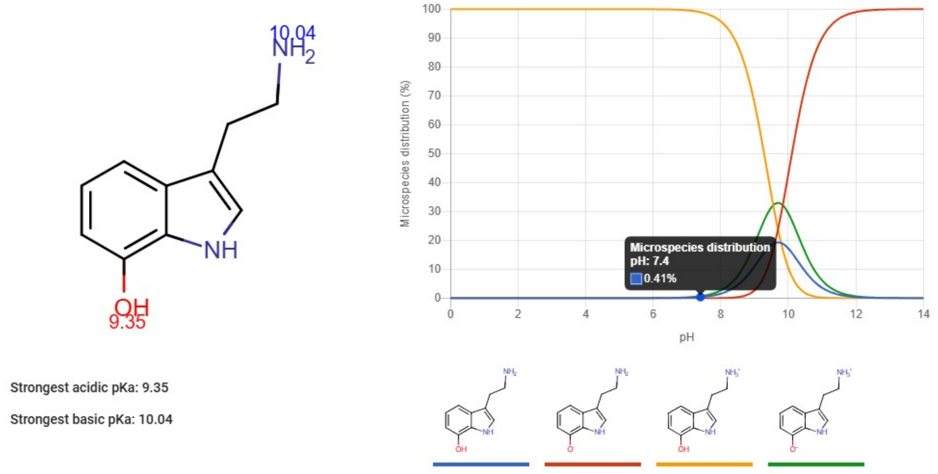
10.04
9.35
Microspecies distribution
pH: 7.4
0.41%
Microspecies distribution (%)
pH
Strongest acidic pKa: 9.35
Strongest basic pKa: 10.04

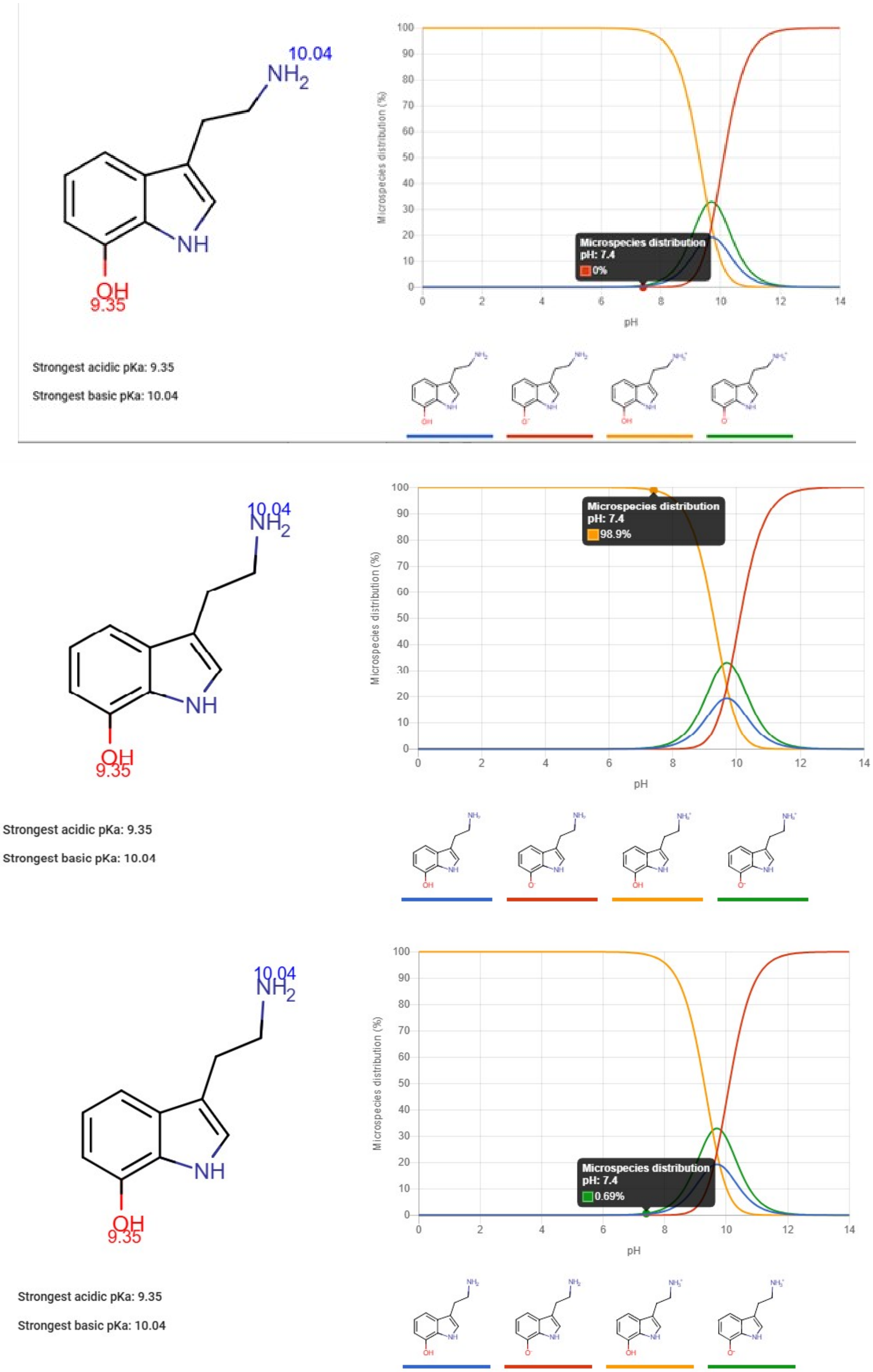
10.04
NH2
NH
OH
9.35
Microspecies distribution (%)
pH
Microspecies distribution
pH: 7.4
0%
Strongest acidic pKa: 9.35
Strongest basic pKa: 10.04
Microspecies distribution
pH: 7.4
98.9%
Strongest acidic pKa: 9.35
Strongest basic pKa: 10.04
Microspecies distribution
pH: 7.4
0.69%
Strongest acidic pKa: 9.35
Strongest basic pKa: 10.04

(b) 10-OH-TA

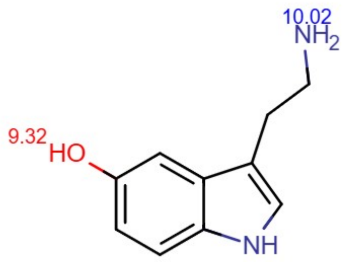

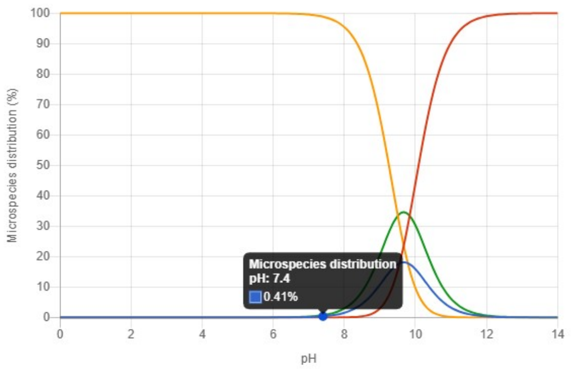

Strongest acidic pKa: 9.32

Strongest basic pKa: 10.02

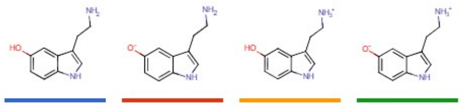

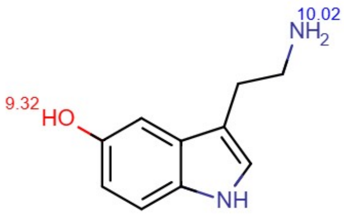

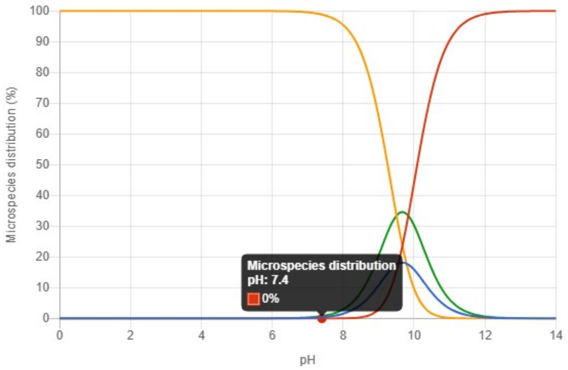

Strongest acidic pKa: 9.32

Strongest basic pKa: 10.02

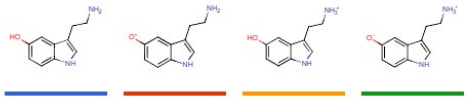

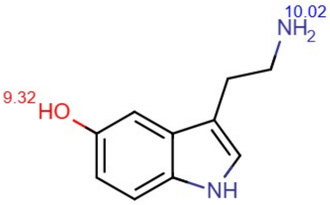

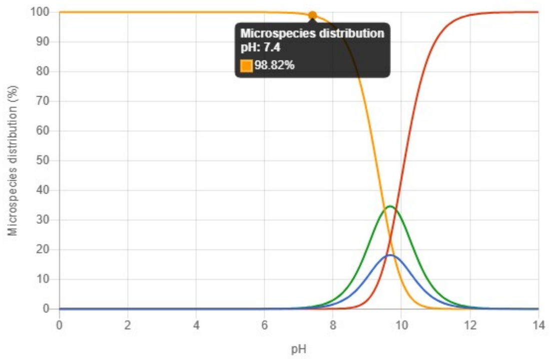

Strongest acidic pKa: 9.32

Strongest basic pKa: 10.02

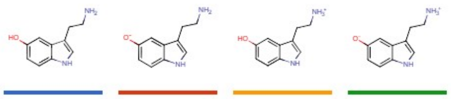

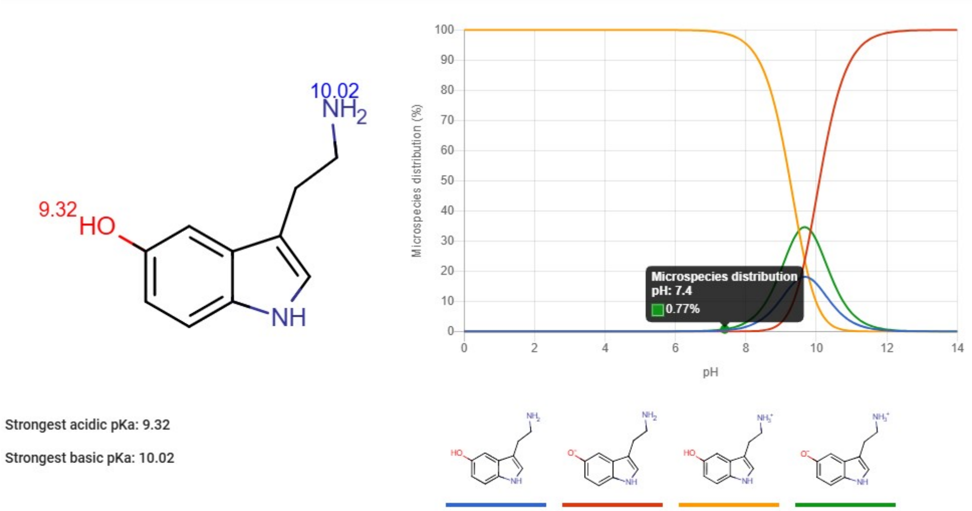
10.02
9.32
Microspecies distribution (%)
pH
Microspecies distribution
pH: 7.4
0.77%
Strongest acidic pKa: 9.32
Strongest basic pKa: 10.02

(c) 11-OH-TA

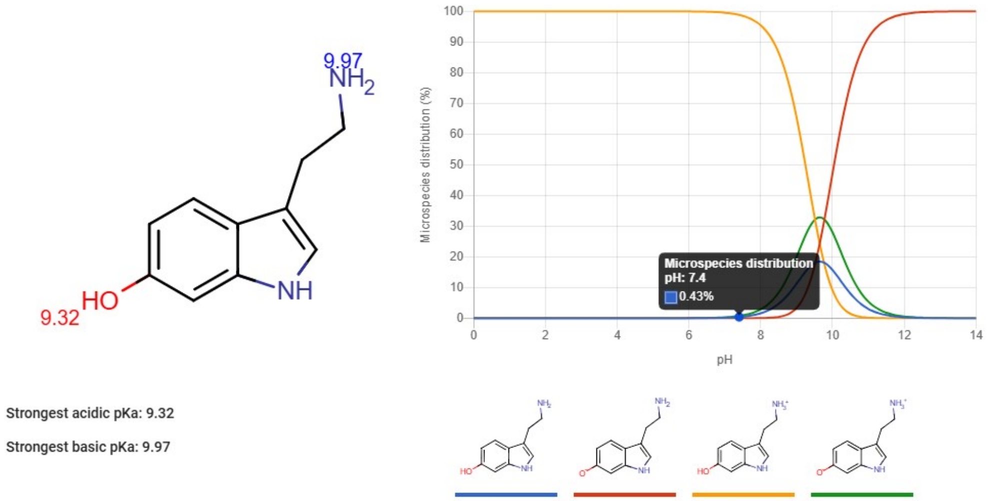
9.97
9.32
Microspecies distribution (%)
pH
Microspecies distribution
pH: 7.4
0.43%
Strongest acidic pKa: 9.32
Strongest basic pKa: 9.97

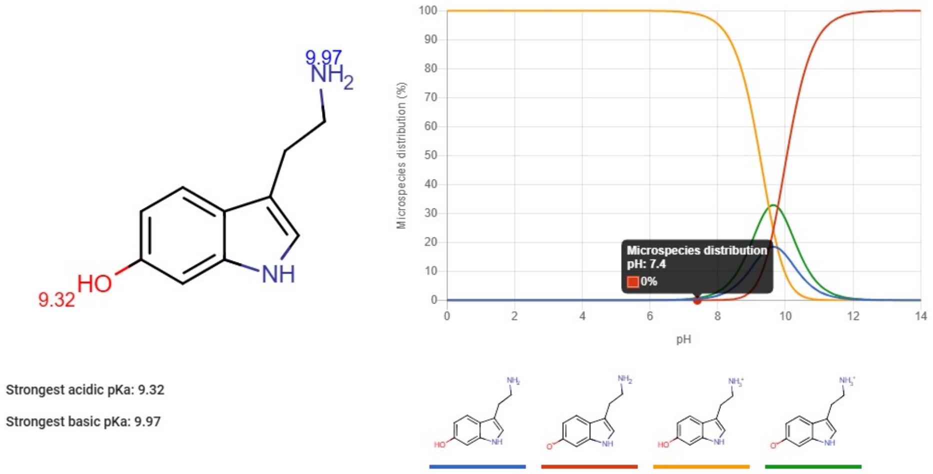
9.97
9.32
Microspecies distribution (%)
pH
Microspecies distribution
pH: 7.4
0%
Strongest acidic pKa: 9.32
Strongest basic pKa: 9.97

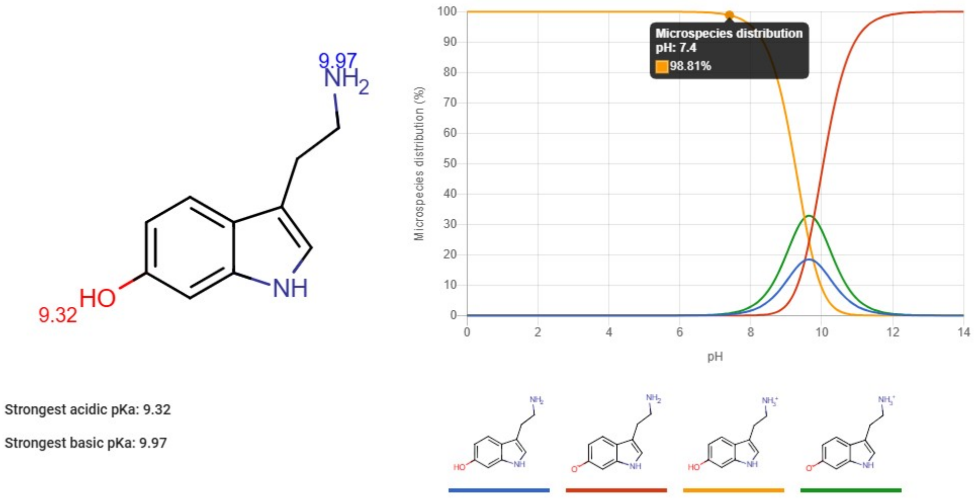

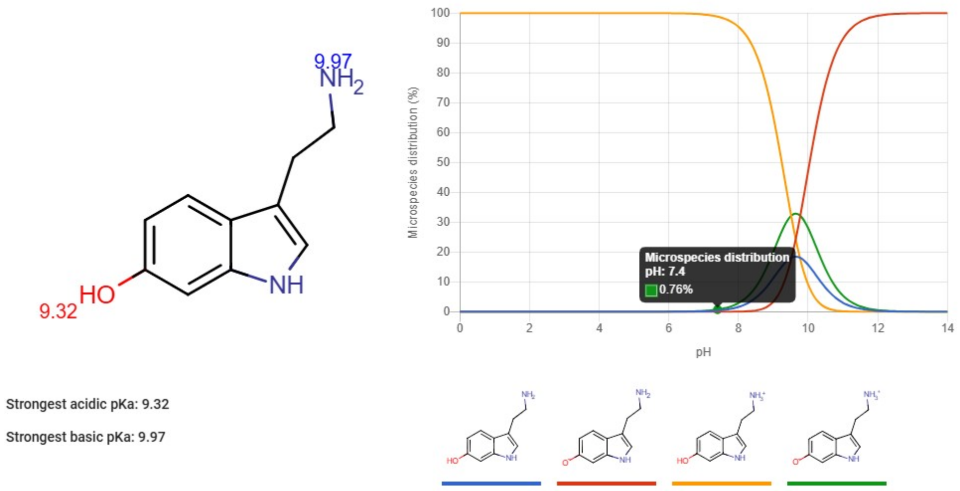

(d) 12-OH-TA

Fig. S1 pKa and microspecies contribution calculated via Chemicalize tool in Chemaxon for (a) 9-OH-TA, (b) 10-OH-TA, (c) 11-OH-TA, and (d) 12-OH-TA.

Table S1. Calculations of rate constants according to the different mechanisms.

For this study, the rate constant (k) and the activated Gibbs free energy of reactions were determined utilizing Eyringpy software. The calculation of k was performed using transition state theory, which can be expressed by Equation (1').

$$k = \sigma \times \kappa \times \frac{k_B \times T}{h} \times e^{\frac{-\Delta G^{\neq}}{RT}} \quad (1')$$

In this Equation, σ represents the total symmetry number of the reaction, κ is a

coefficient associated with tunneling correction, k_B is Boltzmann's constant, R is the gas constant, T is the temperature in Kelvin, $\Delta G^{\neq}$ denotes the Gibbs free energy of activation and h is Planck's constant.

Table S2. Distribution of the existing forms of OH-TA derivatives in water[1]

	9-OH-TA	10-OH-TA	11-OH-TA	12-OH-TA
%Cationic	97.85	98.90	98.82	98.81
%Zwitterionic	1.69	0.69	0.77	0.76
%Neutral	0.45	0.41	0.41	0.43
%Anionic	0.01	0.00	0.00	0.00

Refererence:

[1] Chemicalize was used for prediction of pK_a, March, 2025, https://chemicalize.com/, developed by ChemAxon

Table S3. A analysis of the global reactivity descriptor parameters of OH-TA derivatives.

These parameters provide insights into how molecules interact with other species, particularly in terms of electron transfer, bond formation, or bond breaking. When evaluating the capacity to scavenge free radicals, the parameter E_{HOMO} is particularly significant, as a higher E_{HOMO} indicates a stronger tendency of the molecule to react with free radicals.[37] Based on the data presented in Table 1, 9-OH-TA exhibits the highest E_{HOMO} values among the studied molecules, with values of −6.7 eV in the gas phase and −6.8 eV in the aqueous phase. These results suggest that 9-OH-TA possesses a superior free radical scavenging ability compared to the other compounds investigated.

The energy gap between the LUMO and HOMO ($\Delta_{L\text{-}H}$) is a critical factor in determining a molecule's chemical reactivity, including its potential to scavenge free radicals. A smaller $\Delta_{L\text{-}H}$ indicates a more polarizable molecule, which typically correlates

with enhanced reactivity.[38] According to Table 1, 9-OH-TA has the smallest Δ_{L-H} values among the studied compounds, measured at 6.4 eV in the gas phase and 6.7 eV in the aqueous phase. These findings highlight 9-OH-TA as the compound with the highest potential for reactivity with free radicals.

Chemical hardness is a key parameter for evaluating molecular reactivity. Within the framework of hard and soft acids and bases theory, hardness is associated with molecular stability: molecules with high hardness are typically less reactive, while those with low hardness are more polarizable and exhibit greater chemical reactivity.[39] The calculated hardness values for TA derivatives indicate that 9-OH-TA and 12-OH-TA are particularly reactive, making them well-suited for interactions with free radicals.

Electronegativity is a critical parameter in assessing a molecule's ability to donate electrons, a key aspect of antioxidant activity.[40] A lower electronegativity value indicates a greater propensity for electron donation. As shown in Table 1, 9-OH-TA and 12-OH-TA exhibit the lowest electronegativity values, with 3.4 eV in the gas phase and 3.5 eV in the aqueous phase, respectively.

Table S4 Optimized structures of 9-OH-TA forms in gas phase using M06-2X/6-311++G(d,p)

**Stable Neutral form**	**$C_{10}H_{12}N_2O$**		
0 1			
N	1.18332700	2.20933100	0.05034200
N	-4.06882400	-0.50260100	0.34079500
C	-0.64034600	0.94864000	-0.34765500
C	0.50196400	0.09056500	-0.15554800
C	-2.05713500	0.53047200	-0.59284500
C	1.62413700	0.91061600	0.08923500

C	-0.17324700	2.22396800	-0.21273200
C	-2.71849300	-0.03736800	0.66374200
C	0.69015400	-1.30058000	-0.18516700
C	2.90892300	0.39809200	0.31109900
C	1.95151500	-1.82314800	0.03380100
C	3.04759100	-0.97435000	0.27956300
H	-2.64603900	1.38011600	-0.94722400
H	-2.08282000	-0.23346600	-1.37599700
H	-0.70927900	3.15761400	-0.28797300
H	1.76140700	3.02203800	0.17722200
H	-2.79866400	0.75708700	1.41079100
H	-2.07166100	-0.81986900	1.07970500
H	3.75481200	1.04797400	0.49716900
H	-4.54920900	-0.81881500	1.17548800
H	-4.01621000	-1.29838900	-0.28725300
H	2.09820000	-2.89821100	0.01260600
H	4.02212800	-1.41720000	0.44564000
O	-0.40419100	-2.07811400	-0.43636700
H	-0.14103000	-3.00250800	-0.44816000

**Cation**	**$[C_{10}H_{12}N_2O]^{\bullet+}$**

1 2

N	1.06579000	2.24554000	0.03312700
N	-4.09769700	-0.46715800	0.35983900
C	-0.65871600	0.84649200	-0.38120100

C	0.50154700	0.07398000	-0.16223500
C	-2.05041200	0.40081000	-0.63265500
C	1.58723900	0.95081800	0.09285400
C	-0.23986800	2.19560500	-0.24132100
C	-2.73092700	-0.09888700	0.66675800
C	0.75258600	-1.31809800	-0.18349400
C	2.86700500	0.52319400	0.32889800
C	2.06134100	-1.77502100	0.05716500
C	3.07985900	-0.87711800	0.30664100
H	-2.64914700	1.21775300	-1.03773700
H	-2.05049500	-0.41725500	-1.35753200
H	-0.83622000	3.09203600	-0.33592600
H	1.59984800	3.09427200	0.16844300
H	-2.74619700	0.71915300	1.39076300
H	-2.12144800	-0.90620600	1.09346500
H	3.68540100	1.20529700	0.52040000
H	-4.67796200	-0.49832000	1.18858300
H	-4.15586500	-1.36833700	-0.09974000
H	2.26564600	-2.83967600	0.04484200
H	4.07845600	-1.25411900	0.48948100
O	-0.27234900	-2.12167100	-0.43418800
H	-0.00779800	-3.05054900	-0.45076900

**Radical Form (O24-H)**	$C_{10}H_{11}N_2O^{\bullet}$
0 2	

N	1.18458700	2.19850200	0.07025100
N	-4.03506800	-0.58741600	0.30206700
C	-0.64128000	0.94386700	-0.33153500
C	0.47529600	0.08906000	-0.15272800
C	-2.05470600	0.52836700	-0.59009300
C	1.60679400	0.88762100	0.09323000
C	-0.15966800	2.23141900	-0.18452200
C	-2.71001400	-0.07641800	0.65388700
C	0.60536300	-1.36034600	-0.21027100
C	2.88210900	0.36161400	0.29849600
C	1.95611900	-1.87038900	0.00782800
C	3.02493800	-1.04204900	0.24993900
H	-2.65057800	1.37764800	-0.93265700
H	-2.06141700	-0.22701800	-1.38131600
H	-0.68838500	3.17003300	-0.25067900
H	1.77171400	3.00463300	0.20716400
H	-2.83104600	0.70521200	1.40925600
H	-2.04072400	-0.84163900	1.06452000
H	3.73676400	1.00034000	0.48561000
H	-4.52490400	-0.90614800	1.13039600
H	-3.93350700	-1.39583100	-0.30412100
H	2.07416700	-2.94652100	-0.02997400
H	4.00836800	-1.46899300	0.40693800
O	-0.35209800	-2.11322500	-0.42709400

Anion (O24-H)	$C_{10}H_{11}N_2O^-$

-1 1

N	1.47268800	2.09633000	0.08500700
N	-3.56401100	-0.85311200	0.16647700
C	-0.54065600	1.16547600	-0.26307500
C	0.44239200	0.11892400	-0.13641300
C	-2.00238200	0.93630900	-0.49243800
C	1.68865000	0.73156800	0.08003100
C	0.12008000	2.35175600	-0.12092400
C	-2.68037600	0.20093600	0.68196800
C	0.29870800	-1.32005200	-0.20909100
C	2.88445400	0.01122700	0.24440200
C	1.53544600	-2.02275900	-0.03612400
C	2.75303000	-1.37513600	0.17768900
H	-2.50860300	1.88501800	-0.70004100
H	-2.12040900	0.30284100	-1.37506100
H	-0.25458300	3.36401000	-0.15247100
H	2.18403400	2.79074100	0.22345200
H	-3.27415300	0.90604700	1.27459000
H	-1.89451500	-0.20594100	1.32795000
H	3.83897600	0.49640800	0.40978300
H	-3.99404000	-1.33598400	0.94998300
H	-2.92294800	-1.52824000	-0.25766300
H	1.49470600	-3.10615200	-0.08050200

H	3.64590700	-1.98486700	0.29783900
O	-0.81889800	-1.88448700	-0.40430000

**Radical Form (N1-H)**	**$C_{10}H_{11}N_2O^{\bullet}$**

0 2

N	1.13888500	2.32394400	0.05470600
N	-4.05981100	-0.49814100	0.35135000
C	-0.63869400	0.91041300	-0.36586500
C	0.50711400	0.09411700	-0.16280700
C	-2.04143700	0.48988200	-0.61306700
C	1.58257100	0.99012200	0.08950900
C	-0.13990200	2.25743600	-0.20843200
C	-2.69876800	-0.06622600	0.66063000
C	0.74222900	-1.28189300	-0.18586900
C	2.86253400	0.53634800	0.32117400
C	2.03990400	-1.74859400	0.04910400
C	3.07424100	-0.85155800	0.29755800
H	-2.63838100	1.32910600	-0.97645700
H	-2.05796700	-0.29603500	-1.37563200
H	-0.74626700	3.15203700	-0.29747700
H	-2.75227600	0.73017700	1.40706400
H	-2.06026300	-0.86082300	1.06584900
H	3.67116300	1.23014500	0.51121700
H	-4.55837400	-0.75140300	1.19647500

H	-4.04019400	-1.32162000	-0.24125700
H	2.23666200	-2.81567700	0.03546000
H	4.06941500	-1.24113100	0.47564800
O	-0.30211100	-2.11141900	-0.43633000
H	0.00109000	-3.02433300	-0.44424400

**Anion (N1-H)**	**$C_{10}H_{11}N_2O^-$**

-1 1

N	1.22492700	2.29632500	0.06352100
N	-4.07104100	-0.52072200	0.33570900
C	-0.62934400	0.96554500	-0.34003000
C	0.49704000	0.11540600	-0.15882000
C	-2.05313700	0.56785800	-0.58167900
C	1.60982300	0.99613100	0.08593900
C	-0.10662500	2.25048800	-0.18913100
C	-2.71683400	-0.03187900	0.65776200
C	0.72012000	-1.26769400	-0.18273500
C	2.89999600	0.46446800	0.30514600
C	1.98681600	-1.77843100	0.03476000
C	3.07552100	-0.90349700	0.27867100
H	-2.63860500	1.43429200	-0.90860800
H	-2.10737700	-0.17968000	-1.38439300
H	-0.67927600	3.17149800	-0.26521900
H	-2.80361400	0.74292100	1.42491600

H	-2.06097800	-0.81897400	1.04719800
H	3.73157000	1.13665300	0.48919900
H	-4.47220600	-0.98984100	1.14127000
H	-3.99082700	-1.22558800	-0.39187500
H	2.14618500	-2.85429600	0.01495600
H	4.05987700	-1.32948300	0.44494600
O	-0.36482400	-2.09804600	-0.43047900
H	-0.04361200	-3.00272500	-0.42246400

**Radical Form (N2-H)**	**$C_{10}H_{11}N_2O^{\bullet}$**

0 2

N	1.08352500	2.22179400	0.06254500
N	-4.11426100	-0.55804600	0.35522300
C	-0.70579200	0.91762200	-0.35250600
C	0.45672700	0.08740000	-0.15649700
C	-2.11261800	0.47321200	-0.61138400
C	1.55655400	0.93430900	0.09769200
C	-0.27100500	2.20360400	-0.20827400
C	-2.75660300	-0.16089500	0.62135100
C	0.68048500	-1.29834200	-0.19204800
C	2.85305200	0.45322600	0.32190000
C	1.95361600	-1.79027600	0.02881800
C	3.02670700	-0.91500300	0.28285700
H	-2.71851200	1.32894300	-0.92075200

H	-2.13956100	-0.25695600	-1.42351400
H	-0.82947900	3.12399600	-0.28419700
H	1.64104400	3.04833200	0.19251000
H	-2.72443000	0.54647800	1.46613700
H	-2.16404400	-1.03286900	0.93825700
H	3.68151700	1.12332500	0.51440300
H	-4.48383300	-0.95846600	1.22303200
H	2.12725200	-2.86111800	0.00298100
H	4.01173300	-1.33364300	0.44977900
O	-0.39526500	-2.09845500	-0.44799300
H	-0.11116000	-3.01576200	-0.49052400

**Anion (N2-H)**	**$C_{10}H_{11}N_2O^-$**

-1 1

N	0.92744300	2.23997200	0.00753900
N	-4.18829000	-0.56323500	0.37613700
C	-0.75804200	0.78158400	-0.41750300
C	0.47529600	0.06129200	-0.19572800
C	-2.13649800	0.23434400	-0.62763000
C	1.50134100	0.99838100	0.07079600
C	-0.42731800	2.09866100	-0.27548000
C	-2.87374000	-0.11168600	0.68732300
C	0.81851200	-1.30124500	-0.19331600
C	2.82622700	0.63168900	0.34605500

C	2.12409900	-1.68135600	0.08329500
C	3.11519900	-0.71913700	0.35207800
H	-2.75608000	0.96930300	-1.15184000
H	-2.11085500	-0.66992300	-1.23809300
H	-1.06674300	2.96511600	-0.34627300
H	1.39927000	3.10603500	0.19623400
H	-2.79446300	0.82824800	1.30439600
H	-2.21121300	-0.83757400	1.22217700
H	3.58837200	1.37452100	0.55124600
H	-4.58118000	-0.96734400	1.22824500
H	2.38142100	-2.73628300	0.08553400
H	4.12492700	-1.05169700	0.56496600
O	-0.16131100	-2.19833500	-0.47438600
H	0.15250800	-3.08604300	-0.28656800

**Inter1-O24-HAT**	**$C_{10}H_{12}N_2O$ + HOO$^{\bullet}$**

0 2

N	-0.50038900	-2.80947600	0.58812400
N	3.98571100	1.00810300	0.09454200
C	1.05811400	-1.37080900	-0.17273700
C	-0.24908500	-0.78301200	-0.31720000
C	2.37311500	-0.73283000	-0.49692600
C	-1.19884700	-1.70743800	0.17076500
C	0.85048900	-2.60170900	0.37873700

C	2.74773300	0.35181000	0.51536400
C	-0.70778200	0.44564500	-0.82251300
C	-2.57563500	-1.44457900	0.19688100
C	-2.06964200	0.71990600	-0.80514800
C	-2.98771000	-0.22312300	-0.29400200
H	3.16506400	-1.48535200	-0.51733400
H	2.32572600	-0.28077400	-1.49200100
H	1.57055900	-3.36041100	0.64414100
H	-0.90827800	-3.64251000	0.97683200
H	2.92039000	-0.11679000	1.48817400
H	1.90022900	1.04101800	0.62599100
H	-3.28411900	-2.16644400	0.58329000
H	4.29216600	1.67700600	0.79176200
H	3.82347800	1.52984900	-0.76105100
H	-2.42807000	1.65544700	-1.22161400
H	-4.04253800	0.02325700	-0.30119700
O	0.21478200	1.31555700	-1.30980000
H	-0.16491500	2.20284000	-1.31448000
O	-0.91197900	3.33811500	0.43431600
O	-1.23664800	2.58125900	1.44378600
H	-1.54068200	1.72986100	1.05308100

**TS-O24-HAT**	**$C_{10}H_{12}N_2O$ + HOO$^{\bullet}$**
0 2	

N	-1.63042100	-2.34496200	0.52243100
N	3.92803000	-0.41645400	-0.04263500
C	0.33884700	-1.56462000	-0.25235100
C	-0.63623600	-0.52265800	-0.31473200
C	1.78687000	-1.45227300	-0.60943500
C	-1.85881600	-1.03444900	0.17569600
C	-0.31604500	-2.65571600	0.26393000
C	2.56347300	-0.64393400	0.43403200
C	-0.58649100	0.82144500	-0.76542100
C	-3.01856100	-0.26674900	0.26070300
C	-1.76654200	1.59386100	-0.67817700
C	-2.94806600	1.05763900	-0.17162500
H	2.23811600	-2.44245900	-0.70691700
H	1.87882000	-0.94882300	-1.57624600
H	0.06616200	-3.64418900	0.46883500
H	-2.31749100	-2.97989800	0.89257500
H	2.61434500	-1.21977100	1.36249600
H	2.01660200	0.28052800	0.65333300
H	-3.94338000	-0.67862400	0.64649700
H	4.47571500	0.05054400	0.67157700
H	3.90499100	0.21055000	-0.84134100
H	-1.72809200	2.61491400	-1.04208700
H	-3.83515700	1.67733300	-0.12374600
O	0.52132200	1.32482300	-1.26986400

H	0.92460600	2.06977500	-0.56856000
O	1.17179300	2.75248400	0.50253000
O	0.39806400	2.14137500	1.43655500
H	-0.45853400	2.59529100	1.39550600

**Inter2-O24-HAT**	**$C_{10}H_{12}N_2O + HOO^{\cdot}$**

0 2

N	-2.52013200	-1.85486200	0.02168800
N	3.33136600	-1.56566800	0.41932400
C	-0.36092100	-1.32729500	-0.35263500
C	-1.09738400	-0.13286100	-0.15047700
C	1.10700000	-1.49763500	-0.58491000
C	-2.44523300	-0.48113100	0.08004400
C	-1.27868900	-2.35878800	-0.23603000
C	1.92659100	-1.27211300	0.69006800
C	-0.72207000	1.27101300	-0.16719500
C	-3.45357400	0.44941300	0.30836300
C	-1.80330400	2.21742400	0.07803800
C	-3.09500700	1.81593300	0.30362300
H	1.31040900	-2.50784100	-0.94890300
H	1.44898700	-0.79940700	-1.35443800
H	-1.11698900	-3.42232300	-0.32468300
H	-3.35814600	-2.39995800	0.14252400
H	1.56831000	-1.96204000	1.46026000

H	1.75232700	-0.24984900	1.05367900	
H	-4.47822300	0.14418600	0.48247700	
H	3.86430400	-1.49586500	1.27938600	
H	3.69642300	-0.83785700	-0.18994700	
H	-1.52976200	3.26528800	0.07069900	
H	-3.86468200	2.55752600	0.48029700	
O	0.43392500	1.67241400	-0.37789800	
H	2.12294000	1.35604600	-0.69481800	
O	3.09325500	1.26299100	-0.77839700	
O	3.51988200	1.53468500	0.55259400	
H	3.86451600	2.43132400	0.47265000	
**TS- C7-RAF**				**$C_{10}H_{12}N_2O$ + HOO$^•$**
0 2				
N	-1.52487600	-1.01798000	0.99937900	
N	4.29434000	-0.33366000	0.25888200	
C	0.52753500	-0.59268100	0.11845300	
C	-0.25663500	0.59184400	0.05559600	
C	1.95161600	-0.78398200	-0.27790500	
C	-1.54270600	0.29111000	0.57038800	
C	-0.32992000	-1.59520800	0.60278400	
C	2.92116100	-0.15875100	0.73090900	
C	-0.00702900	1.89367000	-0.41764400	
C	-2.57349500	1.22846500	0.60554600	

C	-1.01958000	2.83458300	-0.37809600
C	-2.28791200	2.49522700	0.12465300
H	2.17393200	-1.84901700	-0.37439300
H	2.12009600	-0.32118900	-1.25546100
H	-2.36101700	-1.57844900	1.08165300
H	2.81991300	-0.67630400	1.68875600
H	2.63667000	0.88926100	0.88766900
H	-3.55341200	0.97861700	0.99115100
H	4.95709500	0.00763100	0.94558200
H	4.44112900	0.20654500	-0.58777400
H	-0.83619800	3.83919700	-0.74433000
H	-3.06145100	3.25361200	0.13378700
O	1.23850800	2.15426500	-0.90141200
H	1.27251400	3.05667700	-1.23170600
H	-0.02806600	-2.54601800	1.01613500
O	-0.89036400	-2.47645000	-1.07871800
O	-1.93793500	-3.28506600	-0.66445900
H	-1.54733000	-4.16674100	-0.61028500

**Product-C7-RAF**	**$C_{10}H_{12}N_2OOOH$**

0 2

N	1.06796700	1.61454400	0.80493600
N	-4.01459700	-1.35480100	0.37205600
C	-0.64663400	0.30323700	-0.05001500

C	0.54412000	-0.44076100	-0.02722000
C	-2.03067000	-0.13261000	-0.38020500
C	1.60261200	0.39310300	0.44078200
C	-0.29707300	1.71052400	0.34023300
C	-2.66938600	-0.93486800	0.76232400
C	0.84212500	-1.77236700	-0.37461200
C	2.90832800	-0.05706000	0.53065200
C	2.14878500	-2.23162300	-0.28112700
C	3.16510100	-1.37891500	0.15991300
H	-2.65791200	0.73520200	-0.59749200
H	-2.01496900	-0.76344900	-1.27453700
H	1.62704500	2.45397200	0.82065700
H	-2.75351600	-0.29490200	1.64516100
H	-2.00051300	-1.76668500	1.01741800
H	3.70214200	0.58740600	0.88624300
H	-4.48352400	-1.82190900	1.13965200
H	-3.96214700	-2.02029400	-0.39240700
H	2.38001400	-3.25577500	-0.55469200
H	4.17730500	-1.75970600	0.22024900
O	-0.19226200	-2.54995100	-0.80290400
H	0.13879300	-3.42065800	-1.04077400
H	-0.94994700	2.16786400	1.09183300
O	-0.42012000	2.49701200	-0.84751000
O	-0.15343100	3.84825800	-0.43185900

H	0.14628400	4.22621300	-1.26643600	
**TS-C9-RAF**				**$C_{10}H_{12}N_2O$ + HOO$^{\bullet}$**
0 2				
N	-0.56635700	2.68055600	0.43997300	
N	4.17536000	-0.73569900	-0.48413400	
C	0.95641200	1.02114200	0.44906500	
C	-0.27442800	0.52592900	-0.06687400	
C	2.23756900	0.26639700	0.61779200	
C	-1.21349900	1.57298000	-0.05396700	
C	0.73129200	2.34697100	0.74062000	
C	2.97498600	0.07193600	-0.70812900	
C	-0.70979000	-0.78121600	-0.44547400	
C	-2.53427800	1.42310400	-0.48719600	
C	-2.02850100	-0.89665300	-0.98762100	
C	-2.91670100	0.16555600	-0.96071300	
H	2.89667900	0.78846400	1.31569600	
H	2.01421300	-0.71500600	1.04655600	
H	1.40917100	3.07888700	1.15235500	
H	-0.98208600	3.58672000	0.57425900	
H	3.28650800	1.04916700	-1.08804700	
H	2.28019000	-0.36407900	-1.43586500	
H	-3.23244000	2.25101100	-0.47157900	
H	4.71304100	-0.82213300	-1.33911600	

H	3.90816600	-1.67617300	-0.21051500
H	-2.32812700	-1.85916500	-1.38713800
H	-3.92376000	0.02421900	-1.33341100
O	0.24100800	-1.70148700	-0.76277300
H	-0.06850500	-2.56354400	-0.44310500
O	-1.10268400	-1.44069300	1.33891400
O	-1.19242500	-2.81939400	1.18956600
H	-2.14163800	-2.99665700	1.21837000

**Product-C9-RAF**	**$C_{10}H_{12}N_2OOOH$**

0 2

N	-0.61634300	2.70437300	0.28423300
N	4.16586900	-0.57521200	-0.47559400
C	0.87561400	1.03793400	0.46930900
C	-0.37710200	0.50155800	0.09820900
C	2.16818100	0.29952000	0.63662700
C	-1.29558800	1.54618600	-0.01449600
C	0.68722400	2.40540400	0.57554700
C	2.94328700	0.20547500	-0.67957700
C	-0.78744400	-0.91003600	-0.13787100
C	-2.63743900	1.37611000	-0.39265600
C	-2.22701800	-1.01970000	-0.57097800
C	-3.05982500	0.05162200	-0.67226600
H	2.79955700	0.78657900	1.38381000

H	1.95424900	-0.71063300	0.99751400
H	1.39104400	3.17802400	0.84303700
H	-1.01901900	3.62655600	0.29223700
H	3.23201400	1.21267500	-0.99318500
H	2.27688300	-0.20491200	-1.44725600
H	-3.31954300	2.21189800	-0.47539900
H	4.73231200	-0.57808800	-1.31632300
H	3.92732600	-1.54270700	-0.28215800
H	-2.58900400	-2.02372300	-0.76216700
H	-4.08828900	-0.11096500	-0.97298800
O	0.08857000	-1.51021000	-1.06148200
H	-0.30243600	-2.34778600	-1.33273800
O	-0.61652700	-1.55437800	1.14403300
O	-0.91406000	-2.93942500	0.97023500
H	-0.04498500	-3.33336900	1.11176400
**TS-C10-RAF**		**$C_{10}H_{12}N_2O$ + $HOO^{\cdot}$**	
0 2			
N	-0.64412500	-2.01605100	0.46190300
N	4.81057000	0.19952100	-0.07128400
C	1.20937600	-0.95451700	-0.24097800
C	0.18022200	0.00961800	0.04878800
C	2.61172100	-0.67864400	-0.68624000
C	-0.95196400	-0.69073800	0.48629500

C	0.65436500	-2.17647600	0.02022600
C	3.47472000	-0.10866400	0.44067400
C	0.09800000	1.41264100	-0.04886100
C	-2.20516000	-0.07605700	0.77803500
C	-1.08562800	2.05985800	0.30972600
C	-2.18854300	1.34286000	0.75811300
H	3.07717300	-1.59512900	-1.05659900
H	2.59830200	0.03757000	-1.51381200
H	1.08875400	-3.15951300	-0.07308000
H	-1.30770300	-2.75710200	0.61910900
H	3.57387000	-0.86346700	1.22543600
H	2.95953300	0.75653700	0.87751800
H	5.42131800	0.50722000	0.67674500
H	4.75776200	0.96012700	-0.74129200
H	-1.13161000	3.14333400	0.25921400
H	-3.08074800	1.87508600	1.06412300
O	1.19349200	2.07936200	-0.48930700
H	1.00468400	3.02154200	-0.53360400
H	-2.92933700	-0.58533300	1.40152000
O	-3.15385500	-0.61845100	-0.77034300
O	-4.47194500	-0.21850300	-0.61572700
H	-4.52131700	0.59629300	-1.13126200

**Product-C10-RAF**	**$C_{10}H_{12}N_2OOOH$**

0 2

N	-0.67626800	-2.00843400	0.23937900
N	4.81226800	0.16085500	0.17772000
C	1.22653700	-0.95577000	-0.30183200
C	0.18756500	0.01117000	-0.07967100
C	2.66241700	-0.68399600	-0.62881800
C	-0.96820200	-0.68018200	0.25056000
C	0.64683600	-2.18214100	-0.10158300
C	3.43898900	-0.15146800	0.57652100
C	0.14147900	1.43395500	-0.14495800
C	-2.29494700	-0.08213800	0.53081600
C	-1.06137100	2.09054200	0.15288800
C	-2.20681500	1.41294000	0.49666000
H	3.14790000	-1.59594200	-0.98464100
H	2.72132600	0.05290000	-1.43590200
H	1.07679800	-3.16818500	-0.17805800
H	-1.35774100	-2.74243700	0.34813600
H	3.47308800	-0.92777100	1.34570100
H	2.89630500	0.70560800	0.99541000
H	5.36352000	0.44844200	0.97819500
H	4.81364100	0.93751200	-0.47583000
H	-1.07889900	3.17714800	0.12773700
H	-3.09954500	1.96318100	0.76489100

O	1.27987500	2.09004000	-0.48941400
H	1.11177700	3.03658500	-0.51995100
H	-2.71803200	-0.43381400	1.48156300
O	-3.17769800	-0.61770700	-0.49601400
O	-4.50659100	-0.25021200	-0.14733200
H	-4.70174800	0.42537700	-0.80836000

**TS-C11-RAF**	**$C_{10}H_{12}N_2O + HOO^{\cdot}$**

0 2

N	-0.43804700	2.75236700	-0.22954500
N	-4.09288900	-1.88765800	0.27291000
C	-1.53050700	0.79632900	-0.46212200
C	-0.18699000	0.54679500	0.00387200
C	-2.59470000	-0.22523000	-0.71107900
C	0.46382700	1.79431600	0.13493500
C	-1.63446300	2.15056600	-0.58511300
C	-3.11775100	-0.84302200	0.58720000
C	0.53713900	-0.60765200	0.28608100
C	1.80645100	1.92289800	0.55304400
C	1.90915600	-0.52409500	0.67386700
C	2.49304600	0.77860800	0.85085200
H	-3.43157700	0.22262000	-1.25204100
H	-2.18889100	-1.02521200	-1.33840100
H	-2.47439500	2.74638100	-0.90682300

H	-0.25607400	3.74162500	-0.25376700
H	-3.62143000	-0.06708600	1.17011000
H	-2.26585100	-1.20154200	1.17888000
H	2.26566500	2.89847900	0.65959700
H	-4.51307900	-2.25272700	1.11999500
H	-3.62773100	-2.66638100	-0.18289800
H	2.30175700	-1.33267300	1.28312900
H	3.50990300	0.83244200	1.21997400
O	-0.01738900	-1.82327900	0.09909600
H	0.70453700	-2.44280300	-0.07811000
O	2.60807800	-1.33964000	-0.83265600
O	3.90837400	-1.73681600	-0.51606300
H	4.44997000	-1.04530000	-0.91543200

**Product-C11-RAF**	**$C_{10}H_{12}N_2OOOH$**

0 2

N	-0.50895900	2.77256000	-0.10829100
N	-4.04269100	-1.96479300	0.30180500
C	-1.57865800	0.81519900	-0.41483500
C	-0.19928300	0.55519400	-0.06844700
C	-2.64284700	-0.20626600	-0.66367200
C	0.43276600	1.80401100	0.11592100
C	-1.71422500	2.17438500	-0.42424900
C	-3.07840000	-0.91120000	0.62246100

C	0.54702000	-0.60435400	0.07001100
C	1.80801000	1.92776400	0.45465900
C	1.99176700	-0.56918500	0.43192600
C	2.54894100	0.80699300	0.61758100
H	-3.51686900	0.25892200	-1.12585300
H	-2.26603700	-0.96025800	-1.36233500
H	-2.58640200	2.77271100	-0.63738100
H	-0.34768100	3.76421700	-0.06075800
H	-3.56857200	-0.18383500	1.27535300
H	-2.18606800	-1.27775200	1.14520300
H	2.25193000	2.90753700	0.59443600
H	-4.40354800	-2.38992600	1.14830700
H	-3.58200900	-2.70214300	-0.22233900
H	2.18351900	-1.19983400	1.31449700
H	3.58901500	0.87171000	0.91359800
O	-0.01850100	-1.82761200	-0.09273200
H	0.68362200	-2.45469000	-0.30649700
O	2.64261300	-1.29528600	-0.66303800
O	3.92739800	-1.70872800	-0.20982800
H	4.50803100	-1.16326000	-0.75416900

**TS-C12-RAF**	**$C_{10}H_{12}N_2O + HOO^{\bullet}$**

0 2

N	0.22894900	2.38362700	0.21585700

N	-4.74624300	-0.78635800	-0.06316100
C	-1.37548200	0.90873200	-0.35942200
C	-0.20629200	0.19469100	0.03529500
C	-2.69928200	0.34509300	-0.77567000
C	0.78925300	1.13706400	0.38717700
C	-1.06198500	2.24230600	-0.23044600
C	-3.49647200	-0.19334100	0.41396300
C	0.11246500	-1.18818500	0.13666500
C	2.05673700	0.77788500	0.81945200
C	1.35219700	-1.57295900	0.55557200
C	2.38624100	-0.60308400	0.80245400
H	-3.29411900	1.11080700	-1.27952100
H	-2.54132400	-0.46868800	-1.48911300
H	-1.67629200	3.10663200	-0.43160400
H	0.69833600	3.25835800	0.37765300
H	-3.74388300	0.63827400	1.07957300
H	-2.86286400	-0.89097300	0.97572300
H	2.79988900	1.51288600	1.10329800
H	-5.32624200	-1.07805000	0.71519800
H	-4.54672400	-1.61827200	-0.60949200
H	1.60227700	-2.62552900	0.62976000
H	3.24143200	-0.92118100	1.38579800
O	-0.88289500	-2.06204200	-0.18337500
H	-0.54146200	-2.96027700	-0.14332900

O	3.37014000	-0.78036600	-0.78641000
O	4.54990700	-0.06885900	-0.65176400
H	4.37053900	0.75605800	-1.12066000
**Product-C12-RAF**		**$C_{10}H_{12}N_2OOOH$**	
0 2			
N	0.13812400	2.42430600	0.10071700
N	-4.75108600	-0.87925000	0.17437000
C	-1.45160900	0.89487300	-0.36616600
C	-0.24376300	0.22288800	-0.07262500
C	-2.78862900	0.29932800	-0.68557900
C	0.75566000	1.19240100	0.21645400
C	-1.17511800	2.24678600	-0.25103700
C	-3.47864600	-0.26610500	0.55737000
C	0.13415700	-1.16487100	-0.02837800
C	2.05333700	0.89226400	0.54039700
C	1.39576500	-1.52994800	0.27356700
C	2.48868600	-0.53905900	0.52434600
H	-3.43934700	1.05296400	-1.13571800
H	-2.66499500	-0.50604900	-1.41488900
H	-1.83169700	3.08961200	-0.40214100
H	0.59842900	3.31141900	0.21452800
H	-3.68828100	0.55367200	1.25007300
H	-2.78879200	-0.95634500	1.05854600

H	2.79922100	1.64930500	0.74376300
H	-5.26092700	-1.19142400	0.99288800
H	-4.58009900	-1.70055300	-0.39709500
H	1.67100900	-2.57883000	0.31016500
H	3.02246900	-0.78203900	1.45361400
O	-0.87024200	-2.04881200	-0.29198200
H	-0.51247600	-2.94166200	-0.29444700
O	3.45079100	-0.76864800	-0.54268900
O	4.61518500	-0.00771600	-0.22646100
H	5.26132000	-0.70539500	-0.06593100

Table S5 Optimized structures of 9-OH-TA forms in water using M06-2X/6-311++G(d,p)

**Stable Cationic form**	**$[C_{10}H_{13}N_2O]^+$**		
1 1			
N	1.21398600	2.21405800	0.05013400
N	-4.03305200	-0.52399700	0.35851200
C	-0.59610000	0.93972800	-0.34717600
C	0.54898300	0.08471100	-0.15763000
C	-2.01479000	0.53123000	-0.60084800
C	1.66354100	0.91900400	0.08778900
C	-0.13656300	2.22094300	-0.20928400
C	-2.64679700	-0.04613500	0.65752500
C	0.75265700	-1.30652000	-0.18541400
C	2.95419200	0.41934700	0.31156900

C	2.01881400	-1.81657600	0.03653600
C	3.10620600	-0.95332400	0.28265700
H	-2.59188000	1.40131100	-0.92538900
H	-2.06114100	-0.21771500	-1.39674800
H	-0.67802100	3.15220900	-0.28141800
H	1.78785100	3.03424500	0.18279900
H	-2.72967000	0.70268300	1.44334500
H	-2.09024100	-0.90392300	1.03011900
H	3.78901700	1.08395200	0.49654100
H	-4.47788900	-0.92284400	1.18915200
H	-4.01672200	-1.24442300	-0.36899300
H	2.16734800	-2.89096300	0.01336100
H	4.08544800	-1.38645300	0.44998300
O	-0.33376200	-2.10123700	-0.44074400
H	-0.06159000	-3.02806700	-0.44427400
H	-4.61981200	0.24500100	0.02260100

**Cation radical**	**$[C_{10}H_{13}N_2O]^{\bullet+}$**

2 2

N	1.18330400	2.23253200	0.04691400
N	-4.02524300	-0.52164600	0.34962200
C	-0.58897400	0.90346600	-0.34543000
C	0.53817200	0.08393900	-0.15673000
C	-2.00064500	0.50589000	-0.60823000
C	1.65648900	0.92358800	0.08896100

C	-0.12344500	2.23294700	-0.20512700
C	-2.64437200	-0.04678200	0.66035000
C	0.74255500	-1.31754900	-0.18475700
C	2.92172100	0.45008500	0.30775900
C	2.04046500	-1.82130500	0.04087300
C	3.08887100	-0.95962200	0.27954300
H	-2.56574000	1.37203600	-0.95845300
H	-2.02407500	-0.26396900	-1.38506800
H	-0.68711200	3.15110900	-0.28262100
H	1.75347300	3.06108700	0.18436300
H	-2.73051400	0.71761800	1.43002100
H	-2.09210700	-0.89892800	1.05162000
H	3.75917000	1.11051800	0.49209100
H	-4.47827100	-0.90589300	1.18321300
H	-4.00474700	-1.25454500	-0.36559300
H	2.19220100	-2.89423300	0.01848000
H	4.07759400	-1.36747400	0.44899500
O	-0.30363700	-2.09311800	-0.42707400
H	-0.05525300	-3.03087900	-0.43699300
H	-4.60697300	0.24434500	-0.00248800

**Radical Form (O24-H)**	**$[C_{10}H_{12}N_2O]^{+\bullet}$**

1 2

N	1.18860400	2.20262100	0.06565100
N	-4.02496900	-0.58305600	0.33981100

C	-0.60274300	0.90958500	-0.33870800
C	0.52520400	0.07308500	-0.16001700
C	-2.01796600	0.50224800	-0.60285300
C	1.63921700	0.90291700	0.09030600
C	-0.14390300	2.21114800	-0.18815800
C	-2.64085000	-0.10716300	0.64527400
C	0.70076100	-1.36477300	-0.21151300
C	2.92291900	0.41438700	0.30470100
C	2.05400300	-1.84255200	0.01978800
C	3.10061400	-0.98817800	0.26436000
H	-2.59984100	1.37456400	-0.91071500
H	-2.05044800	-0.23286100	-1.41224800
H	-0.69238500	3.13884600	-0.25234800
H	1.76292100	3.02336300	0.20954600
H	-2.72317600	0.62265000	1.44861500
H	-2.07718300	-0.97074900	0.99316600
H	3.75568600	1.08035100	0.49363300
H	-4.46506200	-0.99968300	1.16428500
H	-4.00839200	-1.28916900	-0.40161900
H	2.20642400	-2.91535600	-0.01122200
H	4.09304400	-1.39030600	0.42918900
O	-0.24436200	-2.15414200	-0.44362200
H	-4.61567500	0.19031100	0.02137500

**Anion (O24-H)**	**[$C_{10}H_{12}N_2O$]**

2 1			
N	1.15294900	2.23495200	0.06496600
N	-4.03283100	-0.57429600	0.32256500
C	-0.59630000	0.85927700	-0.33761800
C	0.50940200	0.07587500	-0.15860500
C	-2.00480400	0.45872100	-0.61616400
C	1.63501200	0.92124900	0.09322300
C	-0.13345600	2.22174200	-0.18181700
C	-2.65426700	-0.10271100	0.64491400
C	0.70657800	-1.39142200	-0.20169800
C	2.88549800	0.45956500	0.29285400
C	2.10020400	-1.85322400	0.02044200
C	3.09998900	-0.98552900	0.25371900
H	-2.56962400	1.32269300	-0.97119000
H	-2.01018600	-0.30959000	-1.39463600
H	-0.71237900	3.13266700	-0.25113000
H	1.71725700	3.06940600	0.21165000
H	-2.74402700	0.65409900	1.42148500
H	-2.10263500	-0.95850600	1.02911700
H	3.71969000	1.12700900	0.47542500
H	-4.48824600	-0.96886600	1.15011000
H	-4.00897300	-1.29891600	-0.40108400
H	2.26516000	-2.92343600	-0.00987700
H	4.10879300	-1.34305800	0.41683900

O	-0.21108500	-2.16308300	-0.40281100	
H	-4.61411900	0.19532000	-0.02242900	
**Radical Form (N1-H)**				**$[C_{10}H_{12}N_2O]^{+\bullet}$**
1 2				
N	1.18798500	2.32830100	0.05764100	
N	-4.01502500	-0.52964800	0.35527700	
C	-0.58709200	0.91392500	-0.34981900	
C	0.55342500	0.09913200	-0.15751000	
C	-1.99490000	0.50785200	-0.60955600	
C	1.63457100	0.99327700	0.08981200	
C	-0.09781800	2.25892400	-0.19737200	
C	-2.62496600	-0.07049200	0.65439100	
C	0.78743700	-1.28296800	-0.18425900	
C	2.91236500	0.53649300	0.31354200	
C	2.08897800	-1.75283800	0.04617200	
C	3.12134600	-0.85827000	0.28890200	
H	-2.57417800	1.37325200	-0.94036100	
H	-2.02323700	-0.25246100	-1.39668600	
H	-0.71219700	3.14727000	-0.28290300	
H	-2.69566100	0.67578400	1.44336700	
H	-2.07319400	-0.93515200	1.01745900	
H	3.73228900	1.21937400	0.49993700	
H	-4.45876300	-0.92953100	1.18635000	

H	-4.01079600	-1.24548600	-0.37707700
H	2.27240400	-2.82151600	0.02682100
H	4.11833100	-1.24584100	0.46167400
O	-0.24982500	-2.10828300	-0.43495500
H	0.04832300	-3.02865200	-0.44428900
H	-4.59551900	0.24844800	0.02910000

**Anion (N1-H)**	**[$C_{10}H_{12}N_2O$]**

2 1

N	1.16656300	2.35600900	0.05793700
N	-4.00722100	-0.51880700	0.33651700
C	-0.57290700	0.86758300	-0.34413600
C	0.54352100	0.10301300	-0.14414100
C	-1.96831200	0.46666900	-0.62331400
C	1.63544100	1.00922000	0.09686900
C	-0.08926600	2.27678800	-0.19407600
C	-2.62683200	-0.05755900	0.65753700
C	0.77356000	-1.30552700	-0.17521000
C	2.88695900	0.56541000	0.30214300
C	2.10145100	-1.77458000	0.04470700
C	3.09582200	-0.87247600	0.27297100
H	-2.53003000	1.32214400	-1.00231300
H	-1.96766300	-0.33347900	-1.37105800
H	-0.73302900	3.14276500	-0.28917600

H	-2.71023700	0.72236900	1.41100500
H	-2.08504200	-0.90849000	1.06476400
H	3.72158000	1.23134300	0.47867800
H	-4.47232600	-0.88197200	1.17345000
H	-3.98965000	-1.26567600	-0.36462000
H	2.28874600	-2.84125400	0.02473100
H	4.10212500	-1.23887300	0.43958900
O	-0.23236500	-2.08759700	-0.40354900
H	0.01857000	-3.02848400	-0.42405800
H	-4.57613300	0.24871700	-0.03387000

**Radical Form (N2-H)**	**$[C_{10}H_{12}N_2O]^{+\bullet}$**

1 2

N	1.04266300	2.24944100	0.04328200
N	-4.06717600	-0.60685100	0.35689900
C	-0.66562800	0.84139400	-0.37838400
C	0.49640400	0.07249600	-0.16946400
C	-2.05650500	0.38472900	-0.63478200
C	1.57299700	0.96051800	0.09268000
C	-0.25787800	2.19242600	-0.22896700
C	-2.71268400	-0.13411800	0.65050900
C	0.76316600	-1.31636600	-0.19258100
C	2.85522100	0.54680000	0.33214100
C	2.07715300	-1.75998700	0.05459600

C	3.08434300	-0.85195200	0.30826400	
H	-2.64432300	1.21815900	-1.02753500	
H	-2.05186500	-0.41668900	-1.37741500	
H	-0.86024700	3.08510500	-0.31405000	
H	1.57403800	3.10175800	0.18959100	
H	-2.68899500	0.66426700	1.40117300	
H	-2.12786400	-0.96915900	1.03991900	
H	3.65837500	1.24507500	0.52861700	
H	-4.48031500	-0.96915800	1.21138000	
H	2.27791200	-2.82510500	0.03733800	
H	4.08687500	-1.21719000	0.49359800	
O	-0.24158700	-2.14446100	-0.45224600	
H	0.05992000	-3.06585900	-0.45951100	
H	-4.63875300	0.19070600	0.08953200	
**Anion (N2-H)**				**[$C_{10}H_{12}N_2O$]**
2 1				
N	0.96986700	2.27954100	0.03818200	
N	-4.07294300	-0.59950900	0.34766500	
C	-0.66933600	0.76550700	-0.38682800	
C	0.48714100	0.06865000	-0.16115700	
C	-2.03703800	0.29961200	-0.66288900	
C	1.55033300	0.98967000	0.09792300	
C	-0.29066900	2.19097400	-0.23230200	

C	-2.72316100	-0.13378100	0.65316500	
C	0.78852200	-1.33915200	-0.18002900	
C	2.81909300	0.62219800	0.32378800	
C	2.13996300	-1.74803800	0.06038200	
C	3.09188700	-0.81171000	0.29839800	
H	-2.61246800	1.10698900	-1.11978800	
H	-2.00458000	-0.56031300	-1.33486200	
H	-0.93502300	3.05564100	-0.32710800	
H	1.48108800	3.15124700	0.18743000	
H	-2.69964300	0.70620800	1.35474300	
H	-2.15320800	-0.95404300	1.09078300	
H	3.61614700	1.33001200	0.51102900	
H	-4.49072500	-0.96026900	1.20079200	
H	2.36836900	-2.80692100	0.04546700	
H	4.11162700	-1.12875500	0.47963600	
O	-0.17138300	-2.15418700	-0.41238600	
H	0.11127300	-3.08860600	-0.42665400	
H	-4.64066700	0.19850600	0.07399800	
**Inter1-O24-HAT**				**$[C_{10}H_{13}N_2O]^+ + HOO^{\bullet}$**
1 2				
N	-1.00762400	-2.38573000	0.54108300	
N	4.09223400	0.56581200	0.01836500	
C	0.77675800	-1.27178500	-0.25524900	

C	-0.41029000	-0.49080300	-0.49179800
C	2.19519800	-0.90638300	-0.56922600
C	-1.50680000	-1.21957600	0.02433100
C	0.35685300	-2.41226600	0.37334300
C	2.68872000	0.18485100	0.37033900
C	-0.66054100	0.76302400	-1.07836100
C	-2.82664200	-0.74639300	-0.01752700
C	-1.95662900	1.25235700	-1.11585600
C	-3.02804300	0.49278200	-0.59253600
H	2.83025900	-1.78969800	-0.45983900
H	2.28320800	-0.55555700	-1.60154500
H	0.93678900	-3.25514000	0.71823300
H	-1.55839600	-3.11550100	0.97019500
H	2.69910200	-0.15428200	1.40481700
H	2.08574000	1.08688700	0.28980900
H	-3.64494000	-1.32875300	0.38720800
H	4.44708800	1.29544600	0.64100300
H	4.14115700	0.91763400	-0.94210400
H	-2.14366400	2.21913900	-1.57108100
H	-4.02912500	0.90370900	-0.64603400
O	0.40489900	1.45099700	-1.58595100
H	0.10485700	2.29839500	-1.94021700
O	0.02214600	1.82118000	1.81398800
O	-1.24792600	1.60003000	1.99208500

H	-1.66656000	1.60664100	1.10069600	
H	4.71776200	-0.24200600	0.08697600	
**TS-O24-HAT**				**$[C_{10}H_{13}N_2O]^+ + HOO^{\bullet}$**
1 2				
N	-1.52713500	-2.43110000	0.52882100	
N	3.95354700	-0.26468200	0.02450300	
C	0.38835200	-1.53862900	-0.24223100	
C	-0.64202800	-0.55110800	-0.31403400	
C	1.83390300	-1.37721000	-0.59318600	
C	-1.83124800	-1.13885600	0.17919300	
C	-0.20493500	-2.66630900	0.27980900	
C	2.52840200	-0.48180900	0.42384200	
C	-0.68840900	0.79091700	-0.76111400	
C	-3.03820600	-0.44673000	0.26475100	
C	-1.90570300	1.49099100	-0.66940100	
C	-3.05214000	0.88016200	-0.16227500	
H	2.31906800	-2.35653200	-0.60630200	
H	1.93665000	-0.93473300	-1.58845100	
H	0.23354600	-3.63003300	0.49179000	
H	-2.18069900	-3.10441300	0.90481000	
H	2.53945800	-0.93598100	1.41303700	
H	2.06193000	0.50052500	0.48042700	
H	-3.92998500	-0.92392500	0.65288000	

H	4.43897400	0.33546400	0.69579200
H	4.00731800	0.18039400	-0.89632100
H	-1.93033400	2.52011200	-1.01081600
H	-3.97324800	1.44684500	-0.10506600
O	0.39645500	1.36364200	-1.29849300
H	0.74435700	2.11880300	-0.64899100
O	1.01615800	2.86274100	0.49286400
O	0.16719200	2.30547400	1.38650900
H	-0.67563200	2.78533800	1.29336000
H	4.45735400	-1.15476000	-0.02857700

**Inter2-O24-HAT**	**$[C_{10}H_{13}N_2O]^+ + HOO^•$**

1 2

N	1.67756700	2.30213400	0.64578400
N	-3.89857100	0.48410400	-0.12899700
C	-0.26349800	1.56423100	-0.21138300
C	0.70186600	0.53843000	-0.33533300
C	-1.70657100	1.51908800	-0.60303500
C	1.91268800	1.01717600	0.21118500
C	0.38627400	2.63052200	0.39941000
C	-2.47814900	0.59037800	0.32502900
C	0.64492700	-0.79990400	-0.87931900
C	3.07383700	0.25910800	0.27048400
C	1.87080100	-1.57210100	-0.79553800

C	3.01918900	-1.05776900	-0.24683900
H	-2.13323800	2.52377800	-0.55216600
H	-1.80386300	1.15889700	-1.63143900
H	-0.00255700	3.60145200	0.66746500
H	2.36375800	2.90779500	1.07807400
H	-2.49519600	0.96928700	1.34522900
H	-2.06679300	-0.41737000	0.31742100
H	3.98579900	0.65517200	0.69951300
H	-4.43814000	-0.12799800	0.48824700
H	-3.94761500	0.10088900	-1.07743900
H	1.84311700	-2.58121500	-1.19104800
H	3.91352400	-1.66785700	-0.20699000
O	-0.40232500	-1.27911900	-1.38671000
H	-1.06852100	-2.50772500	-0.22045600
O	-1.21897800	-2.98157600	0.62312800
O	-0.57331000	-2.13711500	1.56554300
H	0.33480900	-2.47497100	1.58110000
H	-4.34934100	1.40373200	-0.13867500

**TS- C7-RAF**	**$[C_{10}H_{13}N_2O]^+ + HOO^{\cdot}$**

1 2

N	-1.42506400	1.21695900	-1.01938200
N	4.23404300	-0.34757700	-0.27724100
C	0.54065800	0.49696500	-0.16287400

C	-0.40976600	-0.55072400	-0.05694200
C	1.97571100	0.50304700	0.24472000
C	-1.63955900	-0.07033900	-0.58171200
C	-0.15578700	1.60844300	-0.68576500
C	2.81390500	-0.30285700	-0.74023100
C	-0.35880200	-1.86781800	0.44805600
C	-2.79716400	-0.84434200	-0.61215000
C	-1.50064600	-2.64760700	0.41697200
C	-2.70041300	-2.13271800	-0.10714700
H	2.34057500	1.53273500	0.27466900
H	2.08418800	0.07159400	1.24442200
H	-2.14836200	1.84531300	-1.34064900
H	2.81302400	0.15038500	-1.72958300
H	2.46911900	-1.33236200	-0.81060200
H	-3.72522800	-0.45597300	-1.01151100
H	4.81758000	-0.88117200	-0.92673600
H	4.30146900	-0.78688000	0.64549600
H	-1.46348000	-3.65951700	0.80482000
H	-3.57672100	-2.77030600	-0.11219900
O	0.82911300	-2.30223200	0.94901300
H	0.73462700	-3.20758100	1.27445400
H	0.27251800	2.50020700	-1.11743400
O	-0.37900100	2.58936200	1.07914300
O	-1.32551000	3.56816000	0.80237900

H	-0.81316000	4.31728700	0.46340200
H	4.62535600	0.59596700	-0.20402800

**Product-C7-RAF**	**$[C_{10}H_{13}N_2O]^{+}OOH$**

1 2			
N	1.12165400	1.57629000	0.85600900
N	-4.02877800	-1.22427200	0.40482700
C	-0.59962300	0.32214000	-0.06397300
C	0.56906600	-0.45602800	-0.03388200
C	-1.99681300	-0.06757900	-0.40454300
C	1.63615800	0.34903500	0.46544900
C	-0.21987100	1.71591200	0.33695300
C	-2.63130100	-0.82607300	0.75756400
C	0.84239400	-1.79324900	-0.39000600
C	2.93134900	-0.12745100	0.56662700
C	2.13804500	-2.28097600	-0.28279700
C	3.16458700	-1.45161400	0.18174700
H	-2.58690100	0.82920500	-0.61327600
H	-2.01569000	-0.70128300	-1.29576900
H	1.72013100	2.39308600	0.86944500
H	-2.69055500	-0.20865500	1.65207900
H	-2.08518300	-1.74066700	0.98141000
H	3.73158600	0.49968400	0.93931000
H	-4.47589900	-1.72581600	1.17651100

H	-4.03658100	-1.83475600	-0.41754900
H	2.34221900	-3.30723800	-0.56779000
H	4.16881900	-1.85297800	0.24951000
O	-0.19969100	-2.54635000	-0.84322600
H	0.11285300	-3.43406800	-1.06262800
H	-0.89302800	2.20256000	1.04619600
O	-0.26532400	2.49633100	-0.87866400
O	-0.06266400	3.86383900	-0.53247000
H	0.87482500	4.00329900	-0.73566100
H	-4.59923900	-0.40177000	0.18840500

**TS-C9-RAF**	**$[C_{10}H_{13}N_2O]^+ + HOO^\bullet$**

1 2

N	-0.51298000	2.67635900	0.44858800
N	4.17127700	-0.79857400	-0.50334500
C	0.95197800	0.97241900	0.46355700
C	-0.28467200	0.51306300	-0.06734100
C	2.22163400	0.19838500	0.64188600
C	-1.18840200	1.59327200	-0.05786900
C	0.76306200	2.30834200	0.75827700
C	2.93750700	0.02558000	-0.69006900
C	-0.76883400	-0.77163700	-0.46646000
C	-2.50606100	1.49552100	-0.51532300
C	-2.08300400	-0.84188000	-1.02585600

C	-2.92773300	0.25442100	-1.00648800
H	2.87788300	0.72749800	1.33775300
H	2.00514000	-0.78699700	1.06432400
H	1.45974700	3.02038000	1.17441700
H	-0.90320200	3.60068700	0.57272400
H	3.25237800	0.98349400	-1.09997100
H	2.31508900	-0.49339900	-1.41629000
H	-3.16822900	2.35237900	-0.50268200
H	4.67289600	-0.92132900	-1.38632300
H	3.93716600	-1.72730700	-0.14057400
H	-2.41624600	-1.79718500	-1.41734100
H	-3.93456500	0.15233700	-1.39230100
O	0.15180600	-1.72587800	-0.74432300
H	-0.29657900	-2.56781100	-0.91124500
O	-1.26042400	-1.38317100	1.35960600
O	-1.45437400	-2.76007200	1.26075000
H	-2.40744600	-2.86344100	1.12227700
H	4.80896100	-0.35574600	0.16438500

**Product-C9-RAF**	**$[C_{10}H_{13}N_2O]^+OOH$**

1 2

N	0.55435400	2.70424700	-0.30378500
N	-4.18157700	-0.63173300	0.48563900
C	-0.87214100	0.98346600	-0.45603400

C	0.40006400	0.49371400	-0.07982700
C	-2.14429400	0.21011400	-0.63331100
C	1.27722900	1.57839000	0.00694500
C	-0.73376100	2.35754400	-0.58093600
C	-2.93425600	0.17429000	0.66723000
C	0.87669000	-0.89634500	0.16113500
C	2.62963700	1.46902900	0.37541800
C	2.31019800	-0.93873400	0.61687100
C	3.10090800	0.16945200	0.69032300
H	-2.75810000	0.67397600	-1.41013300
H	-1.91911400	-0.81228900	-0.94746000
H	-1.46624200	3.10194800	-0.85282400
H	0.92448200	3.64503300	-0.32183800
H	-3.24192200	1.17247200	0.97375200
H	-2.36766000	-0.29296200	1.47014200
H	3.27367100	2.33629200	0.43469700
H	-4.73755700	-0.65398400	1.34434600
H	-3.95685900	-1.59756900	0.22922700
H	2.69806300	-1.91724000	0.87697100
H	4.13046700	0.05647700	1.01042900
O	0.01628300	-1.55513300	1.06151600
H	0.45009800	-2.36167500	1.37286200
O	0.74585500	-1.53539600	-1.14045100
O	1.15561900	-2.89295900	-1.03780600

H	0.32182000	-3.35694100	-0.86696300
H	-4.76428600	-0.23873800	-0.25914800

TS-C10-RAF	$[C_{10}H_{13}N_2O]^+ + HOO^{\bullet}$

1 2

N	0.63063800	-2.05682500	-0.48333900
N	-4.76518600	0.30530200	0.07446400
C	-1.18050100	-0.94117200	0.23495100
C	-0.12532800	-0.00490600	-0.04719900
C	-2.57205500	-0.64281300	0.70086300
C	0.98272600	-0.74191700	-0.49599100
C	-0.66881400	-2.17950200	-0.04993500
C	-3.40363600	-0.05381300	-0.42942800
C	-0.01078700	1.39931900	0.02267600
C	2.24324600	-0.15560300	-0.80279600
C	1.18432400	2.01592000	-0.37190300
C	2.26048700	1.26610500	-0.81661000
H	-3.04329200	-1.56461400	1.05179300
H	-2.55121500	0.06351700	1.53587800
H	-1.13610200	-3.14951800	0.02335000
H	1.23323800	-2.82414500	-0.74903500
H	-3.54015900	-0.76962900	-1.23790300
H	-2.95995700	0.85890700	-0.82289200
H	-5.35092300	0.68573000	-0.67276800

H	-4.70222700	1.00701400	0.81752000
H	1.24421900	3.09855200	-0.33954600
H	3.16475000	1.77162300	-1.13305200
O	-1.07814900	2.10037600	0.46064700
H	-0.88841100	3.04915000	0.44386600
H	2.97088500	-0.70865400	-1.38276000
O	3.18329800	-0.57874200	0.85217400
O	4.51460100	-0.17804700	0.71000900
H	4.52366900	0.74066600	1.01563800
H	-5.23862200	-0.51633800	0.46163800

**Product-C10-RAF**	**$[C_{10}H_{13}N_2O]^+OOH$**

1 2

N	-0.68244000	-2.04141200	0.30011100
N	4.75358100	0.26853000	0.15089500
C	1.18359600	-0.95630500	-0.28926400
C	0.12755300	-0.00480500	-0.08273600
C	2.61245600	-0.67673500	-0.64307700
C	-1.00821000	-0.71972100	0.28039800
C	0.63733000	-2.19226700	-0.04231200
C	3.35829700	-0.09472600	0.54933000
C	0.05896800	1.41930900	-0.13595200
C	-2.34199900	-0.13629500	0.54794100
C	-1.13609900	2.05736000	0.23486100

C	-2.25571500	1.35605500	0.61197400
H	3.09990600	-1.60465900	-0.95382300
H	2.66958900	0.02779700	-1.47776000
H	1.09154300	-3.17002400	-0.08476100
H	-1.32271800	-2.79649200	0.50716200
H	3.43487600	-0.81463500	1.36226100
H	2.88651200	0.81588600	0.91339100
H	5.27668300	0.65734200	0.93922600
H	4.74516800	0.96512300	-0.59945400
H	-1.15247100	3.14343100	0.24417800
H	-3.14296200	1.89032700	0.92877100
O	1.17227300	2.09892500	-0.51672500
H	1.00702700	3.05158600	-0.48674100
H	-2.81912100	-0.56463100	1.43535000
O	-3.18192300	-0.57075200	-0.58356400
O	-4.53056300	-0.22811400	-0.29744200
H	-4.62806600	0.64968500	-0.69755400
H	5.26069200	-0.55224400	-0.19240800

**TS-C11-RAF**	**$[C_{10}H_{13}N_2O]^+ + HOO^{\bullet}$**

1 2

N	0.19314100	2.70732800	0.20818500
N	4.26474000	-1.57714200	-0.22703200
C	1.41545800	0.83872000	0.46537200

C	0.10064400	0.48648000	-0.01456600
C	2.56050300	-0.08194200	0.75249400
C	-0.62812300	1.68855900	-0.16465800
C	1.41868800	2.20026900	0.58540600
C	3.16741000	-0.61059100	-0.53938600
C	-0.53319100	-0.71502000	-0.33565300
C	-1.95684400	1.73414800	-0.65259500
C	-1.91600600	-0.71575000	-0.69534700
C	-2.56222000	0.54895300	-0.95225200
H	3.32650000	0.45653700	1.31663300
H	2.22496100	-0.92581600	1.36242800
H	2.20572600	2.85812700	0.92094700
H	-0.05900300	3.68648300	0.21000600
H	3.60616200	0.19106900	-1.13067100
H	2.43487800	-1.14601300	-1.14036300
H	-2.45512300	2.68444300	-0.80197500
H	4.68576600	-1.95244000	-1.08080300
H	3.90807000	-2.36490300	0.32165100
H	-2.27981000	-1.58972800	-1.22688000
H	-3.56983500	0.53211000	-1.34899700
O	0.14827500	-1.87394600	-0.21460100
H	-0.42576800	-2.62265800	-0.42962000
O	-2.63220200	-1.29711600	0.92746900
O	-3.98760300	-1.59028600	0.70139100

H	-4.43836300	-0.74375700	0.83360500	
H	5.00500200	-1.12694100	0.31899600	
**Product-C11-RAF**				**$[C_{10}H_{13}N_2O]^+OOH$**
1 2				
N	-0.31204400	2.75149400	-0.14372000	
N	-4.18834100	-1.71005200	0.28751000	
C	-1.46943200	0.84772400	-0.43408500	
C	-0.11358700	0.52030500	-0.05575900	
C	-2.59445400	-0.10211100	-0.70567800	
C	0.57094100	1.74263100	0.11979800	
C	-1.53437100	2.21540600	-0.47485800	
C	-3.11105700	-0.71663100	0.58815500	
C	0.57164200	-0.67197000	0.15655800	
C	1.92683700	1.81345500	0.54970600	
C	2.02599500	-0.68533800	0.46148300	
C	2.60716900	0.66211200	0.75589800	
H	-3.41085400	0.43096200	-1.20026600	
H	-2.26364700	-0.90285700	-1.37410600	
H	-2.36737300	2.85489300	-0.72417200	
H	-0.09890300	3.73843300	-0.10241700	
H	-3.54685300	0.03808900	1.24038600	
H	-2.32779900	-1.25020000	1.12315500	
H	2.38705800	2.77786200	0.73450000	

H	-4.55687800	-2.13238100	1.14347400
H	-3.83179400	-2.46236900	-0.30867600
H	2.25081000	-1.41227300	1.25201000
H	3.62900300	0.68277500	1.11601500
O	-0.08280800	-1.86196800	0.05660600
H	0.54130400	-2.59271000	0.16325300
O	2.64992400	-1.26891000	-0.75203500
O	3.96711800	-1.69612300	-0.42544900
H	4.51449300	-0.93291800	-0.66446300
H	-4.96783700	-1.26489600	-0.20550700

**TS-C12-RAF**	**$[C_{10}H_{13}N_2O]^+ + HOO^•$**

1 2

N	-0.27915700	2.37203000	-0.23040600
N	4.72098000	-0.75598400	0.01926700
C	1.32122600	0.90262600	0.35573400
C	0.16204500	0.17940500	-0.03384400
C	2.65421100	0.36572300	0.77902700
C	-0.83444700	1.12280900	-0.39578600
C	1.00059000	2.24047100	0.21552000
C	3.42252200	-0.16163600	-0.42461100
C	-0.15421900	-1.20755600	-0.13318100
C	-2.09708000	0.76219700	-0.83235300
C	-1.39336500	-1.59500500	-0.55646500

C	-2.41815100	-0.62249400	-0.81766100
H	3.23267400	1.16191800	1.25464800
H	2.52805100	-0.44038300	1.50679400
H	1.61135200	3.10903700	0.41183300
H	-0.75418400	3.24830100	-0.39838800
H	3.65961100	0.63597900	-1.12625100
H	2.87250700	-0.94735400	-0.93896600
H	-2.83737000	1.50115500	-1.11280600
H	5.25923700	-1.10859700	-0.77594300
H	4.56221800	-1.53527200	0.66459700
H	-1.62774500	-2.65010700	-0.64270400
H	-3.28375700	-0.94524700	-1.38266000
O	0.83964300	-2.08185500	0.19046300
H	0.51127000	-2.98760600	0.11303300
O	-3.41863400	-0.73744100	0.83071100
O	-4.62889900	-0.06334400	0.66933400
H	-4.43698900	0.84725700	0.93735100
H	5.29348800	-0.05952100	0.50509000

**Product-C12-RAF**	**$[C_{10}H_{13}N_2O]^+OOH$**

1 2

N	0.17924500	2.42003700	0.12320400
N	-4.72318100	-0.85358900	0.18620600
C	-1.39363000	0.88831700	-0.37260100

C	-0.18714200	0.21366800	-0.07869200
C	-2.73393200	0.30763700	-0.70846200
C	0.80115600	1.19057400	0.23037500
C	-1.12388800	2.24235700	-0.23791900
C	-3.40478700	-0.24409900	0.54152200
C	0.19333600	-1.17371600	-0.01178100
C	2.09637000	0.89596700	0.57766600
C	1.45433700	-1.53170400	0.30759300
C	2.54007100	-0.52983600	0.53823800
H	-3.36680900	1.08630500	-1.14227000
H	-2.63206800	-0.49366800	-1.44493600
H	-1.78311900	3.08494900	-0.38175100
H	0.63051000	3.31284200	0.26515900
H	-3.60174800	0.54204700	1.26807000
H	-2.80714800	-1.02646300	1.00485600
H	2.81295300	1.66674200	0.83011600
H	-5.18570800	-1.24567200	1.01031400
H	-4.60240200	-1.60693900	-0.49697000
H	1.71893200	-2.58146500	0.37670000
H	3.12149700	-0.78470300	1.43193600
O	-0.80715200	-2.06902700	-0.26276400
H	-0.45182500	-2.96705500	-0.22420500
O	3.47117600	-0.74527100	-0.58300400
O	4.66777700	-0.02275200	-0.32176200

H	5.19733300	-0.65498500	0.18783700
H	-5.34860100	-0.15565000	-0.22611500

Table S6 Optimized structures of 10-OH-TA forms in gas phase using M06-2X/6-311++G(d,p)

Stable Neutral form			$C_{10}H_{12}N_2O$
0 1			
N	-0.92491800	-1.87075900	0.01855500
N	4.47981900	0.53001200	0.34359800
C	0.97813200	-0.73156500	-0.37237900
C	-0.10590600	0.20941400	-0.24753500
C	2.41893300	-0.39858600	-0.60219100
C	-1.26939900	-0.54446300	-0.00623000
C	0.43129400	-1.97393600	-0.20402000
C	3.08320100	0.20661900	0.63596500
C	-0.20043500	1.61135900	-0.33154800
C	-2.52117800	0.05351000	0.15752200
C	-1.43831500	2.19819500	-0.17026800
C	-2.59608400	1.42891300	0.07520300
H	2.97715000	-1.29093500	-0.89551700
H	2.50305300	0.31343500	-1.43263000
H	0.91195500	-2.94002100	-0.23070200
H	-1.56906700	-2.62960200	0.16370300
H	3.06443500	-0.53366900	1.43982300

H	2.49039900	1.06645200	0.97816100	
H	4.95517600	0.86605700	1.17315600	
H	4.53385500	1.26997300	-0.34867300	
H	0.67859500	2.21623100	-0.52146600	
H	-1.53723000	3.27514300	-0.23132200	
H	-3.55552400	1.92095600	0.19863600	
O	-3.57920300	-0.78016900	0.38617000	
H	-4.38494300	-0.26418200	0.47529700	
**Cation**				**$[C_{10}H_{12}N_2O]^{\bullet+}$**
1 2				
N	-0.85735400	-1.88106700	0.00330900	
N	4.47264600	0.47206000	0.39215300	
C	0.98600600	-0.66092900	-0.41819200	
C	-0.12022800	0.23170400	-0.27322300	
C	2.40931100	-0.31997700	-0.64202800	
C	-1.25950800	-0.55387800	-0.01203900	
C	0.45784100	-1.95772500	-0.23032000	
C	3.06448300	0.26459000	0.63876300	
C	-0.24262300	1.62365200	-0.35519000	
C	-2.51539200	-0.00962200	0.17626800	
C	-1.51426400	2.19184500	-0.16077000	
C	-2.62029400	1.40545600	0.09736100	
H	2.97829500	-1.20145700	-0.94002600	
H	2.49498300	0.41930100	-1.44456300	

H	0.97318900	-2.90651700	-0.26350900
H	-1.47592000	-2.66817600	0.16124000
H	2.95555300	-0.46024700	1.44855300
H	2.50875600	1.16643300	0.93471400
H	5.01152300	0.52025500	1.24710200
H	4.65683400	1.30663500	-0.15139900
H	0.61423100	2.25083500	-0.56944900
H	-1.63380200	3.26596200	-0.21774700
H	-3.59246100	1.86505100	0.24128700
O	-3.53737000	-0.82864000	0.41658900
H	-4.37125400	-0.35659100	0.52907700

**Radical Form (O24-H)**	**$C_{10}H_{11}N_2O^{\bullet}$**

0 2

N	-0.93063100	-1.88245500	0.03156800
N	4.44036500	0.56857600	0.33769700
C	0.95367400	-0.72675200	-0.37435400
C	-0.15174500	0.18984600	-0.24621000
C	2.38802400	-0.37214700	-0.60910900
C	-1.29570700	-0.58076900	0.00450300
C	0.42795500	-1.97867600	-0.19659500
C	3.04412800	0.24912800	0.62664800
C	-0.28164600	1.58594100	-0.33598200
C	-2.62476800	-0.03394900	0.18758400
C	-1.55326500	2.17227500	-0.16488800

C	-2.67647800	1.41607300	0.08640100	
H	2.95836600	-1.25844100	-0.89600200	
H	2.46126600	0.33474800	-1.44438100	
H	0.91806900	-2.93939800	-0.21945000	
H	-1.57728500	-2.64089900	0.18928400	
H	3.02075200	-0.48140700	1.43909000	
H	2.44822000	1.11248400	0.95610600	
H	4.92028700	0.89026900	1.17008500	
H	4.50270600	1.31278400	-0.34901000	
H	0.58071700	2.21098000	-0.53825000	
H	-1.64421100	3.24973500	-0.23501000	
H	-3.64933700	1.87440000	0.21680200	
O	-3.61109000	-0.74924100	0.40723700	
**Anion (O24-H)**				**$C_{10}H_{11}N_2O^-$**
-1 1				
N	-0.98311200	-1.86086200	0.03415400	
N	4.41711600	0.58003300	0.32822100	
C	0.93139400	-0.76684700	-0.35969200	
C	-0.13140800	0.20148100	-0.24210100	
C	2.37748100	-0.45739000	-0.58963000	
C	-1.30273600	-0.52443900	0.00225800	
C	0.36256900	-2.00699900	-0.18413600	
C	3.02862000	0.19497500	0.62942100	
C	-0.20242600	1.61552300	-0.33282300	

C	-2.62647300	0.01466800	0.18043200
C	-1.45872900	2.18244100	-0.16845900
C	-2.62580900	1.43601500	0.07901900
H	2.93720200	-1.36181900	-0.84834300
H	2.47357200	0.23192200	-1.44064000
H	0.82787300	-2.98188100	-0.20529300
H	-1.68016400	-2.57052300	0.18897100
H	3.04401300	-0.52796900	1.44943800
H	2.40243500	1.03708400	0.94954800
H	4.82880300	1.05720600	1.12318400
H	4.41751900	1.24734700	-0.43753300
H	0.67506100	2.22315800	-0.52608100
H	-1.55309800	3.26445700	-0.23315300
H	-3.57760900	1.94399900	0.19837400
O	-3.61856700	-0.74171900	0.39489800

**Radical Form (N1-H)**	$C_{10}H_{11}N_2O^{\bullet}$

0 2

N	-0.88996000	-1.96150900	0.01409700
N	4.46552800	0.49904600	0.37959900
C	0.97199100	-0.67940200	-0.41186800
C	-0.13730300	0.22565300	-0.27025200
C	2.39862900	-0.33424200	-0.63496900
C	-1.25248400	-0.60705200	-0.01199900

C	0.40126100	-1.98140700	-0.21893500
C	3.06204300	0.19975800	0.64610000
C	-0.25758000	1.60863100	-0.35012100
C	-2.50784100	-0.05372900	0.17294600
C	-1.52887300	2.15986700	-0.16135600
C	-2.62721100	1.34907600	0.09482400
H	2.96010800	-1.20684700	-0.97529100
H	2.47640900	0.43392900	-1.41385400
H	0.95089500	-2.91466800	-0.25887100
H	3.01664000	-0.57673600	1.41334100
H	2.48191100	1.05571200	1.01684900
H	4.95776500	0.72358800	1.23624000
H	4.55201400	1.30124000	-0.23520900
H	0.59750500	2.24301300	-0.55428100
H	-1.66739200	3.23262900	-0.21521400
H	-3.60505500	1.79906200	0.23786800
O	-3.56561700	-0.85633700	0.41831200
H	-4.36062900	-0.32589700	0.51983600

**Anion (N1-H)**	**$C_{10}H_{11}N_2O^-$**

-1 1

N	-0.96028300	-1.95821700	0.03047800
N	4.48358600	0.54764600	0.34483600
C	0.96436200	-0.73457000	-0.36950400

C	-0.10920900	0.18509900	-0.24868900
C	2.40825700	-0.41311700	-0.59670900
C	-1.26671700	-0.63418600	-0.00363700
C	0.37175700	-1.98990100	-0.18914600
C	3.08700300	0.17924200	0.63892600
C	-0.22201200	1.59035600	-0.33265100
C	-2.51528400	-0.00585800	0.15612800
C	-1.46437300	2.17016800	-0.17302700
C	-2.60897300	1.37439900	0.07351400
H	2.95967600	-1.31039900	-0.89821600
H	2.51374000	0.31062700	-1.42040100
H	0.89525900	-2.94256700	-0.22088700
H	3.08945000	-0.57281000	1.43223600
H	2.48358400	1.02453200	0.99497700
H	4.89881100	1.02211600	1.13968200
H	4.49552700	1.21055700	-0.42466500
H	0.65203100	2.20858300	-0.52221000
H	-1.57801900	3.24792300	-0.23366100
H	-3.57962500	1.84963800	0.19978700
O	-3.62304300	-0.78806000	0.39164800
H	-4.37806900	-0.19951800	0.46174000

**Radical Form (N2-H)**	$\mathbf{C_{10}H_{11}N_2O^{\bullet}}$
0 2	

N	-0.85669000	-1.87274300	0.03033800
N	4.48102000	0.64035700	0.35244200
C	1.03570000	-0.72586100	-0.38656700
C	-0.05275400	0.20994400	-0.26129000
C	2.47218100	-0.37879700	-0.62043100
C	-1.20938000	-0.54862000	-0.00325900
C	0.49811100	-1.97007100	-0.20215500
C	3.11769800	0.25929000	0.61066000
C	-0.15546300	1.61053500	-0.35892600
C	-2.46315800	0.04362400	0.16588700
C	-1.39535300	2.19154200	-0.19119400
C	-2.54612800	1.41794800	0.07226800
H	3.04067100	-1.27218500	-0.89067600
H	2.56450200	0.32005100	-1.45774900
H	0.98395100	-2.93371000	-0.22430100
H	-1.49587600	-2.63432100	0.18330600
H	3.06673800	-0.43922400	1.46122100
H	2.53449100	1.13907300	0.92851800
H	4.85415300	1.03162400	1.22288600
H	0.71694800	2.21820500	-0.56972400
H	-1.50142700	3.26707200	-0.26346500
H	-3.50725000	1.90573600	0.19926900
O	-3.51411000	-0.79379600	0.41045800
H	-4.32305600	-0.28245300	0.49762800

Anion (N2-H)	$C_{10}H_{11}N_2O^-$		
-1 1			
N	-0.86036100	-1.87645000	0.01624300
N	4.50906000	0.62695800	0.27520300
C	1.04355300	-0.70475600	-0.37300100
C	-0.06507700	0.21065200	-0.24685800
C	2.47545100	-0.33194300	-0.59311500
C	-1.22425500	-0.55682200	-0.00889600
C	0.51043100	-1.95130600	-0.20029700
C	3.18972800	0.23901300	0.65043500
C	-0.17081000	1.61047700	-0.32654800
C	-2.47790800	0.03395900	0.15029200
C	-1.41356300	2.19042200	-0.16918000
C	-2.56732000	1.40927500	0.06839800
H	3.05173100	-1.19718800	-0.93464800
H	2.55287900	0.42935300	-1.37763500
H	1.00899600	-2.90859500	-0.21343300
H	-1.48471500	-2.64297600	0.19261000
H	3.10078700	-0.56666200	1.42809600
H	2.51903500	1.05113900	1.03766000
H	4.96826100	0.99362200	1.11117900
H	0.71777900	2.20862600	-0.49164700
H	-1.51891200	3.26788200	-0.22092200

H -3.53376600 1.89021100 0.19185100<br>O -3.54867900 -0.80111300 0.37853100<br>H -4.33491000 -0.26388700 0.50113900	
**Inter1-O24-HAT**	**$C_{10}H_{12}N_2O + HOO^{\cdot}$**
0 2<br>N 0.65428000 -1.29989100 -1.11042600<br>N -4.94557400 0.13768700 0.15892200<br>C -1.26063000 -0.70429600 -0.08212200<br>C -0.31988400 0.38426500 0.02159300<br>C -2.66581300 -0.71227600 0.43254400<br>C 0.86109200 -0.02960800 -0.63379200<br>C -0.62494900 -1.69457500 -0.77836000<br>C -3.59534300 0.17343100 -0.40028500<br>C -0.36175100 1.66220000 0.61110800<br>C 2.01230800 0.77155100 -0.67587400<br>C 0.75962100 2.46389800 0.53163200<br>C 1.94142300 2.02261200 -0.10019900<br>H -3.06613100 -1.72865400 0.43878400<br>H -2.67889400 -0.36157600 1.47181100<br>H -0.98818100 -2.67059200 -1.06169800<br>H 1.32127500 -1.83619400 -1.64010500<br>H -3.63571400 -0.22265800 -1.41806100<br>H -3.16738000 1.18373500 -0.46657800	

H	-5.59596100	0.64496900	-0.42973800
H	-4.96480700	0.57969500	1.07203800
H	-1.25608000	2.00731800	1.11697300
H	0.74621400	3.45124100	0.97672900
H	2.82276400	2.65096400	-0.13196600
O	3.15356900	0.31964100	-1.28586900
H	3.45331500	-0.45308000	-0.78880800
O	2.98135900	-1.55715600	1.01205200
O	2.01113400	-1.31792400	1.84498400
H	1.40370000	-0.68944400	1.39433400

**TS-O24-HAT**	**$C_{10}H_{12}N_2O + HOO^{\cdot}$**

0 2

N	0.66712900	-1.34166500	-0.65716700
N	-5.08718000	-0.14201500	-0.00216700
C	-1.34851200	-0.73198900	0.14431000
C	-0.48237200	0.42223500	0.11902500
C	-2.78471400	-0.75696500	0.56048000
C	0.76373800	-0.01277400	-0.38069600
C	-0.59945300	-1.77606400	-0.32007500
C	-3.70232600	-0.08946100	-0.46695000
C	-0.63850600	1.75851200	0.49725000
C	1.86887100	0.85841400	-0.51145900
C	0.45102600	2.62603000	0.38697100

C	1.68244600	2.18665100	-0.08839600
H	-3.12124200	-1.78532000	0.71053800
H	-2.89700300	-0.24365200	1.52337000
H	-0.87182800	-2.81322700	-0.44031700
H	1.46127600	-1.93316400	-0.86061200
H	-3.63522800	-0.64288000	-1.40691600
H	-3.33839100	0.92822400	-0.66802800
H	-5.72109300	0.21616100	-0.70692100
H	-5.20757000	0.43205400	0.82573600
H	-1.58638600	2.11708900	0.88294500
H	0.33894000	3.66253100	0.68035200
H	2.52404400	2.86198300	-0.18037400
O	3.00954100	0.47969300	-1.06216700
H	3.30369100	-0.44743500	-0.66823100
O	3.51199800	-1.53206200	0.17346200
O	2.84081800	-1.19561500	1.30929600
H	3.53110800	-0.89025800	1.91631000

**Inter2-O24-HAT**	**$C_{10}H_{12}N_2O + HOO^{\cdot}$**

0 2

N	0.81702300	-1.12049300	-0.19196500
N	-5.07951500	-0.71664700	-0.26433800
C	-1.33770100	-0.72342500	0.31822400
C	-0.62922600	0.52685200	0.23603900

C	-2.79545900	-0.91023800	0.59559100
C	0.70903300	0.22363000	-0.08176100
C	-0.41011400	-1.69548900	0.05018100
C	-3.67177600	-0.50611100	-0.59327800
C	-1.01056900	1.86176900	0.41895900
C	1.72994500	1.23583100	-0.24472500
C	-0.04548500	2.88230000	0.27199000
C	1.25967600	2.59544700	-0.04556800
H	-3.00558900	-1.95331700	0.84208400
H	-3.08469300	-0.31473200	1.46992100
H	-0.52780500	-2.76741000	0.01923900
H	1.68467500	-1.61269200	-0.39467200
H	-3.41999700	-1.14266800	-1.44511000
H	-3.43132900	0.52678100	-0.88414200
H	-5.67108000	-0.55151400	-1.07018300
H	-5.37819400	-0.07489300	0.46240800
H	-2.03341400	2.11433600	0.67486200
H	-0.34535100	3.91356100	0.41412700
H	2.00111500	3.37671100	-0.15975100
O	2.91738400	0.98700900	-0.53392500
H	3.53810900	-0.59877900	-0.65157900
O	3.65167900	-1.57881900	-0.60040700
O	3.69312300	-1.78436500	0.81021700
H	4.62356500	-1.98940200	0.95593000

TS- C7-RAF	$C_{10}H_{12}N_2O + HOO^{\bullet}$

0 2

N	0.93655700	1.12760800	0.89156600
N	-4.55281300	-0.97750100	0.27028200
C	-0.98049000	0.24401200	0.04770400
C	0.06975800	-0.69517700	-0.14970800
C	-2.42034700	0.08234800	-0.29927100
C	1.24737800	-0.09792200	0.35087400
C	-0.38452800	1.40260600	0.58556600
C	-3.14945900	-0.86426900	0.66111500
C	0.12787600	-1.98331300	-0.72107600
C	2.48358200	-0.73885600	0.27380500
C	1.35099800	-2.61380400	-0.78490300
C	2.52341500	-1.99877300	-0.29441200
H	-2.92403200	1.05141700	-0.29098700
H	-2.50581400	-0.31276700	-1.31916300
H	1.62056100	1.86069600	1.01377300
H	-3.10845600	-0.44387100	1.66921900
H	-2.61480600	-1.82405500	0.69104100
H	-5.07038700	-1.54128000	0.93447700
H	-4.63540300	-1.43053800	-0.63383500
H	-0.76549000	-2.45849300	-1.10835000
H	1.42956300	-3.60115400	-1.22276100

H	3.47273500	-2.52006300	-0.36552500
O	3.56184000	-0.06869200	0.77050800
H	4.36061500	-0.58430800	0.63097800
H	-0.89775900	2.20273200	1.09852200
O	-0.19809000	2.50211600	-1.02144100
O	0.60578400	3.54842500	-0.59895900
H	-0.01289800	4.27504300	-0.44935200

**Product-C7-RAF**	**$C_{10}H_{12}N_2OOOH$**

0 2

N	0.85722400	1.43687600	0.39653500
N	-4.25597900	-1.61524400	0.42566600
C	-0.90523400	0.01656700	-0.19503900
C	0.30138600	-0.70200800	-0.26762500
C	-2.28314400	-0.46874600	-0.47153200
C	1.34688000	0.16236900	0.14117000
C	-0.59008000	1.41183200	0.27167300
C	-2.90017800	-1.17605300	0.74508600
C	0.59600500	-2.03370600	-0.62906100
C	2.65363300	-0.28532200	0.23821500
C	1.90671900	-2.46431000	-0.54387400
C	2.92927300	-1.60633500	-0.10710900
H	-2.93088600	0.36228600	-0.76008700
H	-2.26278300	-1.17172800	-1.31257500

H	1.27822100	1.96889600	1.14428300
H	-2.96001200	-0.46690000	1.57522600
H	-2.22837600	-1.98755400	1.05883500
H	-4.71002100	-2.01508600	1.23855900
H	-4.23827700	-2.33368100	-0.29046400
H	-0.19128800	-2.70247900	-0.95542500
H	2.16009900	-3.48287000	-0.81057200
H	3.94905000	-1.97153800	-0.03982700
O	3.58854500	0.60940900	0.67858400
H	4.45345900	0.19188700	0.70221100
H	-1.08189100	1.70906600	1.20726200
O	-1.04843100	2.29947300	-0.74226500
O	-0.77233400	3.62016800	-0.29687600
H	0.08018200	3.79015100	-0.71980200

**TS-C9-RAF**	**$C_{10}H_{12}N_2O + HOO^{\bullet}$**

0 2

N	-0.92338300	-2.20498100	-0.59607800
N	4.48493700	-0.14270400	0.69484200
C	0.97733500	-1.01079400	-0.44061800
C	-0.10211000	-0.22773900	0.06787800
C	2.41008500	-0.58748300	-0.52340400
C	-1.26620800	-0.99443500	-0.05514800
C	0.43107900	-2.21112700	-0.82878100

C	3.09981300	-0.58672300	0.84198500
C	-0.18707400	1.12544600	0.52567100
C	-2.52320000	-0.51630400	0.33701600
C	-1.45236900	1.54942600	1.01161800
C	-2.59500300	0.76544500	0.87742600
H	2.96746100	-1.24414300	-1.19542900
H	2.45171500	0.42205400	-0.94842700
H	0.91125500	-3.07232500	-1.26724000
H	-1.56878800	-2.95086900	-0.79600000
H	3.10663100	-1.60641900	1.23637100
H	2.51063700	0.02314600	1.54197100
H	4.97297200	-0.17384300	1.58242500
H	4.51375000	0.81926500	0.37273900
H	0.70256000	1.62817400	0.88471400
H	-1.53676400	2.53297900	1.45750300
H	-3.55507300	1.14390200	1.21414800
O	-3.58450700	-1.34682800	0.17352300
H	-4.39147200	-0.91279000	0.46512400
O	-0.01346600	2.01251000	-1.15031100
O	0.10090300	3.37021000	-0.90654600
H	-0.79329500	3.70326400	-1.05443900

**Product-C9-RAF**	**$C_{10}H_{12}N_2OOOH$**
0 2	

N	-1.05638400	-2.21552800	-0.42025900
N	4.43032400	-0.30104400	0.65913700
C	0.88468200	-1.09116700	-0.41637400
C	-0.16168500	-0.20752200	-0.03996300
C	2.34112400	-0.75949500	-0.52972900
C	-1.34799500	-0.92940000	-0.05796900
C	0.29476400	-2.31869500	-0.63927200
C	3.02070800	-0.64685000	0.83609600
C	-0.14480800	1.24979700	0.26616700
C	-2.60180700	-0.37022600	0.27199500
C	-1.49736900	1.75099900	0.69005900
C	-2.63207200	0.98198400	0.65321200
H	2.86318100	-1.52028200	-1.11484800
H	2.44875900	0.19170600	-1.06168300
H	0.73953900	-3.25148700	-0.94800900
H	-1.73468600	-2.95476600	-0.50371400
H	2.96519200	-1.61507100	1.34090300
H	2.46500300	0.07081100	1.45659100
H	4.91085100	-0.26934900	1.55086000
H	4.51496700	0.62084400	0.24319800
H	0.61401000	1.50673200	1.01592600
H	-1.55804000	2.77961600	1.02261900
H	-3.58422200	1.41850600	0.94307300
O	-3.68683500	-1.18658900	0.22176200

H	-4.47832300	-0.69928900	0.46782700
O	0.30006000	1.89044800	-0.96125300
O	0.56629000	3.25604700	-0.65684300
H	-0.15317600	3.70224000	-1.11954300

**TS-C10-RAF**	**$C_{10}H_{12}N_2O$ + $HOO^{\cdot}$**

0 2

N	-0.31163500	1.83651400	-0.34973100
N	5.10505800	-0.50565900	0.05264700
C	1.53265600	0.72991800	0.30723100
C	0.47321000	-0.21735200	0.06617300
C	2.93485100	0.41199700	0.72110100
C	-0.64485000	0.52306800	-0.34219600
C	1.00648100	1.96468300	0.04177000
C	3.75088100	-0.21185400	-0.41341800
C	0.37107900	-1.61695900	0.15744600
C	-1.91740000	-0.06545200	-0.58333500
C	-0.83690800	-2.23202800	-0.18332200
C	-1.93621400	-1.49398100	-0.59633500
H	3.44682700	1.31488000	1.06175200
H	2.91892700	-0.28142600	1.57078400
H	1.47233200	2.93606100	0.09905800
H	-0.95261700	2.58098200	-0.57390800
H	3.81939400	0.50899800	-1.23201600

H	3.21439300	-1.08863000	-0.80341600
H	5.68600300	-0.84126600	-0.70675300
H	5.08863900	-1.23390700	0.75893700
H	1.21852900	-2.21422200	0.47382200
H	-0.91161700	-3.31202800	-0.14838700
H	-2.85582800	-1.98365000	-0.89670500
O	-2.81881100	0.67005800	-1.28983100
H	-3.69252900	0.52409800	-0.89241800
O	-2.66767100	0.23744300	1.16856600
O	-4.03751800	0.07062600	0.99083000
H	-4.21713200	-0.80312800	1.36163100

**Product-C10-RAF**	**$C_{10}H_{12}N_2OOOH$**		
0 2			
N	-0.31859100	1.83924300	-0.14548700
N	5.08840000	-0.52313300	-0.16940900
C	1.55489200	0.72889200	0.37876000
C	0.47524300	-0.20950600	0.22222400
C	2.98318700	0.39878900	0.67949700
C	-0.65521800	0.52855100	-0.10124000
C	1.02305300	1.97019500	0.14232400
C	3.70368400	-0.21432800	-0.52351000
C	0.39442800	-1.62329100	0.30835600
C	-2.01203100	-0.02534200	-0.31310200

C	-0.84425800	-2.24034600	0.01780100
C	-1.96831700	-1.53036400	-0.29772000
H	3.52591800	1.29463100	0.98999400
H	3.03031600	-0.30566700	1.51885500
H	1.49531600	2.93958500	0.15573100
H	-0.96183600	2.58373600	-0.36458100
H	3.71256800	0.51716800	-1.33543600
H	3.13152900	-1.08220500	-0.88133300
H	5.60484900	-0.85245600	-0.97672400
H	5.12148400	-1.26129300	0.52595200
H	1.26034800	-2.22024100	0.56477500
H	-0.90032200	-3.32307000	0.02758700
H	-2.88961100	-2.03870900	-0.55857400
O	-2.56350500	0.50329700	-1.48768100
H	-3.49675600	0.25725900	-1.50193400
O	-2.77950100	0.47905100	0.80997000
O	-4.12597500	0.04605300	0.63490900
H	-4.19860200	-0.64821200	1.30202500
**TS-C11-RAF**		**$C_{10}H_{12}N_2O$ + HOO$^{\cdot}$**	
0 2			
N	0.16815700	2.37491800	-0.56293300
N	-4.62711700	-0.91777800	0.53866000
C	-1.40602200	0.76592000	-0.52730400

C	-0.17061100	0.22530400	-0.00923100
C	-2.70853500	0.03992400	-0.64442400
C	0.77372800	1.26956900	-0.05114800
C	-1.14990100	2.06839000	-0.85038800
C	-3.36997800	-0.19276400	0.71604600
C	0.21699300	-1.02458300	0.47609400
C	2.10018400	1.11101100	0.41171500
C	1.58212500	-1.23752100	0.81399200
C	2.47887500	-0.10876700	0.88581000
H	-3.40265100	0.59649200	-1.27831800
H	-2.54566600	-0.93043100	-1.12960300
H	-1.80662100	2.81224900	-1.27407200
H	0.62293900	3.26146600	-0.70874900
H	-3.59514100	0.77788200	1.16506700
H	-2.65580500	-0.69632600	1.38277800
H	-5.12466400	-1.00163400	1.41746700
H	-4.45051000	-1.86029300	0.20695000
H	-0.48159300	-1.85117800	0.53395100
H	1.81754000	-2.09455500	1.43329800
H	3.48947300	-0.26748100	1.24495200
O	2.88235800	2.22503100	0.36082100
H	3.77994900	2.00419500	0.62566400
O	2.26522600	-2.07017800	-0.70044100
O	1.66678500	-3.31535300	-0.82318300

H	0.96937400	-3.16527100	-1.47403500	
**Product-C11-RAF**			**$C_{10}H_{12}N_2OOOH$**	
0 2				
N	-0.22524200	2.41765500	-0.31469400	
N	-4.61199400	-1.49535700	0.40908800	
C	-1.63823300	0.67466700	-0.47928900	
C	-0.32862000	0.18254300	-0.10813300	
C	-2.86838200	-0.15427600	-0.66689800	
C	0.50717100	1.31143700	-0.01944100	
C	-1.52430400	2.03166300	-0.59404400	
C	-3.42551100	-0.67784100	0.65890900	
C	0.17890500	-1.08407700	0.14752600	
C	1.88035000	1.22029700	0.36425100	
C	1.63569600	-1.26446100	0.43558700	
C	2.41579000	0.00936600	0.61416700	
H	-3.64815300	0.42316600	-1.16893600	
H	-2.63697600	-1.00782100	-1.31591300	
H	-2.26946000	2.76403400	-0.86266200	
H	0.13489700	3.35807600	-0.32195500	
H	-3.72109000	0.17503500	1.27526600	
H	-2.62811200	-1.20515600	1.20111600	
H	-5.03967700	-1.78880600	1.27969600	
H	-4.36108900	-2.33857100	-0.09662800	

H	-0.43097300	-1.97571100	0.07742300	
H	1.78299100	-1.91542600	1.30714600	
H	3.44551100	-0.08104500	0.94326500	
O	2.52274000	2.41882600	0.46683600	
H	3.43782500	2.27422500	0.72456000	
O	2.13006100	-2.03339000	-0.69576200	
O	3.45013500	-2.46752800	-0.37147500	
H	3.97429200	-1.97725000	-1.01573900	
**TS-C12-RAF**				**$C_{10}H_{12}N_2O$ + HOO$^{\bullet}$**
0 2				
N	-0.19208200	2.23486800	-0.13233200	
N	-4.96117900	-1.25523000	0.15453900	
C	-1.72745300	0.61926400	-0.46259400	
C	-0.50649800	0.00577100	-0.04614400	
C	-3.02031700	-0.07401300	-0.75935000	
C	0.43034600	1.04357300	0.15095600	
C	-1.48791800	1.97383200	-0.49922100	
C	-3.71092400	-0.58490200	0.50720200	
C	-0.10143700	-1.34152800	0.15103500	
C	1.73547000	0.79500200	0.53841300	
C	1.18204200	-1.60305600	0.53828400	
C	2.15670000	-0.55429300	0.69459100	
H	-3.70493500	0.59830400	-1.28161700	

H	-2.83665900	-0.92154300	-1.43074800
H	-2.15792700	2.77604700	-0.76863200
H	0.25318300	3.13703400	-0.10155500
H	-3.94896400	0.27007400	1.14513300
H	-3.00915300	-1.21764400	1.06902000
H	-5.47545500	-1.52537200	0.98483100
H	-4.77475300	-2.10419700	-0.36891200
H	-0.81175000	-2.14897800	0.01280800
H	1.50224600	-2.62003300	0.72998200
O	2.61447700	1.81437400	0.65320100
H	3.49359700	1.45964000	0.46069200
H	2.99743800	-0.74066300	1.35509900
O	3.34029500	-0.55590500	-0.75716200
O	4.17800000	-1.65629600	-0.60269800
H	3.78372500	-2.31543300	-1.18731000

**Product-C12-RAF**	**$C_{10}H_{12}N_2OOOH$**

0 2

N	-0.25238500	2.27026500	-0.06598200
N	-4.92079300	-1.34185600	0.26914200
C	-1.77241600	0.64709300	-0.42943300
C	-0.51952200	0.03630500	-0.15747200
C	-3.07245300	-0.04950100	-0.68742200
C	0.41774100	1.07480300	0.06278400

C	-1.57036600	2.01070900	-0.36370900
C	-3.65266900	-0.68316900	0.57882500
C	-0.06958100	-1.32470900	-0.10112700
C	1.74267100	0.84074000	0.33640800
C	1.21968400	-1.60901400	0.17845500
C	2.25362700	-0.55363400	0.43709500
H	-3.80712600	0.64945700	-1.09391500
H	-2.92882800	-0.83164900	-1.44266000
H	-2.27494400	2.81416300	-0.51164600
H	0.17339700	3.17750100	0.02327400
H	-3.84646800	0.10738100	1.30823800
H	-2.90096800	-1.35113700	1.02297300
H	-5.35895700	-1.69938400	1.11003000
H	-4.76896000	-2.13403400	-0.34643800
H	-0.77603100	-2.12894700	-0.27996200
H	1.55895800	-2.63575400	0.25071900
O	2.60219600	1.86777000	0.56063000
H	3.49563300	1.56066500	0.36319500
H	2.74604200	-0.71204300	1.40932700
O	3.34738100	-0.59000800	-0.52965400
O	4.17861300	-1.69856000	-0.20688500
H	4.01469400	-2.28643700	-0.95439900

Table S7 Optimized structures of 10-OH-TA forms in water using M06-2X/6-311++G(d,p)

**Stable Cationic form**			**$[C_{10}H_{13}N_2O]^+$**
1 1			
N	-0.97901700	-1.87973500	0.02638300
N	4.41878500	0.57383500	0.34445200
C	0.92503200	-0.74752300	-0.36293600
C	-0.15295400	0.19997900	-0.24454300
C	2.36605400	-0.42386700	-0.60517000
C	-1.32166900	-0.55166300	-0.00346400
C	0.37385000	-1.98923800	-0.19038200
C	2.99543900	0.20974900	0.62753000
C	-0.23327200	1.60378100	-0.33261100
C	-2.57008100	0.06113200	0.15412000
C	-1.46983000	2.19931800	-0.17607300
C	-2.63390900	1.43811700	0.06784800
H	2.91390500	-1.33402500	-0.86332000
H	2.45939100	0.27231100	-1.44589000
H	0.84824900	-2.95893400	-0.21102000
H	-1.61641900	-2.64932700	0.17107800
H	3.00432100	-0.47876200	1.47058500
H	2.48561200	1.12759200	0.91615700
H	4.86248900	1.00358800	1.16009900
H	4.47665700	1.23659800	-0.43398600

H	0.65375700	2.19847900	-0.52018200
H	-1.56218600	3.27726300	-0.23912900
H	-3.59410500	1.92852500	0.18779400
O	-3.64941800	-0.75141000	0.38296000
H	-4.44907600	-0.21426900	0.45438800
H	4.96241300	-0.25517200	0.08799300

**Cation radical**	**$[C_{10}H_{13}N_2O]^{•+}$**

2 2

N	-0.92239100	-1.88967000	0.00968300
N	4.41340600	0.56318500	0.38779800
C	0.93143500	-0.69684800	-0.39778100
C	-0.16454300	0.20586000	-0.27025100
C	2.36254200	-0.35313700	-0.63241900
C	-1.31163800	-0.57288400	-0.01371900
C	0.40266100	-1.97598300	-0.21482500
C	2.98385700	0.21416100	0.64009300
C	-0.26958100	1.59889300	-0.35683300
C	-2.56316900	0.00166300	0.16988000
C	-1.54051700	2.19123900	-0.17532900
C	-2.65217500	1.42564800	0.07996300
H	2.90986200	-1.24667700	-0.94066600
H	2.43631100	0.39172300	-1.43045700
H	0.90410600	-2.93140700	-0.23795000
H	-1.53960100	-2.68045600	0.16894600

H	2.96872900	-0.51230700	1.45033400
H	2.48107300	1.12576900	0.95867000
H	4.84979300	0.95614900	1.22624200
H	4.49482600	1.25453700	-0.36365600
H	0.60062100	2.21073700	-0.56288300
H	-1.63939300	3.26692700	-0.23983300
H	-3.62701000	1.88000800	0.21928000
O	-3.61014200	-0.76902400	0.41903800
H	-4.41780600	-0.24460600	0.53437500
H	4.94928100	-0.26447500	0.11025600

**Radical Form (O24-H)**	**$[C_{10}H_{12}N_2O]^{+\bullet}$**

1 2

N	-0.98696500	-1.89280600	0.03722800
N	4.37361300	0.61142800	0.34233200
C	0.89705900	-0.74212800	-0.36754400
C	-0.20215000	0.17925100	-0.24542500
C	2.33068400	-0.39474100	-0.61422500
C	-1.35178400	-0.58979100	0.00613300
C	0.36852000	-1.99316300	-0.18605900
C	2.95269300	0.23941300	0.62256700
C	-0.31075200	1.57401600	-0.33909100
C	-2.66465700	-0.01842000	0.18338900
C	-1.57872300	2.17572800	-0.17124000
C	-2.70507800	1.42870600	0.07885900

H	2.88871700	-1.29610700	-0.87971000
H	2.40696000	0.30788200	-1.45039700
H	0.85230900	-2.95750600	-0.20289000
H	-1.61620500	-2.67025600	0.19502500
H	2.96324800	-0.45152000	1.46356700
H	2.43598100	1.15319800	0.91174900
H	4.81267100	1.04197900	1.16024400
H	4.42991200	1.27635300	-0.43450600
H	0.56130700	2.18568400	-0.54033500
H	-1.65943300	3.25365400	-0.24338900
H	-3.67263300	1.90065300	0.20626800
O	-3.68087400	-0.71418800	0.40841900
H	4.92274600	-0.21408200	0.08592300

**Anion (O24-H)**	**[$C_{10}H_{12}N_2O$]**

2 1

N	-0.97459700	-1.87397700	0.03821600
N	4.38316000	0.63796600	0.34105900
C	0.92843400	-0.74604800	-0.36500800
C	-0.18975400	0.17702600	-0.24100100
C	2.35452700	-0.38706000	-0.61281600
C	-1.34285800	-0.61733700	0.01389400
C	0.40591700	-1.98607400	-0.18681400
C	2.96534700	0.26418900	0.62284000
C	-0.33167800	1.52769400	-0.34364900

C	-2.70025300	-0.03782800	0.18914700
C	-1.64125200	2.15908900	-0.17410800
C	-2.75018000	1.44485500	0.07366100
H	2.91568400	-1.28764900	-0.87040900
H	2.41357000	0.30966900	-1.45435300
H	0.87085100	-2.95861000	-0.19914400
H	-1.60302100	-2.66162800	0.19546200
H	2.97497600	-0.41887400	1.46998100
H	2.44159500	1.17781800	0.89854400
H	4.81645700	1.07766100	1.15759800
H	4.43999600	1.29691800	-0.44118100
H	0.52441700	2.16047800	-0.55703500
H	-1.68952700	3.23701700	-0.25882400
H	-3.72304200	1.90419500	0.19971300
O	-3.67112500	-0.72355100	0.40658600
H	4.93759500	-0.18755300	0.09516300

**Radical Form (N1-H)**	**$[C_{10}H_{12}N_2O]^{+\bullet}$**

1 2			
N	-0.95966000	-1.96746900	0.02553700
N	4.40226100	0.54772100	0.37392900
C	0.91573000	-0.70613900	-0.39038400
C	-0.17938200	0.20952800	-0.26298300
C	2.34665600	-0.38213100	-0.62644400

C	-1.30819900	-0.60663100	-0.00867100
C	0.33764500	-2.00154700	-0.19821800
C	2.97038800	0.21178300	0.63426600
C	-0.28203600	1.59522100	-0.34958300
C	-2.55452100	-0.03527800	0.16565200
C	-1.55052700	2.16295400	-0.17178000
C	-2.66125100	1.36947900	0.08076400
H	2.89178700	-1.28507600	-0.91188700
H	2.43059600	0.34824800	-1.43843300
H	0.88485400	-2.93540800	-0.23071800
H	2.95219700	-0.49569400	1.46100400
H	2.47325000	1.13312700	0.93188400
H	4.84190400	0.95433300	1.20405700
H	4.48604200	1.22347700	-0.39124200
H	0.58461900	2.21478000	-0.55000600
H	-1.67423000	3.23728200	-0.23115200
H	-3.63891100	1.82045500	0.21701900
O	-3.63082300	-0.82626600	0.41089600
H	-4.42344500	-0.28218300	0.50841700
H	4.93270300	-0.28840300	0.11191800

**Anion (N1-H)**	**[$C_{10}H_{12}N_2O$]**

2 1

N	-0.64759200	-1.89456300	-0.09988500

N	4.62883500	0.36736900	0.20554200
C	0.92672100	-0.21474500	-0.24939000
C	-0.31905200	0.41745400	-0.13955800
C	2.25363300	0.38423300	-0.41454800
C	-1.27847800	-0.62114100	-0.04475600
C	0.61487000	-1.66907800	-0.21338400
C	3.32014100	-0.30563300	0.43281300
C	-0.66372800	1.76922400	-0.09695500
C	-2.60220900	-0.32616400	0.09176900
C	-2.03031100	2.08503000	0.03243300
C	-2.95810200	1.07829800	0.12330000
H	2.50290300	0.24268200	-1.48125200
H	2.22240100	1.45811100	-0.22173200
H	1.35058200	-2.45952400	-0.28334900
H	3.44436200	-1.35177400	0.16306200
H	3.09500000	-0.22344900	1.49365400
H	5.36417300	-0.07324000	0.76558000
H	4.58486600	1.35758200	0.46664000
H	0.09024800	2.54595400	-0.15798800
H	-2.35021100	3.11725200	0.06695000
H	-4.01149900	1.32067600	0.22977900
O	-3.52904600	-1.27947000	0.19546700
H	-4.41624600	-0.90637200	0.30475900
H	4.90616600	0.31336100	-0.77977800

Radical Form (N2-H)				$[C_{10}H_{12}N_2O]^{+\bullet}$
1 2				
N	-0.83096400	-1.88974400	0.00671700	
N	4.44755700	0.61534900	0.37741300	
C	0.98884600	-0.64476900	-0.42232300	
C	-0.12479600	0.23236300	-0.28377700	
C	2.40766700	-0.27494500	-0.65106200	
C	-1.25450000	-0.56851400	-0.01477600	
C	0.48055900	-1.94840700	-0.22839200	
C	3.05014300	0.26299700	0.63461500	
C	-0.25234800	1.62346600	-0.36907800	
C	-2.50971600	-0.02848600	0.17854200	
C	-1.52698800	2.18280900	-0.17481500	
C	-2.62399900	1.38729600	0.09087100	
H	2.96448000	-1.15084600	-0.99344500	
H	2.46691200	0.49766900	-1.42249700	
H	1.00747500	-2.89043600	-0.25512300	
H	-1.43030600	-2.69277700	0.17462600	
H	2.93397500	-0.48626500	1.42614800	
H	2.51436400	1.16046300	0.95007700	
H	4.85062300	0.99796700	1.22791400	
H	0.60616800	2.24890300	-0.58252700	
H	-1.65579400	3.25570100	-0.23516600	

H	-3.60501800	1.82579000	0.23910700
O	-3.54792500	-0.82920400	0.43582900
H	-4.36158800	-0.31709600	0.55215400
H	4.96674700	-0.23754700	0.18435900

**Anion (N2-H)**	**[$C_{10}H_{12}N_2O$]**

2 1

N	-0.58599200	-1.84062100	-0.10819800
N	4.58629000	0.45887700	0.34208600
C	1.00005900	-0.26091100	-0.40536400
C	-0.22291800	0.39042800	-0.22851200
C	2.34278300	0.26867000	-0.60191300
C	-1.22310300	-0.58770500	-0.04052800
C	0.68510100	-1.70484000	-0.31447200
C	3.24368500	-0.03065900	0.62748500
C	-0.53639700	1.76063700	-0.22271800
C	-2.53221700	-0.26110000	0.16012600
C	-1.88125000	2.13068000	-0.01552700
C	-2.83588600	1.16777600	0.16676600
H	2.78661900	-0.25725500	-1.45870300
H	2.31634700	1.33993900	-0.79424000
H	1.36091100	-2.54438800	-0.40641400
H	-1.06525600	-2.73959900	-0.01548900
H	3.20333800	-1.10163600	0.84865200

H	2.84156100	0.50817300	1.48519500
H	5.13929600	0.40021800	1.19226000
H	0.23700400	2.50538600	-0.37495900
H	-2.15908200	3.17532100	-0.00232300
H	-3.87418100	1.44405000	0.32587800
O	-3.46422000	-1.16977500	0.33507100
H	-4.34292200	-0.77668800	0.46148300
H	5.02890100	-0.16097300	-0.33118400

**Inter1-O24-HAT**	**$[C_{10}H_{13}N_2O]^+$ + HOO$^{\bullet}$**

1 2

N	0.49140700	-1.97416100	-0.30647100
N	-4.84843000	0.57944400	0.02104500
C	-1.28355200	-0.71672700	0.26293500
C	-0.23480600	0.15573500	-0.19446800
C	-2.64898800	-0.30568900	0.71690400
C	0.85921000	-0.67145500	-0.53825800
C	-0.79545600	-1.99191700	0.17412900
C	-3.49599400	0.15809400	-0.45956600
C	-0.13077400	1.55318800	-0.32503800
C	2.06672700	-0.12749300	-0.99948000
C	1.05953400	2.08005300	-0.78788900
C	2.15474900	1.24985700	-1.11603200
H	-3.14229900	-1.14853000	1.20818200

H	-2.57715700	0.51004500	1.44427000
H	-1.27209800	-2.92836800	0.42267700
H	1.06330800	-2.79427700	-0.45239800
H	-3.64913900	-0.64217400	-1.18111900
H	-3.05560200	1.01793200	-0.96162700
H	-5.43483100	0.90221100	-0.75233800
H	-4.77110600	1.34168400	0.70041700
H	-0.96465700	2.19587400	-0.06626900
H	1.17248200	3.15194000	-0.89872000
H	3.08155200	1.67861300	-1.47930900
O	3.15268600	-0.88777600	-1.34440000
H	3.02140900	-1.81531000	-1.11301800
O	2.64676300	-0.24526000	2.16857700
O	2.28644900	1.00121600	2.06726800
H	2.36516900	1.24623800	1.11670500
H	-5.32896000	-0.20016600	0.47953700

**TS-O24-HAT**	**$[C_{10}H_{13}N_2O]^+ + HOO^{\cdot}$**

1 2

N	0.49257500	-1.62663500	-0.67839300
N	-5.07093200	0.26347400	0.18568000
C	-1.41420400	-0.76713700	0.14920200
C	-0.44783000	0.29305200	0.00781700
C	-2.81750400	-0.63434200	0.65090400

C	0.72497300	-0.29676400	-0.51186900
C	-0.79624300	-1.90982400	-0.28061300
C	-3.68575000	0.10822000	-0.35512200
C	-0.45898000	1.66561600	0.28753100
C	1.89600200	0.45166300	-0.74966600
C	0.69305100	2.41635300	0.04826000
C	1.85205700	1.82453100	-0.45274100
H	-3.23913100	-1.62618700	0.83282100
H	-2.82538800	-0.08694000	1.59939800
H	-1.17483400	-2.91931600	-0.33217000
H	1.16146800	-2.29747500	-1.03246300
H	-3.76876800	-0.43886200	-1.29239200
H	-3.30901500	1.11025800	-0.55344800
H	-5.67210000	0.76656300	-0.47169800
H	-5.06167000	0.78204200	1.06897300
H	-1.34868700	2.13844800	0.68908700
H	0.69241500	3.47812500	0.26184800
H	2.74640800	2.41086100	-0.62984200
O	2.99135900	-0.12738900	-1.25268100
H	3.48969600	-0.63592400	-0.45943000
O	3.89083400	-1.01754200	0.79215900
O	3.01241000	-0.34465100	1.57148400
H	3.42372000	0.51958100	1.75283600
H	-5.49588300	-0.65059500	0.36554000

Inter2-O24-HAT	$[C_{10}H_{13}N_2O]^+ + HOO^{\cdot}$

1 2			
N	0.81933700	-1.13451300	-0.26175000
N	-5.08642900	-0.60346500	-0.20984100
C	-1.32091500	-0.72504000	0.27726900
C	-0.60458500	0.52090500	0.21301100
C	-2.77117200	-0.91105000	0.59215000
C	0.72694000	0.21051300	-0.12271800
C	-0.40943800	-1.70421000	-0.02091400
C	-3.64308900	-0.43618900	-0.56199800
C	-0.97628500	1.85531000	0.42157700
C	1.75071100	1.21396600	-0.26477200
C	-0.00010500	2.86914900	0.28419100
C	1.29975600	2.57378000	-0.04437800
H	-2.97096700	-1.96748900	0.78789000
H	-3.03312500	-0.34621800	1.49258700
H	-0.53797400	-2.77423900	-0.07540300
H	1.67298000	-1.64158500	-0.47754500
H	-3.46461000	-1.01883800	-1.46375600
H	-3.48930100	0.61984800	-0.77775300
H	-5.69212100	-0.28693000	-0.97102300
H	-5.32238400	-0.06342100	0.62772900
H	-1.99548000	2.11121700	0.68758400

H	-0.28939900	3.90069300	0.44384400	
H	2.04412600	3.35489400	-0.14713000	
O	2.94624000	0.95067700	-0.55410200	
H	3.57716200	-0.62347900	-0.64004600	
O	3.84892300	-1.56922500	-0.54733000	
O	3.89660800	-1.72446400	0.86662100	
H	4.82959900	-1.57113700	1.07234900	
H	-5.30394000	-1.58618600	-0.02019300	
**TS-C7-RAF**				**$[C_{10}H_{13}N_2O]^+$ + HOO$^\cdot$**
1 2				
N	-0.99196200	1.15527100	-0.89947200	
N	4.44348500	-1.14058900	-0.25885200	
C	0.91592000	0.24110100	-0.09359000	
C	-0.14580000	-0.67167000	0.14601300	
C	2.35525100	0.07420000	0.25607800	
C	-1.32259700	-0.06245100	-0.35041700	
C	0.33542800	1.40183200	-0.65559700	
C	3.01211000	-0.96136300	-0.64973200	
C	-0.21165300	-1.94914700	0.74406900	
C	-2.56480200	-0.69254900	-0.25899400	
C	-1.44324800	-2.56309700	0.82565700	
C	-2.61236500	-1.94375800	0.32999000	
H	2.87388000	1.03061600	0.15302600	

H	2.44865300	-0.25429600	1.29725900
H	-1.65461000	1.85278700	-1.20875500
H	3.00175000	-0.64564100	-1.69095800
H	2.53336700	-1.93495500	-0.56109600
H	4.90380000	-1.83575800	-0.85179600
H	4.51889700	-1.45242300	0.71385000
H	0.68295800	-2.42557600	1.12768400
H	-1.53186300	-3.54267200	1.27958900
H	-3.57026300	-2.44712600	0.40845900
O	-3.65034200	-0.02909700	-0.76104900
H	-4.44520900	-0.56554700	-0.64353500
H	0.84592000	2.19309300	-1.18382700
O	0.34511100	2.50325300	1.01261700
O	-0.42393400	3.61451000	0.69418400
H	0.19797100	4.23587100	0.28640700
H	4.95794600	-0.25906400	-0.34492100

**Product-C7-RAF**	**$[C_{10}H_{13}N_2O]^{+}OOH$**

1 2

N	0.84877400	1.47444100	0.36225000
N	-4.16339800	-1.69113900	0.47349600
C	-0.82813700	-0.04251400	-0.24731200
C	0.41087300	-0.70386700	-0.28954200
C	-2.17531200	-0.60940900	-0.52405000

C	1.40371800	0.21670000	0.13111400
C	-0.59411200	1.36156000	0.23351200
C	-2.80635800	-1.13453600	0.76345900
C	0.76858400	-2.02707300	-0.62714600
C	2.72918300	-0.17084900	0.24994900
C	2.09751000	-2.39423400	-0.51288300
C	3.07222100	-1.48221100	-0.07367100
H	-2.82760900	0.15526300	-0.95642900
H	-2.09266500	-1.43131700	-1.24121600
H	1.20081100	1.99285300	1.15799300
H	-2.93552900	-0.34425600	1.50070300
H	-2.21565000	-1.94118300	1.19369000
H	-4.59881600	-2.06918700	1.31894400
H	-4.10528700	-2.44551300	-0.21670200
H	0.01478900	-2.73279700	-0.95565400
H	2.40328800	-3.40396900	-0.75915200
H	4.10898700	-1.78882200	0.01610000
O	3.63205400	0.76723700	0.68362000
H	4.51471000	0.37665500	0.71772900
H	-1.10706900	1.61478500	1.16762900
O	-1.11353200	2.23425000	-0.77644700
O	-1.09295400	3.56241000	-0.27067000
H	-0.25013700	3.90947700	-0.60088000
H	-4.78100500	-0.96767500	0.09440900

TS-C9-RAF	$[C_{10}H_{13}N_2O]^+ + HOO^{\cdot}$

1 2			
N	-0.88234000	-2.22569700	-0.63186400
N	4.44121900	0.00577500	0.73981000
C	0.97891500	-0.97795300	-0.47765600
C	-0.11591800	-0.22832900	0.04720100
C	2.40555800	-0.53185400	-0.55523300
C	-1.25876300	-1.03318800	-0.07234300
C	0.46334500	-2.19228600	-0.87411400
C	3.04037000	-0.51112400	0.82829800
C	-0.23464500	1.10823100	0.53650100
C	-2.52098300	-0.59977800	0.36151200
C	-1.50210900	1.48845500	1.05482100
C	-2.61845400	0.67405600	0.93342900
H	2.97168200	-1.20372900	-1.20522100
H	2.45689400	0.47426400	-0.98439500
H	0.96351300	-3.03902700	-1.31924700
H	-1.49714500	-3.00293300	-0.83140500
H	3.09420800	-1.50938000	1.25864800
H	2.50309500	0.14731400	1.50899500
H	4.88901100	0.02389000	1.65952900
H	4.45337100	0.95716600	0.36093200
H	0.65040700	1.64650600	0.85301200

H	-1.60958500	2.46455100	1.51239500
H	-3.58625900	1.00584700	1.29362900
O	-3.56542500	-1.44470400	0.21434900
H	-4.37504100	-1.04805600	0.56535900
O	-0.21008600	2.05730200	-1.19152900
O	-0.16930000	3.42953300	-0.94333100
H	-1.09942500	3.69144600	-0.88027300
H	5.00771000	-0.58284100	0.12200700

**Product-C9-RAF**	**$[C_{10}H_{13}N_2O]^{+}OOH$**

1 2

N	-0.98890100	-2.23978500	-0.46912500
N	4.40106100	-0.12328400	0.72014300
C	0.90409500	-1.04354400	-0.45083000
C	-0.17217000	-0.20170900	-0.05759900
C	2.35142500	-0.66869700	-0.55046000
C	-1.33084000	-0.97266300	-0.08242500
C	0.36111000	-2.28970700	-0.69202200
C	2.97057400	-0.54623300	0.83478800
C	-0.21334100	1.24983700	0.26224500
C	-2.59669300	-0.46773800	0.29850000
C	-1.55236300	1.67085300	0.79172900
C	-2.65953600	0.86336400	0.75139700
H	2.89350700	-1.42682200	-1.12176300

H 2.45776100 0.28649400 -1.07393300<br>H 0.83845800 -3.20326000 -1.01103100<br>H -1.62974300 -3.01624500 -0.55935600<br>H 2.96057400 -1.49840400 1.36196600<br>H 2.46632200 0.20608500 1.43904700<br>H 4.83963700 -0.04450000 1.64094500<br>H 4.47427700 0.78564700 0.25408200<br>H 0.59996200 1.55896100 0.92691600<br>H -1.64041600 2.67837000 1.17982600<br>H -3.61872000 1.23546700 1.09933700<br>O -3.66442800 -1.30567300 0.24780500<br>H -4.45850700 -0.84732600 0.55510500<br>O 0.06817500 1.92071400 -1.01923600<br>O 0.25320400 3.30855500 -0.77292800<br>H -0.63518400 3.67653000 -0.89611800<br>H 4.93776300 -0.80085800 0.17078700	
**TS-C10-RAF**	**$[C_{10}H_{13}N_2O]^+$ + $HOO^{\cdot}$**
1 2<br>N 0.34206500 -1.87793800 -0.34227900<br>N -5.02513400 0.60133800 0.02722400<br>C -1.48392800 -0.74250000 0.30775600<br>C -0.41740300 0.19209100 0.05700200<br>C -2.87939200 -0.40529400 0.72958800	

C	0.69201800	-0.56628800	-0.34911500
C	-0.97546100	-1.98676700	0.04805800
C	-3.65145300	0.22030300	-0.42385300
C	-0.31718800	1.59412000	0.12620300
C	1.96132700	0.02863600	-0.60142400
C	0.89179700	2.20348300	-0.22833800
C	1.98833600	1.45825600	-0.63021100
H	-3.39499700	-1.30952200	1.06283200
H	-2.85961700	0.29885700	1.56802900
H	-1.45049700	-2.95385900	0.10834500
H	0.94742900	-2.64916900	-0.59067700
H	-3.76891400	-0.47807200	-1.25032700
H	-3.17341100	1.12970400	-0.78434000
H	-5.56445700	1.01700300	-0.73630200
H	-4.98072600	1.28137000	0.79152400
H	-1.16642100	2.19337200	0.43436000
H	0.97061700	3.28350100	-0.20647500
H	2.91771000	1.93778100	-0.91823800
O	2.87176300	-0.71905400	-1.27535900
H	3.73730100	-0.28490800	-1.23445100
O	2.69487300	-0.20028300	1.20583100
O	4.06621800	0.01492900	1.07779400
H	4.19014500	0.94649200	1.31293500
H	-5.53743000	-0.21733200	0.36804200

Product-C10-RAF	$[C_{10}H_{13}N_2O]^+OOH$

1 2

N	-0.36556000	-1.85494900	0.14460400
N	5.02611700	0.57662300	0.18826600
C	1.50675500	-0.74243700	-0.36906800
C	0.42927200	0.19839300	-0.21975800
C	2.93459900	-0.41589900	-0.67771300
C	-0.70463000	-0.54126100	0.09754000
C	0.97498100	-1.98514100	-0.13456200
C	3.61671300	0.21126300	0.53002400
C	0.36045400	1.61380200	-0.31563300
C	-2.05626000	0.03034700	0.30911800
C	-0.88092700	2.23717900	-0.05197700
C	-2.01107000	1.53310000	0.25810200
H	3.46971600	-1.32492800	-0.96433500
H	2.98794300	0.28409000	-1.51801400
H	1.44425700	-2.95654200	-0.14518500
H	-0.99890500	-2.61734400	0.34807500
H	3.66076400	-0.48143300	1.36854100
H	3.11920400	1.12751100	0.84433600
H	5.50259400	1.00448300	0.98618400
H	5.05099100	1.24138100	-0.59025100
H	1.23479900	2.20122700	-0.56630500

H	-0.93776900	3.31940600	-0.08568400
H	-2.94316600	2.04235200	0.47743100
O	-2.60294600	-0.45693200	1.51133900
H	-3.46482400	-0.03686200	1.64344000
O	-2.84459800	-0.49914300	-0.79188300
O	-4.18878200	-0.06207700	-0.63968200
H	-4.23759100	0.70537000	-1.23038300
H	5.55936100	-0.25128300	-0.09258100
**TS-C11-RAF**		**$[C_{10}H_{13}N_2O]^+ + HOO^\bullet$**	
1 2			
N	-0.27925900	-2.37868700	-0.57812200
N	4.55978700	0.85566000	0.58074300
C	1.33531500	-0.81423000	-0.53030300
C	0.11539100	-0.24331000	-0.00819400
C	2.65303500	-0.11720400	-0.65458400
C	-0.85830700	-1.26662200	-0.05979200
C	1.04594300	-2.10715000	-0.86201100
C	3.28190800	0.09128200	0.71602300
C	-0.22984800	1.01180400	0.48616200
C	-2.18189900	-1.07067500	0.41061800
C	-1.59012900	1.25607700	0.83299900
C	-2.52306200	0.16044600	0.89078800
H	3.32822900	-0.70731700	-1.27912600

H	2.51557900	0.85757400	-1.13440200
H	1.67930700	-2.86924900	-1.28957200
H	-0.74313200	-3.26385700	-0.73287700
H	3.52535200	-0.85666900	1.19223800
H	2.63534400	0.66990300	1.37377900
H	5.00329000	1.00114300	1.49085400
H	4.39248500	1.77351400	0.15888900
H	0.49532400	1.81553700	0.54342200
H	-1.80650100	2.12269900	1.44582700
H	-3.52492100	0.33080600	1.26814100
O	-3.00344800	-2.15630100	0.37031600
H	-3.87659000	-1.92438800	0.71469600
O	-2.20917200	2.12981500	-0.73970200
O	-1.57853500	3.37210800	-0.84718800
H	-0.77133200	3.18937700	-1.35022700
H	5.22302400	0.35462400	-0.01762800

**Product-C11-RAF**	**$[C_{10}H_{13}N_2O]^{+}OOH$**

1 2			
N	-0.12745800	2.43443100	-0.32806900
N	-4.54750500	-1.45672300	0.43462900
C	-1.57966200	0.72676100	-0.48086000
C	-0.28378600	0.20247400	-0.10647000
C	-2.82847500	-0.07268700	-0.67526700

C	0.57974600	1.31554600	-0.02424700
C	-1.43307500	2.08042400	-0.60519200
C	-3.35708100	-0.58030800	0.65948800
C	0.18570600	-1.07599800	0.16307300
C	1.94746200	1.18663300	0.37779900
C	1.63621300	-1.28744400	0.44748600
C	2.44505300	-0.03906800	0.64268700
H	-3.59250500	0.54011700	-1.16020800
H	-2.62506300	-0.93205400	-1.32378800
H	-2.15698700	2.83239900	-0.87898900
H	0.24265200	3.37513300	-0.34807700
H	-3.67849300	0.23869200	1.30021300
H	-2.61745900	-1.18204100	1.18516000
H	-4.92165800	-1.81451900	1.31730900
H	-4.30085400	-2.25894100	-0.15207900
H	-0.45998700	-1.94512800	0.12593000
H	1.77786100	-1.97359500	1.28950900
H	3.47025400	-0.14116900	0.98242600
O	2.63515300	2.36130100	0.48959400
H	3.54976800	2.18076200	0.74460400
O	2.12240400	-2.03738100	-0.72566200
O	3.42382900	-2.53846600	-0.43929600
H	4.00898900	-1.84324300	-0.77654800
H	-5.29547100	-0.94200600	-0.03943200

TS-C12-RAF	$[C_{10}H_{13}N_2O]^+ + HOO^{\cdot}$

1 2			
N	0.09236400	2.26433300	0.24031300
N	4.84399100	-1.23556700	-0.23591500
C	1.61655200	0.62995300	0.49483300
C	0.40199100	0.04052200	0.03902000
C	2.90866300	-0.07498000	0.76750400
C	-0.53306900	1.09002700	-0.10449700
C	1.37905700	1.98471900	0.60174900
C	3.57045700	-0.51104100	-0.53243400
C	0.02158400	-1.29289600	-0.28669900
C	-1.82752100	0.86345200	-0.55232700
C	-1.25392800	-1.53222000	-0.70634800
C	-2.24542600	-0.48400100	-0.74784200
H	3.58431800	0.58895200	1.31281500
H	2.73250600	-0.95854300	1.38958400
H	2.04586600	2.77276000	0.91808100
H	-0.33630900	3.17963100	0.24793200
H	3.82627800	0.34298200	-1.15676400
H	2.93937600	-1.19569100	-1.09679200
H	5.30193900	-1.55212900	-1.09429600
H	4.66632800	-2.05716100	0.34920000
H	0.74702700	-2.09619300	-0.22054300

H	-1.56777500	-2.53299700	-0.97713400
O	-2.66635000	1.91300800	-0.69828300
H	-3.53474100	1.61314100	-1.00159500
H	-3.13174800	-0.63798400	-1.35476300
O	-3.22325100	-0.70317300	0.86069500
O	-3.89771600	-1.92767900	0.76907400
H	-3.25816800	-2.57666500	1.09683500
H	5.49899700	-0.62992600	0.26701100

**Product-C12-RAF**	**$[C_{10}H_{13}N_2O]^+OOH$**

1 2

N	-0.16566200	2.27966000	-0.13116600
N	-4.85591600	-1.29088000	0.29479800
C	-1.68100500	0.65136400	-0.46738100
C	-0.43777500	0.04267600	-0.14773800
C	-2.98191400	-0.04459500	-0.72449900
C	0.49816500	1.08651500	0.05175900
C	-1.47404900	2.01643300	-0.44804400
C	-3.55919500	-0.59936800	0.57032900
C	-0.01904800	-1.31805800	0.03340700
C	1.81118000	0.85153900	0.39910000
C	1.26146900	-1.59629000	0.36061900
C	2.32281900	-0.54226700	0.47693800
H	-3.69488300	0.65639900	-1.16642300

H	-2.83933600	-0.86817400	-1.43148900
H	-2.16857500	2.81946800	-0.64190000
H	0.25191100	3.19744700	-0.07309500
H	-3.76668200	0.19354800	1.28644600
H	-2.89753900	-1.33403100	1.02631800
H	-5.25934700	-1.68413400	1.14906400
H	-4.72546700	-2.05563400	-0.37376000
H	-0.74449100	-2.11863800	-0.07179500
H	1.58210500	-2.61866600	0.52523200
O	2.64504200	1.89791200	0.65641400
H	3.53995400	1.57652200	0.83055000
H	2.93501200	-0.67598700	1.37782200
O	3.28298700	-0.64503700	-0.63970700
O	4.11770800	-1.77448100	-0.41735700
H	3.66552800	-2.48453000	-0.89796800
H	-5.53691200	-0.63989800	-0.10617600

Table S8 Optimized structures of 11-OH-TA forms in gas phase using M06-2X/6-311++G(d,p)

**Stable Neutral form**			**$C_{10}H_{12}N_2O$**
0 1			
N	-0.41431200	2.47377500	-0.10575900
N	4.07934600	-1.37864200	-0.25224000
C	1.06136900	0.83549200	0.34831000
C	-0.24358400	0.25284900	0.18053600

C	2.33506700	0.10125100	0.62716000	
C	-1.14168300	1.30309000	-0.10035400	
C	0.90338400	2.18167800	0.16550100	
C	2.84535000	-0.67010200	-0.59160000	
C	-0.72664100	-1.06503700	0.25796000	
C	-2.50301800	1.07138300	-0.30943600	
C	-2.07370400	-1.28858300	0.05205000	
C	-2.95779900	-0.22947800	-0.23197900	
H	3.11501000	0.79496700	0.95023600	
H	2.18003200	-0.60385800	1.45370200	
H	1.64253500	2.96696200	0.21365800	
H	-0.78676900	3.39354700	-0.26566800	
H	3.06892900	0.04404200	-1.38810700	
H	2.04512900	-1.32477900	-0.96508000	
H	-3.18821800	1.88288900	-0.52504000	
H	4.47051500	-1.83768200	-1.06663100	
H	3.89631300	-2.10085200	0.43656900	
H	-4.00290600	-0.46654700	-0.38533700	
H	-0.05551600	-1.89087100	0.47674800	
O	-2.63459700	-2.53693400	0.10815600	
H	-1.95596600	-3.18353200	0.31680300	
**Cation**				**$[C_{10}H_{12}N_2O]^{\bullet+}$**
1 2				
N	0.46239100	2.41533200	0.10295000	

N	-4.14056400	-1.28990400	0.25859700
C	-1.07855800	0.82693100	-0.35361700
C	0.23955200	0.21827700	-0.18501000
C	-2.35639100	0.10885600	-0.63056100
C	1.16494100	1.28048100	0.10027500
C	-0.89219400	2.15580600	-0.16968100
C	-2.86430100	-0.68176400	0.58716400
C	0.70076400	-1.06675000	-0.26625000
C	2.55118400	1.05511000	0.31577100
C	2.09329300	-1.29076800	-0.05164000
C	3.00137100	-0.22739100	0.23939900
H	-3.13018100	0.81999700	-0.92335900
H	-2.21762400	-0.57551600	-1.47513300
H	-1.59885900	2.96980300	-0.21061800
H	0.84442900	3.33797700	0.26982500
H	-3.01829700	0.01604100	1.41341400
H	-2.09064100	-1.39454100	0.91325700
H	3.22455000	1.87562800	0.53176200
H	-4.63479800	-1.60197200	1.08515600
H	-4.03809500	-2.08502800	-0.36129700
H	4.04183000	-0.48334600	0.39095000
H	0.04290700	-1.90125800	-0.48869800
O	2.62323200	-2.49296000	-0.11013100
H	1.97817300	-3.18483400	-0.31014200

Radical Form (O24-H)				$C_{10}H_{11}N_2O^{\bullet}$
0 2				
N	0.53961500	2.40029100	0.11103300	
N	-4.10233400	-1.27620300	0.24930300	
C	-1.02223300	0.84254800	-0.34460700	
C	0.26574800	0.19787000	-0.18492300	
C	-2.32156300	0.15724300	-0.62253300	
C	1.21211700	1.21896200	0.09905400	
C	-0.80596900	2.17253900	-0.15678900	
C	-2.84211000	-0.61925700	0.59010200	
C	0.68739400	-1.11658500	-0.27155700	
C	2.58738600	0.95235000	0.30903000	
C	2.08073200	-1.44032300	-0.06601100	
C	3.00186400	-0.34140200	0.22883800	
H	-3.08051100	0.88259200	-0.92401100	
H	-2.19473200	-0.53760200	-1.46139100	
H	-1.50206700	2.99597800	-0.19487000	
H	0.95192500	3.30337400	0.27839600	
H	-3.03273100	0.08727300	1.40187900	
H	-2.06157400	-1.30860200	0.94098900	
H	3.28042400	1.75777800	0.52516100	
H	-4.51445400	-1.71403200	1.06499200	
H	-3.94682700	-2.01044800	-0.43360200	
H	4.03869500	-0.61592600	0.37912500	

H	0.01221100	-1.93530900	-0.49360000	
O	2.49105900	-2.61467000	-0.13612900	
**Anion (O24-H)**				**$C_{10}H_{11}N_2O^-$**
-1 1				
N	0.49631600	2.46788300	0.15898300	
N	-3.98867200	-1.40302700	0.25098500	
C	-1.01798100	0.88174500	-0.34938800	
C	0.26826500	0.23447300	-0.18325500	
C	-2.30799600	0.17534800	-0.61862300	
C	1.19142300	1.25598800	0.10685900	
C	-0.82458100	2.22283000	-0.15979100	
C	-2.79290200	-0.61660600	0.59560700	
C	0.67641500	-1.10430800	-0.26685400	
C	2.54331500	0.95604800	0.31198000	
C	2.04406500	-1.47353600	-0.07634100	
C	2.94243000	-0.36079700	0.21661400	
H	-3.09008000	0.87882800	-0.92158000	
H	-2.16477600	-0.52681800	-1.45157400	
H	-1.54018200	3.03114900	-0.21086300	
H	0.93201000	3.37052600	0.08730100	
H	-3.06214700	0.08554500	1.38960700	
H	-1.96173800	-1.22859900	0.96590800	
H	3.26268900	1.73934200	0.54049900	
H	-4.27714300	-1.96208100	1.04705900	

H	-3.74618500	-2.06238000	-0.48293000
H	3.98503500	-0.62457100	0.36647800
H	-0.03454700	-1.89618200	-0.48637200
O	2.47610500	-2.66073200	-0.14676900

**Radical Form (N1-H)**	**$C_{10}H_{11}N_2O^{\bullet}$**

0 2

N	0.33718300	2.54575700	0.12148000
N	-4.09268700	-1.35498500	0.27490600
C	-1.06008600	0.78652200	-0.38016200
C	0.25807900	0.23851900	-0.20030600
C	-2.32078400	0.05430600	-0.65706900
C	1.08096400	1.34503500	0.10321800
C	-0.89220200	2.20168200	-0.15859900
C	-2.84059300	-0.67672400	0.59306500
C	0.78408200	-1.04757800	-0.27901400
C	2.43057700	1.17632700	0.33391900
C	2.15356500	-1.20798500	-0.04376900
C	2.96379600	-0.11459500	0.25881700
H	-3.09861800	0.73757600	-1.00428100
H	-2.15298000	-0.68563800	-1.44930900
H	-1.68776600	2.93576400	-0.21537900
H	-3.03952000	0.06219000	1.37279400
H	-2.05165100	-1.34108300	0.97152900

H	3.06073700	2.02606900	0.56641000
H	-4.52727500	-1.72758500	1.11083300
H	-3.93221400	-2.13447700	-0.35395400
H	4.01735500	-0.29479000	0.43182100
H	0.16057000	-1.90531100	-0.51716900
O	2.75989800	-2.42947500	-0.09802800
H	2.11632800	-3.10539300	-0.32437400

**Anion (N1-H)**	$C_{10}H_{11}N_2O^-$

-1 1

N	0.43824500	2.55750200	0.12177600
N	-4.09643800	-1.37605100	0.24716500
C	-1.05067700	0.84305600	-0.34098000
C	0.23174400	0.26456000	-0.18172000
C	-2.33966700	0.13548000	-0.61805300
C	1.11156800	1.36816400	0.10140000
C	-0.83718000	2.21710700	-0.14224500
C	-2.86686000	-0.63957300	0.59039000
C	0.75263800	-1.04661900	-0.25387700
C	2.47637800	1.12648300	0.30623800
C	2.10391200	-1.23976300	-0.04805300
C	2.97096800	-0.16790600	0.23329400
H	-3.10950500	0.84787700	-0.93435200
H	-2.21523400	-0.57782200	-1.44892700

H	-1.60806100	2.98284200	-0.19417200
H	-3.10236200	0.07099600	1.38688000
H	-2.06435600	-1.28963300	0.96379700
H	3.14007300	1.95881800	0.51996500
H	-4.38767300	-1.96107100	1.02313800
H	-3.90300100	-2.00126200	-0.52954200
H	4.02203600	-0.38328600	0.38783600
H	0.10696600	-1.89858400	-0.46875700
O	2.67915100	-2.50707400	-0.10652500
H	1.97831600	-3.12836600	-0.31461300

**Radical Form (N2-H)**	**$C_{10}H_{11}N_2O^{\bullet}$**

0 2

N	0.33718300	2.54575700	0.12148000
N	-4.09268700	-1.35498500	0.27490600
C	-1.06008600	0.78652200	-0.38016200
C	0.25807900	0.23851900	-0.20030600
C	-2.32078400	0.05430600	-0.65706900
C	1.08096400	1.34503500	0.10321800
C	-0.89220200	2.20168200	-0.15859900
C	-2.84059300	-0.67672400	0.59306500
C	0.78408200	-1.04757800	-0.27901400
C	2.43057700	1.17632700	0.33391900
C	2.15356500	-1.20798500	-0.04376900

C	2.96379600	-0.11459500	0.25881700
H	-3.09861800	0.73757600	-1.00428100
H	-2.15298000	-0.68563800	-1.44930900
H	-1.68776600	2.93576400	-0.21537900
H	-3.03952000	0.06219000	1.37279400
H	-2.05165100	-1.34108300	0.97152900
H	3.06073700	2.02606900	0.56641000
H	-4.52727500	-1.72758500	1.11083300
H	-3.93221400	-2.13447700	-0.35395400
H	4.01735500	-0.29479000	0.43182100
H	0.16057000	-1.90531100	-0.51716900
O	2.75989800	-2.42947500	-0.09802800
H	2.11632800	-3.10539300	-0.32437400

**Anion (N2-H)**	**$C_{10}H_{11}N_2O^-$**

0 2

N	0.29135400	2.46776300	0.12051200
N	-4.00402300	-1.58432100	0.25047500
C	-1.13271100	0.79429900	-0.36633900
C	0.18596000	0.24623400	-0.19044900
C	-2.37857400	0.01759400	-0.65349300
C	1.05117100	1.31763700	0.11127900
C	-1.01478900	2.14246300	-0.16784100
C	-2.85495300	-0.77695200	0.56383400

C	0.70453600	-1.05753300	-0.27803500	
C	2.41595400	1.12060500	0.33319200	
C	2.05502900	-1.24655600	-0.05926900	
C	2.90630400	-0.16670100	0.24697700	
H	-3.18146000	0.68763800	-0.97086600	
H	-2.21027400	-0.68377400	-1.47708500	
H	-1.77455000	2.90785100	-0.21613800	
H	0.63731400	3.39642300	0.28871400	
H	-3.07880800	-0.08749200	1.39333600	
H	-2.03899000	-1.41692700	0.93871600	
H	3.07689900	1.94783800	0.56408700	
H	-4.28993300	-2.05061500	1.11699200	
H	3.95590500	-0.37696100	0.40851900	
H	0.05975900	-1.89753500	-0.52114600	
O	2.65106700	-2.47731100	-0.12474100	
H	1.99672800	-3.13859100	-0.36323900	
**Inter1-O24-HAT**				**$C_{10}H_{12}N_2O + HOO^{\cdot}$**
0 2				
N	-2.19607100	-2.21093500	0.27739800	
N	3.63993400	-1.23644100	0.07408200	
C	-0.07487900	-1.77613300	-0.33231200	
C	-0.79756800	-0.53760300	-0.24121500	
C	1.38407000	-1.88213600	-0.64524000	

C	-2.11529600	-0.83883600	0.14956200
C	-0.96547700	-2.76213300	-0.00840200
C	2.24434100	-1.32893000	0.49620700
C	-0.41944500	0.79517700	-0.45852300
C	-3.06309000	0.16919700	0.34744100
C	-1.34569000	1.79261800	-0.22536000
C	-2.66317500	1.47978600	0.16716200
H	1.67654000	-2.91475100	-0.84759700
H	1.60195800	-1.30691300	-1.55444800
H	-0.81742500	-3.83018400	0.04274800
H	-3.01591700	-2.72735500	0.54464300
H	2.19052700	-2.01268500	1.34653600
H	1.80295400	-0.37834400	0.83158200
H	-4.07977500	-0.05935000	0.64528100
H	4.28476600	-1.00123500	0.81719100
H	3.77758400	-0.62833000	-0.72448400
H	-3.35302700	2.29862300	0.32817900
H	0.57500000	1.03904800	-0.81273300
O	-1.04302200	3.11700700	-0.36989900
H	-0.08459900	3.21850000	-0.38935200
O	1.87484300	2.54133000	0.60177500
O	2.79668800	1.90464600	-0.07268800
H	3.20354500	1.26470800	0.54268100

TS-O24-HAT	$C_{10}H_{12}N_2O + HOO^{\bullet}$		
0 2			
N	-0.82144800	2.82213500	0.50639500
N	-3.52109700	-2.41581400	-0.10600300
C	-1.68368500	0.88912900	-0.26269800
C	-0.24307000	0.81047100	-0.28515900
C	-2.62727000	-0.20337200	-0.65217200
C	0.25721600	2.03891600	0.20670600
C	-1.98611300	2.12671900	0.22377500
C	-2.60119400	-1.37006100	0.33972700
C	0.64929200	-0.17942900	-0.69008100
C	1.63543900	2.29441900	0.32580600
C	2.03167400	0.05749000	-0.56828500
C	2.50386400	1.29876900	-0.04586200
H	-3.64912800	0.17641900	-0.72051800
H	-2.36125100	-0.57920900	-1.64768700
H	-2.94990100	2.58112800	0.39396400
H	-0.77924100	3.76035600	0.86766100
H	-2.94231700	-1.00700200	1.31281800
H	-1.56577600	-1.71522500	0.46861500
H	1.99978200	3.24248000	0.70401800
H	-3.58709000	-3.15412800	0.58520400
H	-3.17907400	-2.84476800	-0.95984100

H	3.57618000	1.42842000	0.02875800
H	0.31401900	-1.12697500	-1.09464700
O	2.91159300	-0.84965300	-0.94389400
H	2.72070500	-1.80583900	-0.44532600
O	2.45074600	-2.72038100	0.43218900
O	1.33005000	-2.23970000	1.04403800
H	1.64487800	-1.90033000	1.89503000

**Inter2-O24-HAT**	**$C_{10}H_{12}N_2O + HOO^{\cdot}$**

0 2

N	-1.10676000	-2.92701900	0.17990500
N	4.09169400	-0.06071400	0.14451900
C	0.72782900	-1.72316800	-0.33563100
C	-0.41163600	-0.83795800	-0.20440700
C	2.12732300	-1.29190600	-0.63436800
C	-1.53702200	-1.64175500	0.12170500
C	0.25964600	-2.97633900	-0.09381100
C	2.74884200	-0.49997600	0.52132000
C	-0.55711100	0.52702600	-0.33933100
C	-2.82905500	-1.09642400	0.32772000
C	-1.85239500	1.12566000	-0.12069900
C	-2.97413000	0.25164500	0.20882800
H	2.75958000	-2.15551800	-0.85128400
H	2.12766700	-0.66216300	-1.53193800

H	0.78078300	-3.92088600	-0.09295000
H	-1.68344400	-3.72589900	0.38811900
H	2.83331300	-1.15883700	1.38959700
H	2.08036300	0.32506800	0.80074500
H	-3.66891300	-1.73631500	0.57353100
H	4.56496300	0.35429100	0.93898800
H	4.03241900	0.66310400	-0.56494600
H	-3.93097900	0.73602900	0.35835100
H	0.27278200	1.17733000	-0.58977500
O	-2.02122100	2.36506500	-0.20573500
H	-0.55345600	3.31488000	-0.46671700
O	0.36346000	3.66587600	-0.51386600
O	0.94760400	3.07740500	0.64518200
H	0.95790000	3.81543600	1.26471100

**TS- C7-RAF**	**$C_{10}H_{12}N_2O$ + HOO$^{\bullet}$**

0 2

N	0.17978400	-1.79211000	1.01745400
N	2.97877200	3.33013600	0.10257400
C	0.97795900	0.11959500	0.08009400
C	-0.44035100	0.16968500	0.06377300
C	1.93182100	1.16891800	-0.37809300
C	-0.91210800	-1.04708000	0.61234800
C	1.32241000	-1.15503800	0.56765700

C	2.02739300	2.34147400	0.60450400
C	-1.36057400	1.13781200	-0.38628500
C	-2.27477000	-1.32585300	0.69805800
C	-2.70627100	0.85489400	-0.29309400
C	-3.16155700	-0.36900300	0.24245400
H	2.92775400	0.74244100	-0.51661100
H	1.61118700	1.55015200	-1.35579700
H	0.15743000	-2.79940700	1.08978000
H	2.39653500	1.96807400	1.56308300
H	1.01906300	2.74134200	0.78346400
H	-2.63413700	-2.26124300	1.10936400
H	3.11460400	4.07789600	0.77280100
H	2.63644900	3.75385700	-0.75333600
H	-4.23073700	-0.53454400	0.28507400
H	-1.01611100	2.07816000	-0.80621900
O	-3.68074000	1.72044300	-0.70516300
H	-3.27705600	2.50791800	-1.07856600
H	2.28817200	-1.45133600	0.95013100
O	1.65804500	-2.13115300	-1.11030800
O	1.74508500	-3.44972100	-0.69449700
H	2.69412100	-3.62847600	-0.67211500

**Product-C7-RAF**	**$C_{10}H_{12}N_2OOOH$**
0 2	

N	0.28011900	-1.98703100	0.52526600
N	2.68049000	3.45425800	0.27054300
C	0.81920700	0.19156000	-0.14467100
C	-0.57214100	0.06428200	-0.09960200
C	1.62818500	1.38353300	-0.51484300
C	-0.89373800	-1.24691600	0.35160700
C	1.41992200	-1.11604600	0.29049200
C	1.86303000	2.31656000	0.68294800
C	-1.61055400	0.97616200	-0.39036300
C	-2.20598200	-1.63652500	0.54065200
C	-2.91824500	0.57119500	-0.20026700
C	-3.22010700	-0.71829300	0.26853000
H	2.59775300	1.07780200	-0.91504100
H	1.11542200	1.94615000	-1.30393700
H	0.32992500	-2.60264300	1.32376700
H	2.40915400	1.76986100	1.45651300
H	0.88886800	2.59537700	1.10951300
H	-2.44928800	-2.63522800	0.88300200
H	2.91499400	4.04137300	1.06241700
H	2.17812600	4.03354300	-0.39371000
H	-4.26150600	-0.98176100	0.40151300
H	-1.38258500	1.97690600	-0.74439000
O	-3.98694900	1.38583200	-0.44787000
H	-3.67778600	2.23324500	-0.77772300

H	2.06755000	-1.05481200	1.17496400
O	2.22605000	-1.57881600	-0.78660600
O	2.82155700	-2.79905200	-0.36854300
H	2.20238100	-3.44719300	-0.73030000

**TS-C9-RAF**	**$C_{10}H_{12}N_2O$ + $HOO^{\cdot}$**

0 2

N	0.24549800	2.79058300	0.37295400
N	4.04211400	-1.63583800	-0.66095600
C	1.37713600	0.84817100	0.37699300
C	0.02659000	0.60269600	-0.01520400
C	2.46382600	-0.17278900	0.50403500
C	-0.65827600	1.82537600	-0.00158500
C	1.46550300	2.20032300	0.60196000
C	2.99700800	-0.63244900	-0.85441600
C	-0.68056900	-0.59995200	-0.30951000
C	-2.01590900	1.93464700	-0.34466800
C	-2.03681800	-0.45758100	-0.71096300
C	-2.69216700	0.77857000	-0.69450900
H	3.29747300	0.22386300	1.08812600
H	2.07237100	-1.03985400	1.04866200
H	2.31241500	2.78789000	0.92120300
H	0.04473900	3.77092100	0.47553700
H	3.43644000	0.22530800	-1.37041800

H	2.15685200	-0.98054600	-1.47294000
H	-2.52451700	2.89120700	-0.33778000
H	4.44392800	-1.91897300	-1.54710500
H	3.65701700	-2.46891000	-0.22800400
H	-3.73801000	0.80435400	-0.97248400
H	-0.13857400	-1.48038600	-0.63783500
O	-2.74970200	-1.54992200	-1.07414300
H	-2.29023900	-2.33557200	-0.74790100
O	-0.87253300	-1.39579200	1.42580600
O	-1.59805500	-2.57470000	1.25409800
H	-2.45881100	-2.36128400	1.63807000

**Product-C9-RAF**	**$C_{10}H_{12}N_2OOOH$**

0 2

N	0.01735700	2.83535700	0.25303300
N	4.06085400	-1.36920700	-0.66195800
C	1.26641500	0.97857500	0.35585800
C	-0.07934600	0.62465400	0.06555100
C	2.43542300	0.05185300	0.49174900
C	-0.83734500	1.78949500	0.00960400
C	1.28565000	2.35235200	0.46304500
C	2.93393700	-0.45937200	-0.86130500
C	-0.70316400	-0.71010100	-0.11429500
C	-2.22306900	1.81855200	-0.27837400

C	-2.16032200	-0.59873700	-0.47389300
C	-2.84920000	0.58950700	-0.52414300
H	3.26382800	0.55295800	0.99801700
H	2.14603100	-0.80364900	1.11139900
H	2.10718400	3.01568500	0.68264100
H	-0.24580600	3.80641800	0.26602900
H	3.27966900	0.39092100	-1.45498000
H	2.09393200	-0.91105100	-1.40872500
H	-2.77805800	2.74634200	-0.31505500
H	4.44215200	-1.67545300	-1.54954500
H	3.75781400	-2.20076800	-0.16545700
H	-3.90285700	0.54699900	-0.77191700
H	-0.17847100	-1.32628700	-0.85975300
O	-2.80612900	-1.74954700	-0.78257300
H	-2.26505900	-2.49325300	-0.48802000
O	-0.51851900	-1.40547600	1.15072800
O	-0.85129700	-2.78555500	0.91461900
H	-1.27415200	-3.00795400	1.75285700

**TS-C10-RAF**	**$C_{10}H_{12}N_2O + HOO^{\cdot}$**

0 2

N	-0.25696400	-2.13490800	0.56558200
N	4.92999700	0.61011900	-0.19318700
C	1.46467400	-0.92308600	-0.22296000

C	0.34158500	-0.05338000	0.01634700
C	2.81836100	-0.51678800	-0.71346200
C	-0.70296700	-0.84831900	0.51275600
C	1.04854400	-2.17990500	0.11847000
C	3.62342300	0.23786400	0.34664700
C	0.12737200	1.32115500	-0.17805500
C	-2.00889200	-0.33778200	0.76408300
C	-1.12252100	1.85892600	0.14614900
C	-2.14729600	1.07096600	0.65051600
H	3.39063700	-1.39358500	-1.02516900
H	2.71226300	0.12073200	-1.60018500
H	1.58613600	-3.11458100	0.07990000
H	-0.83044900	-2.93290900	0.78495500
H	3.78231500	-0.42422700	1.20148500
H	3.03193400	1.09092900	0.70946100
H	5.50892500	1.04140200	0.51787600
H	4.82763400	1.28442000	-0.94433400
H	-3.08162000	1.54602400	0.92135500
H	0.91293000	1.95787000	-0.57473500
O	-1.38765100	3.19292200	0.00026300
H	-0.59225200	3.65351300	-0.27771400
H	-2.68178600	-0.87080300	1.42494100
O	-2.86812000	-1.08701800	-0.73416800
O	-4.20278100	-0.74373700	-0.67038300

H	-4.26318300	0.03949000	-1.23324000

Product-C10-RAF	$C_{10}H_{12}N_2OOOH$

0 2

N	-0.30051400	-2.13767600	0.27798800
N	4.95572100	0.54536500	0.10698700
C	1.48751200	-0.92251000	-0.30851400
C	0.35990700	-0.04956200	-0.11356900
C	2.88166700	-0.50898500	-0.66075600
C	-0.71933200	-0.84649900	0.24862800
C	1.03519800	-2.19200400	-0.06551600
C	3.60307100	0.16354800	0.50925700
C	0.19091400	1.34951200	-0.25040200
C	-2.09289400	-0.35702200	0.51487700
C	-1.09423200	1.89585100	0.01281400
C	-2.16133200	1.13190600	0.38173100
H	3.46797400	-1.37397700	-0.97928100
H	2.85739500	0.18498200	-1.51016000
H	1.55788200	-3.13409300	-0.11287400
H	-0.90487700	-2.93144300	0.41983400
H	3.67964000	-0.55345400	1.33049300
H	2.99291100	1.00269000	0.87384800
H	5.47750700	0.91825200	0.89152000
H	4.92650300	1.26955100	-0.60298300

H	-3.09814000	1.62211000	0.61421000
H	1.00981400	1.99030000	-0.55953400
O	-1.29254000	3.24606300	-0.09365300
H	-0.45708800	3.68187600	-0.27696600
H	-2.45445200	-0.67219100	1.50345700
O	-2.93114900	-1.05380900	-0.44222900
O	-4.28321300	-0.78074000	-0.10390500
H	-4.52918400	-0.13595900	-0.77939700

**TS-C11-RAF**	**$C_{10}H_{12}N_2O$ + $HOO^{\cdot}$**

0 2

N	0.56052800	2.63921300	0.01339100
N	4.26298800	-1.98794400	-0.08625200
C	1.67353100	0.72461900	0.42444200
C	0.33063700	0.40943500	-0.01092800
C	2.73777500	-0.26899900	0.76396900
C	-0.32801400	1.64058200	-0.26270300
C	1.76250500	2.08381100	0.42654200
C	3.29706800	-0.96443200	-0.48016200
C	-0.34329900	-0.78302000	-0.21934800
C	-1.64574000	1.70322400	-0.76155200
C	-1.73373000	-0.73625500	-0.54236200
C	-2.31416300	0.52607400	-0.93807800
H	3.55424500	0.23102200	1.29524000

H	2.34150000	-1.03507500	1.43712900
H	2.59150700	2.71798000	0.70057800
H	0.37366800	3.62469700	-0.06540800
H	3.70579500	-0.20013800	-1.15605800
H	2.47943800	-1.45615800	-1.01373900
H	-2.10223600	2.65149300	-1.02048200
H	4.57822800	-2.51896000	-0.88965000
H	-3.32108500	0.49380700	-1.33448900
H	0.12480200	-1.74858900	-0.06428400
O	-2.34511700	-1.84082500	-1.07930500
H	-2.45278600	-2.48849200	-0.37302200
H	5.08454900	-1.56193300	0.32964100
O	-2.34411300	-0.90710200	1.22504600
O	-3.73294900	-0.91238300	1.21106900
H	-3.96422400	0.00371100	1.41116800

**Product-C11-RAF**	**$C_{10}H_{12}N_2OOOH$**

0 2

N	-0.74529900	2.75052500	-0.06069400
N	-3.89591100	-2.25446900	0.29471700
C	-1.68744300	0.73776400	-0.41114200
C	-0.27381200	0.55935300	-0.14323500
C	-2.67252600	-0.35840600	-0.66086400
C	0.26900500	1.84870500	0.06651300

C	-1.92378200	2.07878300	-0.35039900	
C	-2.99320200	-1.14932700	0.61013500	
C	0.53531700	-0.56119200	-0.09349400	
C	1.65396100	2.06157200	0.33505500	
C	1.99711700	-0.42159500	0.20498000	
C	2.47432900	0.99419800	0.38648700	
H	-3.59865200	0.06259100	-1.06597300	
H	-2.28443100	-1.05541000	-1.40972800	
H	-2.84317100	2.62422300	-0.49625800	
H	-0.65065300	3.74968500	0.01282100	
H	-3.38434000	-0.45467800	1.36696500	
H	-2.06704900	-1.57066900	1.01015800	
H	2.03676900	3.06316300	0.49742800	
H	-4.05494600	-2.83864700	1.10722800	
H	3.53229100	1.09376900	0.59443100	
H	0.17271500	-1.55904200	-0.30749000	
O	2.36902800	-1.20594500	1.34416700	
H	1.63462300	-1.19805300	1.96454800	
H	-4.80062800	-1.90240800	-0.00017900	
O	2.81610600	-0.93097400	-0.84411600	
O	2.51429800	-2.30310500	-1.03940700	
H	2.92670600	-2.70585700	-0.26149300	
**TS-C12-RAF**				**$C_{10}H_{12}N_2O + HOO^{\bullet}$**

0 2			
N	0.16981700	2.42461200	-0.41123300
N	4.80835300	-1.18794200	0.20903500
C	1.62162200	0.86000800	0.31299800
C	0.38762100	0.22060700	-0.01159700
C	2.87448500	0.19682700	0.79334800
C	-0.51255300	1.22413800	-0.45797800
C	1.44137600	2.19792700	0.05428000
C	3.59984200	-0.55957200	-0.32177100
C	-0.03231500	-1.13054100	0.01218200
C	-1.80061200	0.93568700	-0.86626900
C	-1.31264200	-1.42626200	-0.36729400
C	-2.26621800	-0.39333600	-0.70262400
H	3.56145500	0.93408000	1.21549800
H	2.63157700	-0.50486600	1.60069700
H	2.13862700	3.01320900	0.17548100
H	-0.21182500	3.32258000	-0.65373300
H	3.89678300	0.15551200	-1.09317100
H	2.90070300	-1.26518100	-0.79314800
H	-2.47368500	1.69783300	-1.23944600
H	5.34390000	-1.62910700	-0.52946900
H	4.56971300	-1.91281600	0.87766700
H	-3.14746300	-0.71145300	-1.24554500

H	0.65511400	-1.91847700	0.30492200
O	-1.82133300	-2.68267900	-0.40684000
H	-1.18201600	-3.30611600	-0.05095700
O	-3.17955500	-0.40450100	0.93025400
O	-4.20959200	0.51424500	0.83936500
H	-3.84987800	1.29869300	1.27269600

**Product-C12-RAF**	**$C_{10}H_{12}N_2OOOH$**

0 2

N	-0.27847900	2.46959000	0.24206200
N	-4.82048100	-1.29499500	0.03499600
C	-1.70744100	0.83130100	-0.35216100
C	-0.44430600	0.25151200	-0.09391900
C	-2.96750000	0.11501000	-0.72784000
C	0.45570300	1.29455300	0.27388200
C	-1.56624300	2.19119900	-0.14178400
C	-3.59584900	-0.61832300	0.45917000
C	0.02746600	-1.10229500	-0.11482700
C	1.76696900	1.08507100	0.60286300
C	1.32264300	-1.35615000	0.16277400
C	2.33977400	-0.28480900	0.44490800
H	-3.70236600	0.81643500	-1.12993600
H	-2.75583200	-0.60957600	-1.52350500
H	-2.29912900	2.97650300	-0.24528100

H	0.09810000	3.38846900	0.39941700
H	-3.85831300	0.11618100	1.22482300
H	-2.84706500	-1.28929200	0.90434300
H	2.44061700	1.88300800	0.88641500
H	-5.29032500	-1.72597900	0.82260400
H	-4.60871200	-2.03419900	-0.62695900
H	2.93084000	-0.58214400	1.32065800
H	-0.65911700	-1.91789800	-0.32412900
O	1.87788800	-2.59589400	0.21829700
H	1.22410700	-3.25564000	-0.03083000
O	3.24768900	-0.34384500	-0.68710800
O	4.39570900	0.41990800	-0.33738800
H	5.06232600	-0.27180100	-0.24582500

Table S9 Optimized structures of 11-OH-TA forms in water using M06-2X/6-311++G(d,p)

**Stable Cationic form**			**$[C_{10}H_{13}N_2O]^+$**
1 1			
N	0.52196000	2.48214100	0.10805700
N	-4.02673300	-1.34059000	0.25213700
C	-0.99641300	0.88169000	-0.33065400
C	0.29129700	0.26168000	-0.17140200
C	-2.28927500	0.18594000	-0.62123900
C	1.21749200	1.29386300	0.09951500

C	-0.80060700	2.22659600	-0.15153500
C	-2.76831000	-0.60464400	0.58833300
C	0.73715200	-1.07077600	-0.24746400
C	2.57544500	1.03066800	0.29727200
C	2.08124900	-1.31975200	-0.05325600
C	2.99691800	-0.28360700	0.21900200
H	-3.05186700	0.91867000	-0.89846600
H	-2.16489300	-0.50089600	-1.46569400
H	-1.51694900	3.03354700	-0.19657600
H	0.92263800	3.39630200	0.25877000
H	-2.99574000	0.04918000	1.42824100
H	-2.03965400	-1.35136600	0.89966200
H	3.27811600	1.82948100	0.50327300
H	-4.37236800	-1.87440100	1.05330500
H	-3.86975700	-1.99445100	-0.52026000
H	4.04004900	-0.53873700	0.36385900
H	0.05076000	-1.88653400	-0.45382300
O	2.60562200	-2.59639100	-0.11263700
H	1.90149500	-3.22036700	-0.32735700
H	-4.76308300	-0.69011300	-0.03662000

**Cation radical**	**$[C_{10}H_{13}N_2O]^{\bullet+}$**

2 2

N	0.56424100	2.42025200	0.10253000
N	-4.07801000	-1.28047800	0.26116300

C	-1.00980200	0.86780900	-0.33618600
C	0.28677900	0.22401400	-0.17431400
C	-2.30447600	0.18374100	-0.62827200
C	1.23377000	1.26748100	0.09953100
C	-0.79305300	2.19293600	-0.16067600
C	-2.80220500	-0.57836600	0.59334100
C	0.71466200	-1.07233900	-0.25055400
C	2.61703300	1.01476200	0.30557900
C	2.10414200	-1.32121100	-0.05079900
C	3.03789500	-0.27683600	0.22962000
H	-3.04916400	0.92582300	-0.92476600
H	-2.17010200	-0.51530800	-1.45980500
H	-1.47926400	3.02439300	-0.20211800
H	0.97737800	3.33391000	0.25988200
H	-3.01238600	0.09369500	1.42292500
H	-2.09369600	-1.33990800	0.91449300
H	3.29985100	1.82928100	0.51196300
H	-4.44206300	-1.78912300	1.07096400
H	-3.93832200	-1.95347600	-0.49828000
H	4.07523500	-0.55048500	0.37208500
H	0.04736400	-1.90253500	-0.45865500
O	2.59483600	-2.54180500	-0.11937400
H	1.90887300	-3.19846600	-0.32207000
H	-4.79448200	-0.61372100	-0.04110100

Radical Form (O24-H)				$[C_{10}H_{12}N_2O]^{+\bullet}$
1 2				
N	0.62975000	2.39378200	0.11406400	
N	-4.06618200	-1.23811900	0.25766700	
C	-0.96137000	0.86630200	-0.33449200	
C	0.31503600	0.19653600	-0.18146800	
C	-2.27447100	0.21327100	-0.62473100	
C	1.27874000	1.21049900	0.09826600	
C	-0.71973900	2.19190800	-0.14632900	
C	-2.77461500	-0.56147000	0.58706700	
C	0.71486600	-1.12000300	-0.26614500	
C	2.65242300	0.92467000	0.30083600	
C	2.10510300	-1.45184700	-0.06604000	
C	3.04615500	-0.37504400	0.21997500	
H	-3.01026600	0.97331000	-0.89893700	
H	-2.16868200	-0.47446500	-1.47020100	
H	-1.39778200	3.03094600	-0.17917700	
H	1.06111500	3.29447200	0.27817500	
H	-2.96542100	0.09848700	1.43110100	
H	-2.07645200	-1.34065300	0.88799000	
H	3.35288500	1.72437800	0.51101300	
H	-4.42786100	-1.75916800	1.06036100	
H	-3.94546300	-1.89581500	-0.51804400	
H	4.08229200	-0.65719200	0.36544700	

H	0.01956300	-1.92473000	-0.48069600	
O	2.50173800	-2.64676100	-0.13616700	
H	-4.77557100	-0.55405400	-0.02145400	
**Anion (O24-H)**				**[$C_{10}H_{12}N_2O$]**
2 1				
N	0.67524100	2.32784700	0.10923600	
N	-4.11709500	-1.18292100	0.26425500	
C	-0.98106200	0.85555700	-0.33627500	
C	0.30003000	0.15530200	-0.18660000	
C	-2.29938300	0.22029600	-0.62533400	
C	1.29833200	1.18455800	0.09685000	
C	-0.71456200	2.15937800	-0.15183300	
C	-2.80886900	-0.54300300	0.59131200	
C	0.68257400	-1.12446900	-0.27483200	
C	2.70212000	0.92048900	0.30643500	
C	2.13029700	-1.45197600	-0.06570800	
C	3.09741700	-0.35519900	0.22642200	
H	-3.01814000	0.99447200	-0.90164200	
H	-2.19296100	-0.46829500	-1.46909700	
H	-1.35574100	3.02618600	-0.17796000	
H	1.12105800	3.22880100	0.27552200	
H	-2.97793200	0.12221700	1.43553200	
H	-2.12868100	-1.33969700	0.88686900	
H	3.37535200	1.74257000	0.51519100	

H	-4.49108700	-1.69001100	1.07064800
H	-4.01673600	-1.84763000	-0.50863900
H	4.12808700	-0.65636800	0.36759600
H	0.00893200	-1.94704700	-0.49068000
O	2.50094800	-2.60315000	-0.13561700
H	-4.80811900	-0.48006600	-0.01546000

**Radical Form (N1-H)**	**$[C_{10}H_{12}N_2O]^{+\bullet}$**

1 2

N	0.44925000	2.56193200	0.12258800
N	-4.03562100	-1.32265000	0.27166100
C	-0.99529500	0.83766700	-0.35277100
C	0.30728600	0.25553400	-0.18370300
C	-2.27476700	0.14268200	-0.64379500
C	1.16504000	1.34005600	0.10467100
C	-0.79501100	2.24650700	-0.14386700
C	-2.75590900	-0.62173000	0.58774700
C	0.78890000	-1.04711100	-0.26394900
C	2.51165500	1.13478200	0.31873200
C	2.15779100	-1.24258200	-0.04718500
C	3.00532400	-0.17452900	0.24091100
H	-3.03398800	0.86954100	-0.94190500
H	-2.12722700	-0.56630900	-1.46570700
H	-1.57683900	2.99397400	-0.19910200

H	-2.95446600	0.04915500	1.42101100
H	-2.04011200	-1.38179200	0.89466300
H	3.17872700	1.95990500	0.53997000
H	-4.38531500	-1.83614000	1.08487600
H	-3.90724600	-1.99150600	-0.49346800
H	4.05773400	-0.37590000	0.40181100
H	0.14150400	-1.88938000	-0.48869600
O	2.72150500	-2.49175200	-0.10822800
H	2.04743200	-3.14522200	-0.33308100
H	-4.75772800	-0.65494500	-0.01502800

**Anion (N1-H)**	**[$C_{10}H_{12}N_2O$]**

2 1

N	0.63911900	2.45589500	0.11762200
N	-4.10313500	-1.23826200	0.25733300
C	-1.02234100	0.88786800	-0.33007600
C	0.28545700	0.21638600	-0.17072500
C	-2.32137700	0.21893300	-0.62296400
C	1.22810400	1.31371100	0.10605400
C	-0.75776100	2.19130700	-0.15485100
C	-2.81908800	-0.55439000	0.59262500
C	0.70536200	-1.05061000	-0.24971300
C	2.64039700	1.05155600	0.31446700
C	2.13624500	-1.27404600	-0.05024300

C	3.07725100	-0.22048700	0.23689100
H	-3.05869200	0.97331200	-0.90579000
H	-2.19399300	-0.47096700	-1.46354200
H	-1.44269600	3.02473400	-0.20079000
H	-3.01808900	0.10989300	1.43103700
H	-2.11646600	-1.32677900	0.90040200
H	3.30926200	1.87833000	0.52232300
H	-4.46790700	-1.75319900	1.06292400
H	-3.97470800	-1.90299400	-0.51133300
H	4.11230500	-0.50340400	0.37363900
H	0.06802500	-1.90408700	-0.45799200
O	2.62161300	-2.45348200	-0.13033200
H	1.95881100	-3.14195100	-0.32958900
H	-4.81414400	-0.55983600	-0.03211400
**Radical Form (N2-H)**		**$[C_{10}H_{12}N_2O]^{+\bullet}$**	
1 2			
N	0.29034400	2.45850500	0.10913300
N	-4.03153900	-1.46772600	0.26743500
C	-1.06265700	0.73616200	-0.39305600
C	0.25787900	0.21705600	-0.20800600
C	-2.30085800	-0.01606900	-0.67964200
C	1.09609800	1.30157500	0.10402900
C	-0.95856800	2.14800700	-0.17379000

C	-2.81110300	-0.72712900	0.58628600
C	0.77843500	-1.07475800	-0.28939500
C	2.44154100	1.15252300	0.34225000
C	2.14613100	-1.23611600	-0.04859000
C	2.96023200	-0.14522800	0.26102800
H	-3.07241300	0.66385700	-1.04805600
H	-2.09816400	-0.77219700	-1.44319700
H	-1.74083600	2.89176200	-0.22316800
H	0.62695500	3.39935500	0.29690400
H	-2.95159400	0.01897400	1.37630100
H	-2.05065600	-1.43162700	0.92729600
H	3.07916900	1.99397200	0.58226800
H	-4.33560100	-1.96781600	1.09784900
H	4.01424900	-0.32023400	0.43924900
H	0.15226400	-1.92687500	-0.53301900
O	2.74480600	-2.46000100	-0.10559900
H	2.09480200	-3.13900500	-0.32828300
H	-4.77103000	-0.80173900	0.05798300

**Anion (N2-H)**	**[$C_{10}H_{12}N_2O$]**

2 1

N	0.51276700	2.36022900	0.10389700
N	-4.11127300	-1.36196200	0.23620400
C	-1.09453500	0.82555700	-0.35299700

C	0.22129700	0.17829400	-0.18232700
C	-2.37061700	0.11607700	-0.63892900
C	1.17833600	1.25553700	0.10794400
C	-0.87921500	2.13690300	-0.17467400
C	-2.87037100	-0.66523900	0.57910900
C	0.65637400	-1.08033600	-0.26770100
C	2.59654100	1.03969900	0.33366200
C	2.10037100	-1.29494900	-0.05645300
C	3.03916800	-0.22103800	0.25008900
H	-3.12377900	0.84809900	-0.93916000
H	-2.21622900	-0.57729800	-1.47076600
H	-1.54368400	2.98526000	-0.21281400
H	0.92118700	3.28306600	0.26848400
H	-2.98476000	0.03107100	1.41765100
H	-2.12441400	-1.40798000	0.86899400
H	3.24088300	1.88180900	0.55388900
H	-4.42124800	-1.89660600	1.04230500
H	4.07424100	-0.50099500	0.39506300
H	0.02883900	-1.93837600	-0.48845400
O	2.59483400	-2.45103200	-0.14025300
H	1.95200400	-3.16182200	-0.34778600
H	-4.83626100	-0.66887600	0.06757800

**Inter1-O24-HAT**	**$[C_{10}H_{13}N_2O]^+ + HOO^\bullet$**

1 2

N	0.02562500	3.03947800	0.46871300
N	-3.85305300	-1.41697100	-0.10642400
C	-1.24999000	1.34676400	-0.28412900
C	0.11876500	0.91289800	-0.25346100
C	-2.42948100	0.52118000	-0.69238200
C	0.88947100	1.99651200	0.22208500
C	-1.25192000	2.64216600	0.16546900
C	-2.71023200	-0.56154600	0.34089100
C	0.75479500	-0.29347900	-0.60168300
C	2.27662500	1.90430800	0.36658900
C	2.12579000	-0.38185000	-0.43857700
C	2.88436700	0.70678200	0.03864100
H	-3.31175900	1.15771600	-0.79901200
H	-2.24136700	0.04549000	-1.66137100
H	-2.08194700	3.32159700	0.29036900
H	0.28726500	3.95139900	0.81404900
H	-2.99036900	-0.13247000	1.30119800
H	-1.85567900	-1.22317300	0.47760800
H	2.85965300	2.74221600	0.73054100
H	-4.06836200	-2.14132000	0.58348500
H	-3.63658300	-1.87954700	-0.99387900
H	3.95557400	0.58356200	0.14531200
H	0.19351900	-1.13173200	-1.00702000

O	2.82163800	-1.53450500	-0.74177100
H	2.19569600	-2.27183000	-0.81270000
O	0.86457000	-3.54447600	0.10245400
O	0.45754900	-2.95829000	1.18900400
H	0.58318500	-1.99096100	1.06458800
H	-4.69603000	-0.85273100	-0.24734000
**TS-O24-HAT**		**$[C_{10}H_{13}N_2O]^+$ + $HOO^{\bullet}$**	
1 2			
N	-0.69725700	2.77168800	0.56971100
N	-3.70286000	-2.30355600	-0.06707500
C	-1.67859300	0.90700500	-0.21804200
C	-0.24663600	0.76420400	-0.31799200
C	-2.70352400	-0.10995700	-0.60878000
C	0.32670500	1.95914900	0.18966000
C	-1.90018900	2.13972000	0.32636700
C	-2.69239900	-1.28671200	0.35753900
C	0.58697100	-0.24626400	-0.78927100
C	1.71723300	2.16198100	0.24662300
C	1.97701400	-0.04994400	-0.73752500
C	2.52791600	1.15094500	-0.21007200
H	-3.69543600	0.34895700	-0.61090300
H	-2.50168200	-0.47860800	-1.62003800
H	-2.83221400	2.63111700	0.56125500

H	-0.59526800	3.69601500	0.96632300
H	-2.95611900	-0.97687200	1.36697700
H	-1.72562700	-1.78766600	0.37495500
H	2.12873100	3.08347100	0.64070300
H	-3.70758300	-3.10977100	0.56268500
H	-3.50437500	-2.64180400	-1.01322800
H	3.60669000	1.24848200	-0.18841600
H	0.19668100	-1.17597200	-1.18961900
O	2.80478400	-1.00012200	-1.20274900
H	2.84782300	-1.79497900	-0.49270400
O	2.74058000	-2.54846800	0.63427600
O	1.76402600	-1.89085400	1.30494800
H	2.22111300	-1.24478400	1.87290900
H	-4.64402500	-1.89972700	-0.07218500

**Inter2-O24-HAT**	**$[C_{10}H_{13}N_2O]^+ + HOO^{\bullet}$**

1 2

N	-1.34780100	2.82709100	0.25993400
N	-2.93416800	-2.90187300	0.09253800
C	-1.76111100	0.68736500	-0.30498300
C	-0.32893000	0.89006400	-0.20507100
C	-2.44641600	-0.59667200	-0.64549700
C	-0.13106000	2.25718900	0.15378700
C	-2.33425100	1.88559000	-0.01387600

C	-2.30504200	-1.60469300	0.48744800
C	0.75976000	0.06886100	-0.39177500
C	1.15808900	2.82040800	0.33421300
C	2.08504600	0.60561400	-0.20614000
C	2.23389800	2.00707800	0.15611200
H	-3.50562500	-0.40986500	-0.83799600
H	-2.00759700	-1.02083600	-1.55474000
H	-3.37576000	2.16580300	0.02094200
H	-1.51993800	3.79633100	0.49679600
H	-2.81074900	-1.26534400	1.38954400
H	-1.26164000	-1.81688400	0.71584800
H	1.26724300	3.86410000	0.60436100
H	-2.85298300	-3.59469100	0.84087800
H	-2.48387400	-3.28613500	-0.74345700
H	3.24474900	2.37608300	0.28208200
H	0.65712600	-0.97344500	-0.67475700
O	3.10831200	-0.12520300	-0.35263100
H	2.86207100	-1.80147300	-0.49587100
O	2.67084800	-2.76935800	-0.42645900
O	1.91231900	-2.83139100	0.77629000
H	2.57790500	-3.04962400	1.44361900
H	-3.92888400	-2.77776900	-0.11745900

**TS- C7-RAF**	**$[C_{10}H_{13}N_2O]^+ + HOO^{\cdot}$**

1 2

N	0.00215200	1.84880300	-1.03472400
N	3.06578200	-3.14551100	-0.11583100
C	0.93435200	-0.00960600	-0.14026300
C	-0.47437000	-0.15004300	-0.08391400
C	1.96796800	-0.97795800	0.32456700
C	-1.03436900	1.03349300	-0.62967100
C	1.18936100	1.28571100	-0.64864400
C	2.04182100	-2.17777100	-0.61287900
C	-1.31973800	-1.17284700	0.39719900
C	-2.41345100	1.22336300	-0.70171700
C	-2.68039000	-0.97301000	0.31988600
C	-3.22610800	0.21131000	-0.22450300
H	2.94296000	-0.48592000	0.36447400
H	1.72261600	-1.33007800	1.33285600
H	-0.09837200	2.80849800	-1.33444400
H	2.34081200	-1.88306300	-1.61683800
H	1.09392600	-2.71095000	-0.65928400
H	-2.83616600	2.13062400	-1.11557200
H	3.12549800	-3.96671500	-0.72334100
H	2.83787900	-3.46652500	0.82982200
H	-4.30462300	0.31148000	-0.25733900
H	-0.91528100	-2.08638800	0.82133700

O	-3.59140100	-1.90335600	0.76760600
H	-3.12370300	-2.65906100	1.14445500
H	2.12233100	1.65598400	-1.04637800
O	1.60058800	2.14899800	1.12004700
O	1.63629600	3.50842000	0.84074400
H	2.55030600	3.68005100	0.56852300
H	3.99196100	-2.70932300	-0.08193500

**Product-C7-RAF**	**$[C_{10}H_{13}N_2O]^+OOH$**

1 2

N	0.17386900	-2.02269100	0.49151800
N	2.76578000	3.29888800	0.34896300
C	0.76253200	0.13161500	-0.20391000
C	-0.63051700	0.04811900	-0.14120500
C	1.59623900	1.31356700	-0.55445300
C	-0.97782800	-1.24944900	0.33399500
C	1.33065800	-1.17839500	0.26137300
C	1.87591100	2.14805600	0.69326800
C	-1.64287300	0.99296800	-0.42032600
C	-2.29784900	-1.59556200	0.56274200
C	-2.95499700	0.62572200	-0.19552300
C	-3.28782600	-0.64823200	0.29855900
H	2.54722700	0.99089400	-0.98879100
H	1.07898300	1.93429300	-1.29210600

H	0.21008400	-2.63790700	1.29449700
H	2.39008700	1.56653500	1.45630000
H	0.95979700	2.56354200	1.10882100
H	-2.56159100	-2.57810600	0.93547300
H	2.97728100	3.86518500	1.17439000
H	2.32485200	3.90609100	-0.34797700
H	-4.33221400	-0.88324300	0.46541700
H	-1.39822600	1.98471500	-0.78705200
O	-4.00693300	1.48248800	-0.43267700
H	-3.67018800	2.31932000	-0.77647600
H	1.96699300	-1.10732300	1.15019200
O	2.15691000	-1.66896600	-0.80178100
O	2.88618500	-2.79002200	-0.31927800
H	2.34794800	-3.54235000	-0.60897000
H	3.65142900	2.96651800	-0.04432600

**TS-C9-RAF**	**$[C_{10}H_{13}N_2O]^+$ + HOO$^\cdot$**

1 2

N	0.13425200	2.79942900	0.36333900
N	4.01105200	-1.55799000	-0.68886400
C	1.32074800	0.89415000	0.39422000
C	-0.01716100	0.59915000	-0.00566200
C	2.45119300	-0.07801000	0.52616600
C	-0.73449200	1.80522500	-0.00936700

C	1.36593700	2.25475700	0.60334700
C	2.91833500	-0.54939800	-0.84381200
C	-0.69296500	-0.62576200	-0.28504200
C	-2.09027500	1.87655000	-0.37447600
C	-2.03426800	-0.51355600	-0.74980000
C	-2.72570200	0.70389100	-0.74705700
H	3.28573500	0.39200300	1.05266700
H	2.13268600	-0.94815200	1.11009000
H	2.19392100	2.87482900	0.91189200
H	-0.09716900	3.78014700	0.44252900
H	3.32194300	0.27157300	-1.43354400
H	2.11651500	-1.03315000	-1.39933700
H	-2.61967000	2.82159800	-0.37609900
H	4.33653100	-1.89923200	-1.59692000
H	3.68688200	-2.36310600	-0.14531300
H	-3.76314900	0.71007900	-1.05790800
H	-0.13104200	-1.51721100	-0.54555400
O	-2.71503200	-1.62094400	-1.14376600
H	-2.15290500	-2.40435300	-1.06609700
O	-1.01521300	-1.29389200	1.50139400
O	-1.54519900	-2.58147200	1.40323700
H	-2.50484300	-2.45388600	1.44145700
H	4.81288500	-1.15273300	-0.19722000

Product-C9-RAF	$[C_{10}H_{13}N_2O]^+OOH$

1 2

N	-0.00837400	2.82985400	0.26176400
N	4.02584300	-1.38973900	-0.68342300
C	1.24402900	0.97799000	0.36314300
C	-0.09794600	0.61391400	0.06641700
C	2.42351500	0.06488800	0.50365200
C	-0.85662700	1.78134000	0.01243300
C	1.25712900	2.35438900	0.47201600
C	2.87352800	-0.45193100	-0.85564700
C	-0.72578800	-0.72269900	-0.08737000
C	-2.23823500	1.80919500	-0.29666000
C	-2.15165000	-0.60300800	-0.54739900
C	-2.84929600	0.58197300	-0.59063200
H	3.25094300	0.59993800	0.97704400
H	2.16842400	-0.78880100	1.13975300
H	2.07576100	3.02300200	0.68887000
H	-0.27602000	3.80446600	0.27018800
H	3.21338300	0.35733200	-1.49932700
H	2.08477300	-1.00839200	-1.35928000
H	-2.79192300	2.73794500	-0.33037100
H	4.34972100	-1.75429500	-1.58295200
H	3.75981200	-2.18598200	-0.09707100

H	-3.89214600	0.54901100	-0.88518900
H	-0.16236600	-1.39420600	-0.74694300
O	-2.78688700	-1.75013300	-0.93305500
H	-2.23191600	-2.51601800	-0.73067700
O	-0.65754600	-1.33378200	1.24401800
O	-1.03732900	-2.70381400	1.13050000
H	-1.90307000	-2.72056100	1.56658900
H	4.81448300	-0.91871600	-0.23044400

**TS-C10-RAF**	**$[C_{10}H_{13}N_2O]^+ + HOO^{\bullet}$**

1 2

N	0.28972500	-2.16524300	-0.59902700
N	-4.84234900	0.70386600	0.20401000
C	-1.42057600	-0.94975400	0.20429600
C	-0.29158700	-0.08398600	-0.02013100
C	-2.76781400	-0.54272000	0.71183300
C	0.74834000	-0.88600200	-0.52775300
C	-1.01804700	-2.20436300	-0.16501100
C	-3.52283000	0.25776700	-0.34083700
C	-0.08034600	1.28916100	0.16486500
C	2.04980200	-0.37069100	-0.78443500
C	1.16643900	1.82727400	-0.18683900
C	2.18793700	1.04104400	-0.69345200
H	-3.34751400	-1.43062900	0.97670500

H	-2.65985200	0.06875700	1.61384600
H	-1.56480200	-3.13483400	-0.15441200
H	0.82269200	-2.96211100	-0.92039900
H	-3.72886300	-0.34123300	-1.22588100
H	-2.98129600	1.15661100	-0.63145400
H	-5.36992900	1.23728900	-0.49162700
H	-4.71313700	1.29729900	1.02853100
H	3.12715600	1.50711100	-0.96563200
H	-0.85794800	1.93458500	0.56212600
O	1.41531000	3.17370100	-0.05569900
H	0.63215000	3.61894700	0.29120700
H	2.73876600	-0.92924300	-1.40572500
O	2.90792900	-1.02394100	0.81958800
O	4.24996500	-0.66997900	0.76634600
H	4.28824400	0.20265700	1.18581400
H	-5.41083500	-0.10020700	0.48494100

**Product-C10-RAF**	**$[C_{10}H_{13}N_2O]^+OOH$**

1 2			
N	-0.33555500	-2.14929700	0.34000100
N	4.88764000	0.59910900	0.08860800
C	1.43395600	-0.93656100	-0.29676300
C	0.30173700	-0.06617100	-0.12665600
C	2.82245500	-0.52560800	-0.67470400

C	-0.76930600	-0.86445400	0.26795700
C	0.99727000	-2.20207700	-0.00046600
C	3.51270900	0.16911500	0.49058400
C	0.13865500	1.33259800	-0.26962400
C	-2.14354900	-0.37028000	0.51776000
C	-1.13529300	1.88015300	0.03880600
C	-2.19764900	1.12320300	0.43680900
H	3.40178900	-1.40559300	-0.96595100
H	2.79583100	0.15692700	-1.53078000
H	1.52948300	-3.14053000	-0.00568500
H	-0.90823100	-2.94735300	0.58158300
H	3.62430000	-0.49847500	1.34293700
H	2.97787300	1.06548100	0.80107700
H	5.37275700	1.06080400	0.86206200
H	4.84899700	1.25348000	-0.69808500
H	-3.13277100	1.61052800	0.68654500
H	0.95002300	1.97728100	-0.58896400
O	-1.31922800	3.24406700	-0.04929600
H	-0.47639500	3.67132000	-0.24507500
H	-2.54622400	-0.73583100	1.46883300
O	-2.96774200	-0.99895100	-0.51721800
O	-4.33271800	-0.77742700	-0.19504800
H	-4.56018100	0.00798100	-0.71575000
H	5.44974400	-0.20572400	-0.20274500

TS-C11-RAF	$[C_{10}H_{13}N_2O]^+ + HOO^{\cdot}$

1 2

N	0.42854800	2.65243800	0.00654900
N	4.34635700	-1.79654900	-0.11419400
C	1.60389100	0.77783600	0.41434200
C	0.27485500	0.41534900	-0.02395800
C	2.70990000	-0.16471300	0.76685500
C	-0.42174300	1.63130200	-0.27234500
C	1.64584800	2.14122800	0.41791200
C	3.26012000	-0.83781300	-0.48361700
C	-0.35987100	-0.79650100	-0.23946300
C	-1.74471500	1.66057600	-0.76992500
C	-1.75010000	-0.77409700	-0.56601600
C	-2.38040700	0.46947500	-0.95117100
H	3.51353100	0.38002500	1.26887400
H	2.34368600	-0.93452700	1.45427900
H	2.45227400	2.80507000	0.68991100
H	0.20817800	3.63589200	-0.07672200
H	3.69578500	-0.11146200	-1.16714100
H	2.49542600	-1.41109900	-1.00533900
H	-2.22410000	2.60107800	-1.01339100
H	4.73519500	-2.25465300	-0.94236800
H	3.99283400	-2.52428500	0.51339300

H	-3.39149600	0.41919700	-1.33679600
H	0.13444900	-1.75137000	-0.09148500
O	-2.36255000	-1.88610200	-1.06343600
H	-1.89826700	-2.67832100	-0.76072700
H	5.11081400	-1.31389900	0.36721700
O	-2.34367600	-0.85764900	1.27686600
O	-3.73772900	-0.99435200	1.26813100
H	-4.06768900	-0.08388200	1.24562900

**Product-C11-RAF**	**$[C_{10}H_{13}N_2O]^+OOH$**

1 2			
N	0.58469400	2.77987100	0.12952100
N	4.00982600	-2.04284500	-0.35375100
C	1.61002800	0.80083200	0.41376400
C	0.21368000	0.56542300	0.11068000
C	2.64729100	-0.24712400	0.66132300
C	-0.38075900	1.84045900	-0.05893500
C	1.78337200	2.15577400	0.41471800
C	3.02043100	-0.95673100	-0.63325600
C	-0.53980400	-0.58651200	-0.02175700
C	-1.75555600	2.00268900	-0.40106400
C	-2.00990100	-0.48657100	-0.28846000
C	-2.52712100	0.90404800	-0.52693900
H	3.54068400	0.20967500	1.09490100

H 2.26793900 -0.98656800 1.37494400<br>H 2.67185500 2.73812200 0.60549400<br>H 0.44779600 3.77986300 0.07086700<br>H 3.48726900 -0.27490100 -1.34159300<br>H 2.15675900 -1.42722100 -1.10036100<br>H -2.16158500 2.99331100 -0.57248700<br>H 4.27882600 -2.53128600 -1.21136200<br>H 3.62022800 -2.73264400 0.29485500<br>H -3.57291800 0.97130900 -0.80299800<br>H -0.11442000 -1.57803300 0.08660300<br>O -2.40987300 -1.29708800 -1.37244500<br>H -1.91249900 -2.12556400 -1.34247500<br>H 4.85851600 -1.65869500 0.07225500<br>O -2.76905300 -0.90959300 0.88422700<br>O -2.44369800 -2.25963500 1.18993400<br>H -3.11905900 -2.75974500 0.70677800		
**TS-C12-RAF**	**$[C_{10}H_{13}N_2O]^+ + HOO^{\bullet}$**	
1 2<br>N 0.11345500 2.44917100 -0.39641200<br>N 4.74133400 -1.21152500 0.16198100<br>C 1.57771600 0.88895100 0.30794200<br>C 0.35556000 0.23840300 -0.01373500<br>C 2.83861100 0.24070200 0.78744500		

C	-0.55730400	1.24344700	-0.44912400
C	1.38055800	2.23281600	0.05800000
C	3.51750200	-0.51703300	-0.34535900
C	-0.04914900	-1.11962500	0.00692500
C	-1.84243600	0.95029000	-0.85213900
C	-1.32793600	-1.41607400	-0.37293500
C	-2.28697800	-0.38938200	-0.70988000
H	3.52228000	0.99871100	1.17802700
H	2.61493600	-0.45895900	1.59959700
H	2.07024600	3.05487500	0.18105700
H	-0.28262800	3.35031900	-0.62319600
H	3.83817700	0.15495100	-1.13927500
H	2.86890300	-1.28522500	-0.76328200
H	-2.52049200	1.71468900	-1.21117400
H	5.21305100	-1.72543400	-0.58641700
H	4.50049800	-1.87727000	0.90216200
H	-3.17044400	-0.70549900	-1.25134800
H	0.63851200	-1.90850900	0.29451600
O	-1.82960500	-2.68599400	-0.40466900
H	-1.14967800	-3.31261400	-0.12383000
O	-3.21982400	-0.41032800	0.96096500
O	-4.34283700	0.40531700	0.82526600
H	-4.03209900	1.28760500	1.07652100
H	5.40648500	-0.53807100	0.55233000

Product-C12-RAF	$[C_{10}H_{13}N_2O]^+OOH$

1 2

N	0.18379400	2.47538900	-0.27085500
N	4.76274800	-1.25955200	-0.03387200
C	1.63486300	0.86697100	0.34306500
C	0.38413200	0.25986300	0.09023800
C	2.90560500	0.17980500	0.73741900
C	-0.52949700	1.28870400	-0.29090000
C	1.47136400	2.22387600	0.11353400
C	3.51018500	-0.55556100	-0.45089800
C	-0.05719300	-1.10454100	0.10722500
C	-1.83587800	1.05286500	-0.63130300
C	-1.34495100	-1.37809700	-0.18750400
C	-2.38001600	-0.32609600	-0.46966600
H	3.62422700	0.91374300	1.11129000
H	2.71169200	-0.53825700	1.54062900
H	2.19028400	3.02406300	0.20551400
H	-0.20129900	3.38887000	-0.46257300
H	3.78222100	0.13273700	-1.24898400
H	2.83522200	-1.31446000	-0.84323800
H	-2.49967900	1.84009000	-0.96629300
H	5.19097800	-1.75263500	-0.82139700
H	4.56764300	-1.94493700	0.70185000

H	-2.99147000	-0.63132600	-1.32561000
H	0.63737600	-1.91154500	0.32035600
O	-1.87406600	-2.63786100	-0.24097400
H	-1.19398700	-3.28663400	-0.01567100
O	-3.27776600	-0.41263700	0.69207300
O	-4.47026400	0.29273400	0.38572600
H	-4.30224400	1.17885900	0.74127300
H	5.44832000	-0.59405800	0.33407800

Table S10 Optimized structures of 12-OH-TA forms in gas phase using M06-2X/6-311++G(d,p)

Stable Neutral form			$C_{10}H_{12}N_2O$
0 1			
N	0.44868400	2.25479700	0.14815100
N	-4.33205500	-1.24353600	0.27164600
C	-1.17658000	0.76981600	-0.32563500
C	0.08463700	0.07524500	-0.22768900
C	-2.51248600	0.15120200	-0.59289800
C	1.07682200	1.03787100	0.06808800
C	-0.90500000	2.08481100	-0.09112600
C	-3.03480300	-0.65222500	0.59982400
C	0.47194000	-1.26144300	-0.37671000
C	2.42478500	0.70927800	0.22379900
C	1.80324500	-1.60282900	-0.22657500

C	2.76815600	-0.62110600	0.07326900
H	-3.24761600	0.92007600	-0.84235900
H	-2.44276600	-0.51282800	-1.46370600
H	-1.57467000	2.93100000	-0.07855400
H	0.90101000	3.13272100	0.33606400
H	-3.17222900	0.02688400	1.44501000
H	-2.27517100	-1.38754700	0.90029000
H	3.18868300	1.44234700	0.45052100
H	-4.72626400	-1.72125600	1.07376000
H	-4.23050600	-1.93440300	-0.46459000
H	-0.26076000	-2.02637300	-0.60989800
H	2.11759200	-2.63549300	-0.33957000
O	4.08741000	-0.94596800	0.22676700
H	4.20271000	-1.88993300	0.09423200
**Cation**		**$[C_{10}H_{12}N_2O]^{\bullet+}$**	
1 2			
N	0.39782000	2.25006900	0.13623900
N	-4.36929100	-1.12788400	0.32218300
C	-1.14406700	0.67290300	-0.37680000
C	0.10001000	0.03928300	-0.25833800
C	-2.47476800	0.06621900	-0.64137400
C	1.07440800	1.03004000	0.06241200
C	-0.90061700	2.04908400	-0.11694500
C	-3.03446600	-0.64840500	0.61080100

C	0.51863000	-1.30860300	-0.40668200	
C	2.39861700	0.75004900	0.24359300	
C	1.83982500	-1.62167200	-0.22959300	
C	2.77737200	-0.60731300	0.09354400	
H	-3.19334300	0.82795600	-0.94773300	
H	-2.39667200	-0.65771500	-1.45817600	
H	-1.61073500	2.86425400	-0.11463900	
H	0.82213300	3.14491400	0.34023700	
H	-3.09544500	0.07318200	1.42904700	
H	-2.32073200	-1.42614400	0.92099400	
H	3.15318300	1.48728800	0.48725100	
H	-4.88957900	-1.33714200	1.16475600	
H	-4.36413100	-1.95776500	-0.25918600	
H	-0.19994500	-2.07928100	-0.65983500	
H	2.18621900	-2.64303600	-0.33762700	
O	4.06309700	-0.86067400	0.27280400	
H	4.27490000	-1.79593000	0.15981400	
**Radical Form (O24-H)**				**$C_{10}H_{11}N_2O^{\bullet}$**
0 2				
N	0.51938300	2.24618300	0.14213300	
N	-4.30380600	-1.16167800	0.28441700	
C	-1.09413400	0.74662200	-0.34119100	
C	0.13933300	0.05846400	-0.23389300	
C	-2.43973900	0.14836500	-0.60935800	

C	1.14990800	1.01663100	0.07010200
C	-0.81480600	2.07998500	-0.10371800
C	-2.99160000	-0.60484300	0.60397300
C	0.50344700	-1.31304300	-0.38238100
C	2.47184100	0.68396900	0.23722300
C	1.80083100	-1.67859900	-0.22490400
C	2.85381100	-0.70725800	0.09513500
H	-3.15530400	0.92375200	-0.89220400
H	-2.37153900	-0.54288900	-1.45746400
H	-1.48848100	2.92373600	-0.09996300
H	0.97517900	3.12280800	0.33278300
H	-3.11115000	0.10099700	1.42990500
H	-2.25486600	-1.35388100	0.92747900
H	3.24807100	1.40318600	0.46900000
H	-4.73112300	-1.57857100	1.10314900
H	-4.22136700	-1.89368400	-0.41329500
H	-0.25809600	-2.04783700	-0.62157100
H	2.12422400	-2.70703300	-0.32920800
O	4.03275800	-1.06548500	0.23745200

**Anion (O24-H)**	**$C_{10}H_{11}N_2O^-$**

-1 1

N	0.52446900	2.24374300	0.15844000
N	-4.27082600	-1.25447100	0.26633700
C	-1.12830100	0.80102700	-0.31849400

C	0.11055700	0.07282000	-0.22035200	
C	-2.47810200	0.20923700	-0.57895800	
C	1.13471600	1.00241300	0.07322900	
C	-0.83957300	2.11668700	-0.08691900	
C	-2.97972900	-0.62977900	0.59587800	
C	0.48560000	-1.28052900	-0.37017800	
C	2.47533500	0.65946000	0.22536600	
C	1.80092800	-1.64639200	-0.22550600	
C	2.87982200	-0.70647000	0.08286000	
H	-3.21249500	0.99234500	-0.79113000	
H	-2.43146300	-0.43556200	-1.46793000	
H	-1.49232000	2.97641100	-0.07385600	
H	1.01143000	3.10799200	0.31150000	
H	-3.12642700	0.02620700	1.45794000	
H	-2.20246900	-1.35443600	0.86806300	
H	3.23515500	1.40117500	0.45049900	
H	-4.58019800	-1.84430000	1.03145900	
H	-4.14977300	-1.86600600	-0.53549700	
H	-0.26639100	-2.03245900	-0.60409300	
H	2.10177800	-2.68341600	-0.33884400	
O	4.07127000	-1.10046200	0.20711100	
**Radical Form (N1-H)**				**$C_{10}H_{11}N_2O^{\bullet}$**
0 2				

N	0.41665000	2.31754400	0.16175300
N	-4.37209000	-1.13645600	0.31206400
C	-1.14640100	0.69924200	-0.36816700
C	0.10362500	0.03962000	-0.26017800
C	-2.48319300	0.10518000	-0.63136200
C	1.04358900	1.06072500	0.06463900
C	-0.84436100	2.08785700	-0.08950000
C	-3.04700400	-0.60270800	0.61163700
C	0.53205300	-1.28257700	-0.40835300
C	2.37713900	0.78052200	0.24355100
C	1.87697900	-1.57525400	-0.22921300
C	2.78463700	-0.55563200	0.09293700
H	-3.19489700	0.87365200	-0.94123400
H	-2.41180500	-0.62456300	-1.44677800
H	-1.57532800	2.88928100	-0.08678800
H	-3.14629200	0.12880500	1.41719900
H	-2.32329300	-1.35777200	0.94821400
H	3.10153900	1.54595100	0.49053900
H	-4.81599100	-1.49779200	1.14810500
H	-4.31033400	-1.90256500	-0.34999000
H	-0.16605700	-2.07370200	-0.66064900
H	2.23649400	-2.59294100	-0.33860700
O	4.10371100	-0.80685800	0.27487000
H	4.28197800	-1.74296700	0.14837100

Anion (N1-H)	$C_{10}H_{11}N_2O^-$		
-1 1			
N	0.49017200	2.33021200	0.16827100
N	-4.34882200	-1.23479700	0.26225600
C	-1.16416700	0.78077900	-0.31687200
C	0.07760100	0.08450400	-0.22513100
C	-2.51433200	0.19314000	-0.58137600
C	1.05565000	1.10203600	0.07575000
C	-0.82975800	2.10982700	-0.06899300
C	-3.05615300	-0.61261300	0.60016300
C	0.48547700	-1.24678400	-0.37133600
C	2.41297800	0.74868400	0.22240600
C	1.82706400	-1.57562700	-0.21926300
C	2.77324200	-0.57287000	0.07564700
H	-3.23564300	0.98141200	-0.82278800
H	-2.47692500	-0.47518900	-1.45671700
H	-1.52898100	2.94249200	-0.06075400
H	-3.21036500	0.06411500	1.44457000
H	-2.29333300	-1.34144700	0.90416700
H	3.16499200	1.49788000	0.44350200
H	-4.65816600	-1.83918700	1.01610100
H	-4.22731500	-1.82910100	-0.55240600
H	-0.23437900	-2.02905800	-0.60210100

H	2.15651400	-2.60719500	-0.32221800	
O	4.11703300	-0.90848500	0.23241300	
H	4.21228200	-1.83119600	-0.01032700	
**Radical Form (N2-H)**				**$C_{10}H_{11}N_2O^{\bullet}$**
0 2				
N	0.35582900	2.24624900	0.16881500	
N	-4.29541200	-1.39794900	0.26597700	
C	-1.24043100	0.74233400	-0.34051000	
C	0.03057600	0.06651200	-0.24013500	
C	-2.56138100	0.09705900	-0.61671400	
C	1.00430500	1.04051800	0.07809600	
C	-0.99264800	2.05822400	-0.08506000	
C	-3.05671000	-0.73173000	0.56972000	
C	0.43967900	-1.26171600	-0.40687700	
C	2.35574500	0.73083900	0.24096800	
C	1.77465400	-1.58427700	-0.24825200	
C	2.72111800	-0.59199900	0.07502500	
H	-3.31292900	0.85453300	-0.85255100	
H	-2.49073400	-0.56031600	-1.48916500	
H	-1.67563400	2.89364800	-0.06715300	
H	0.79311300	3.12948300	0.36742300	
H	-3.17226200	-0.08352500	1.45303400	
H	-2.29293100	-1.47258800	0.85802700	

H	3.10634600	1.47244600	0.48368800
H	-4.58094800	-1.90274900	1.11077800
H	-0.27758200	-2.03342900	-0.66491500
H	2.10660200	-2.60970500	-0.37547700
O	4.04346500	-0.89797200	0.23631900
H	4.17687700	-1.83669900	0.08465400

**Anion (N2-H)**	**$C_{10}H_{11}N_2O^-$**

-1 1

N	0.39194100	2.26266700	0.14657700
N	-4.29382200	-1.40224600	0.16879800
C	-1.24274900	0.75733300	-0.30940400
C	0.03409300	0.08441700	-0.21902200
C	-2.56199100	0.10205300	-0.56835400
C	1.02640300	1.05195600	0.06512100
C	-0.97465500	2.07238600	-0.07581900
C	-3.10531600	-0.74472700	0.60270700
C	0.41867500	-1.25137100	-0.36301400
C	2.37871000	0.72393000	0.21183600
C	1.75461700	-1.59112000	-0.22479300
C	2.71855800	-0.60685700	0.06199500
H	-3.32188900	0.84851600	-0.81803300
H	-2.48659700	-0.57232800	-1.42953800
H	-1.65219500	2.91193700	-0.04777200

H	0.83173400	3.13733900	0.36930500
H	-3.20932900	-0.02572500	1.45773100
H	-2.25446200	-1.40513900	0.91996600
H	3.14456000	1.45720100	0.43444800
H	-4.67407600	-1.90639100	0.97217400
H	-0.33140400	-2.00743900	-0.56771100
H	2.06971600	-2.62552500	-0.32910200
O	4.04840200	-0.94144500	0.20522800
H	4.13183000	-1.89183200	0.10157900

**Inter1-O24-HAT**	**$C_{10}H_{12}N_2O$ + $HOO^{\bullet}$**

0 2

N	0.61793700	2.48167900	0.43857700
N	4.64651200	-1.77966900	-0.38836800
C	1.82676300	0.58239500	0.52191100
C	0.51047100	0.28454400	0.01183600
C	2.95144700	-0.38801500	0.70123400
C	-0.21659400	1.49860900	-0.02408300
C	1.84265300	1.92345800	0.76510400
C	3.56211900	-0.82946300	-0.63074100
C	-0.12596300	-0.88841200	-0.40932800
C	-1.54609700	1.57123400	-0.44501600
C	-1.44048700	-0.83080100	-0.84754700
C	-2.14741200	0.39414500	-0.85433100

H	3.74063600	0.05103000	1.31596100
H	2.59103300	-1.27476500	1.23748600
H	2.63881400	2.53904800	1.15472800
H	0.37503500	3.45233800	0.53947000
H	3.98088600	0.04773300	-1.13025100
H	2.76683100	-1.21889800	-1.28213400
H	-2.11322300	2.49356300	-0.45238800
H	5.12007600	-2.01876300	-1.25173800
H	4.28333200	-2.64357500	0.00056500
H	0.40243400	-1.83581300	-0.40796700
H	-1.93961800	-1.72214300	-1.21254000
O	-3.44051300	0.45534600	-1.27810000
H	-3.86338200	-0.39328800	-1.10128700
O	-3.97534300	-1.64565100	0.70450900
O	-3.06728800	-1.45571700	1.61872300
H	-2.27024300	-1.12852500	1.14335100
**TS-O24-HAT**		**$C_{10}H_{12}N_2O + HOO^{\cdot}$**	
0 2			
N	0.43080700	2.38398700	-0.13853200
N	4.90699000	-1.47927600	0.04483600
C	1.82194200	0.69098400	0.38773600
C	0.56344800	0.14459100	-0.01425100
C	3.04447100	-0.07468000	0.78602500

C	-0.29172300	1.22938100	-0.33783300
C	1.69106300	2.05504200	0.29647000
C	3.72756600	-0.74545100	-0.40844700
C	0.06627800	-1.17349800	-0.11664700
C	-1.60406200	1.05142300	-0.74634700
C	-1.22636900	-1.37065900	-0.53369600
C	-2.07795600	-0.26751200	-0.85777100
H	3.76645100	0.58351800	1.27469300
H	2.77339100	-0.84428400	1.51886500
H	2.41546800	2.82414400	0.51821300
H	0.08275300	3.31905400	-0.26852800
H	4.05398800	0.03034500	-1.10561100
H	2.99363000	-1.36616800	-0.94195200
H	-2.27560400	1.87015100	-0.97362600
H	5.42913700	-1.84961000	-0.74063500
H	4.63684600	-2.26923300	0.62137100
H	0.70288600	-2.01737400	0.12632200
H	-1.64329200	-2.36515300	-0.64239000
O	-3.31445100	-0.48829000	-1.25450200
H	-3.93540800	-0.70621900	-0.35229800
O	-4.31251500	-0.84581100	0.84885000
O	-3.36886600	-0.16115200	1.54718100
H	-2.68610800	-0.81784700	1.75778200

Inter2-O24-HAT	$C_{10}H_{12}N_2O + HOO^{\bullet}$		
0 2			
N	0.93324400	2.53023200	0.28460900
N	4.61342900	-2.04839200	-0.30550800
C	1.96716200	0.53613200	0.49552800
C	0.63595100	0.30677900	0.07798400
C	3.03294800	-0.48974700	0.72258600
C	-0.00805100	1.57302800	-0.05074800
C	2.10446400	1.90993100	0.60923300
C	3.57288600	-1.06461000	-0.59018400
C	-0.10539200	-0.88085900	-0.19953700
C	-1.31695300	1.71251200	-0.43659600
C	-1.40518800	-0.78290700	-0.57729400
C	-2.07813900	0.51364100	-0.71262800
H	3.86641000	-0.05980300	1.28249100
H	2.63142700	-1.30733700	1.33228800
H	2.97042100	2.48133100	0.90843300
H	0.77116000	3.52351400	0.30414400
H	4.01861500	-0.25314900	-1.17115500
H	2.73423800	-1.45812400	-1.18235900
H	-1.81915500	2.66707500	-0.53447300
H	5.06104400	-2.36256500	-1.15846400
H	4.21946600	-2.86830700	0.14373900

H	0.37731900	-1.84826500	-0.10937500	
H	-2.00303200	-1.65754500	-0.80875900	
O	-3.27351700	0.58601000	-1.05355800	
H	-4.24630100	-0.84366600	-0.40795800	
O	-4.42748900	-1.61837100	0.15858500	
O	-3.88855600	-1.17721200	1.40036400	
H	-3.08995700	-1.71285400	1.47454700	
**TS-C7-RAF**				**$C_{10}H_{12}N_2O$ + HOO$^{\bullet}$**
0 2				
N	-0.35197800	-1.53370700	1.02604300	
N	4.03672200	2.30792200	0.06472700	
C	1.09198300	-0.06675900	0.06289400	
C	-0.22695300	0.45192400	-0.05121500	
C	2.36362400	0.57489600	-0.37571800	
C	-1.11086000	-0.50189300	0.52028200	
C	0.96411800	-1.34316400	0.62985900	
C	2.78324100	1.72856900	0.54204000	
C	-0.76074900	1.62548500	-0.60685600	
C	-2.48847500	-0.32610300	0.53193600	
C	-2.12707300	1.81547500	-0.59245900	
C	-2.98043100	0.84254800	-0.03079600	
H	3.16883400	-0.16230900	-0.40674400	
H	2.24468400	0.95883900	-1.39661900	

H	-0.71423600	-2.46750600	1.15679500
H	2.94985400	1.33519000	1.54806100
H	1.95613800	2.44922700	0.61228600
H	-3.17195200	-1.05124700	0.95433600
H	4.37188800	3.02312000	0.69952900
H	3.90502600	2.74865200	-0.83959400
H	-0.11048100	2.37150300	-1.04970200
H	-2.55993000	2.71390000	-1.02006200
O	-4.33013600	1.01360100	-0.01446400
H	-4.55915000	1.84069500	-0.44611400
H	1.75134800	-1.92514900	1.08512900
O	1.02190000	-2.50621900	-0.98600800
O	0.61864800	-3.73573300	-0.48924300
H	1.44093200	-4.23348300	-0.39478300

**Product-C7-RAF**	**$C_{10}H_{12}N_2OOOH$**

0 2

N	0.25804000	1.64275400	0.81395000
N	-3.81659600	-2.55499300	0.21659100
C	-0.99099200	-0.06597000	-0.12961200
C	0.36399800	-0.42418100	-0.15009800
C	-2.17286800	-0.88502100	-0.50950300
C	1.12781000	0.66433200	0.38111700
C	-1.08203200	1.35078600	0.35634200

C	-2.64266900	-1.79290800	0.63644200
C	1.03826800	-1.58093500	-0.57713800
C	2.50334900	0.62162300	0.45579500
C	2.42198000	-1.63232200	-0.49832600
C	3.14150300	-0.54099600	0.00564100
H	-3.00455900	-0.24266000	-0.80976700
H	-1.92444600	-1.51227900	-1.37440200
H	0.53738500	2.60613300	0.91658700
H	-2.92846100	-1.16945200	1.48792100
H	-1.79686800	-2.41529700	0.96100400
H	3.09590700	1.43527300	0.85399500
H	-4.18710500	-3.10397700	0.98357100
H	-3.57303200	-3.20394800	-0.52431700
H	0.48284700	-2.42204300	-0.97574500
H	2.95724200	-2.51553200	-0.83085900
O	4.49918900	-0.55544400	0.09144500
H	4.83582200	-1.39196500	-0.24043200
H	-1.82479900	1.54018000	1.13934900
O	-1.46272300	2.14094300	-0.77250000
O	-1.61320400	3.47812400	-0.26511200
H	-1.39621800	3.98580900	-1.05531600

**TS-C9-RAF**	**$C_{10}H_{12}N_2O + HOO^{\cdot}$**
0 2	

N	-0.22047600	-2.55194300	-0.63472700
N	4.38408200	0.84663400	0.91368100
C	1.29645000	-0.90339300	-0.40815000
C	0.02226600	-0.42271600	0.00872500
C	2.57629300	-0.12842400	-0.41560100
C	-0.91195400	-1.46448500	-0.15626400
C	1.10412100	-2.21039100	-0.78736300
C	3.14788900	0.07010500	0.98956600
C	-0.43790700	0.86950900	0.40214100
C	-2.26586300	-1.32007400	0.13498900
C	-1.79990900	0.98066600	0.79271800
C	-2.68375500	-0.07338600	0.61937600
H	3.32716500	-0.63143200	-1.02929400
H	2.38938400	0.85116400	-0.87034900
H	1.81817500	-2.92617100	-1.16480500
H	-0.62128500	-3.44725900	-0.85695900
H	3.38317900	-0.90788300	1.41784800
H	2.37800800	0.52323600	1.63068200
H	-2.98913400	-2.11604600	0.01101200
H	4.80418600	0.95221500	1.82980600
H	4.19344900	1.77991800	0.56368900
H	0.26784800	1.59142600	0.79498600
H	-2.15882800	1.92380300	1.19209400
O	-4.00963900	0.02913900	0.92957900

H	-4.19542300	0.91251800	1.25819900
O	-0.32764600	1.70370900	-1.30772500
O	-0.71102100	3.02110900	-1.16939000
H	-1.64130600	3.01553400	-1.43012000

**Product-C9-RAF**	**$C_{10}H_{12}N_2OOOH$**

0 2

N	0.34397500	2.62824700	-0.39660100
N	-4.36727500	-0.73742000	0.78126100
C	-1.21362400	1.01159600	-0.37593800
C	0.04368700	0.44128500	-0.07658700
C	-2.53246300	0.30547500	-0.45508900
C	1.00505900	1.45854800	-0.10153900
C	-0.98983900	2.36487200	-0.56315600
C	-3.09310400	-0.03584900	0.92694800
C	0.43161200	-0.97700200	0.16468700
C	2.36632200	1.25332600	0.13996300
C	1.89185000	-1.12539700	0.47605100
C	2.76389500	-0.08358100	0.43918400
H	-3.26465800	0.92016700	-0.98395500
H	-2.40637600	-0.61888600	-1.02772300
H	-1.68759200	3.14928900	-0.81124800
H	0.77492200	3.53412300	-0.47528400
H	-3.27279200	0.89201600	1.47664300

H	-2.33903500	-0.60271000	1.49139700
H	3.10713500	2.04081500	0.11760300
H	-4.78319700	-0.92585200	1.68599200
H	-4.22356800	-1.63390800	0.32775300
H	-0.17254700	-1.43120300	0.96064200
H	2.24093700	-2.11634700	0.74735800
O	4.09718900	-0.21825300	0.70408800
H	4.29300300	-1.14031700	0.89115200
O	0.05655700	-1.67534300	-1.04975800
O	0.20717700	-3.06902800	-0.80247200
H	0.96911000	-3.28161800	-1.35496200

**TS-C10-RAF**	**$C_{10}H_{12}N_2O$ + $HOO^{\bullet}$**

0 2			
N	0.08019200	-1.97189800	-0.58973900
N	-5.10965700	0.78210000	0.08860200
C	-1.66778400	-0.79965000	0.19901200
C	-0.52363300	0.06822700	0.09192700
C	-3.04317800	-0.41114100	0.64168900
C	0.53906800	-0.70147400	-0.40339100
C	-1.24880100	-2.03218600	-0.21826700
C	-3.77836800	0.43083400	-0.40344100
C	-0.30521500	1.42945800	0.37827600
C	1.85829800	-0.20333700	-0.57651200

C	0.95402400	1.97376600	0.16510100
C	1.99306000	1.19170400	-0.34101100
H	-3.64146100	-1.30005900	0.85471400
H	-2.98096000	0.15860600	1.57709900
H	-1.79713100	-2.95912600	-0.28199000
H	0.65735100	-2.76047500	-0.83227700
H	-3.89531600	-0.16442500	-1.31252400
H	-3.15835800	1.29955500	-0.66752600
H	-5.64167300	1.27337500	-0.62021500
H	-5.04127200	1.39948700	0.89086500
H	-1.10560600	2.04967100	0.76491600
H	1.13798600	3.02121300	0.38123500
O	3.24229500	1.69124700	-0.55665700
H	3.25412600	2.63168500	-0.35535700
H	2.55267300	-0.67781900	-1.26005700
O	2.82430100	-0.90856500	0.87997700
O	4.14908100	-0.94079400	0.48556900
H	4.47565300	-0.05540900	0.69767200

**Product-C10-RAF**	**$C_{10}H_{12}N_2OOOH$**

0 2

N	-0.12182600	-1.99429800	0.26133500
N	5.10624700	0.75121100	0.19345400
C	1.67485800	-0.78000200	-0.29711600

C	0.53409900	0.08845300	-0.18232200
C	3.07807200	-0.36477400	-0.61014000
C	-0.55002400	-0.70672000	0.16037700
C	1.22502400	-2.04601900	-0.02738500
C	3.74437000	0.36337100	0.55905000
C	0.36198300	1.48902700	-0.36936000
C	-1.93375200	-0.23597100	0.39338300
C	-0.92840800	2.02450400	-0.21320800
C	-1.99743700	1.24099300	0.13711500
H	3.68596200	-1.23462800	-0.86989500
H	3.08019500	0.29503500	-1.48659400
H	1.75729100	-2.98397900	-0.02516000
H	-0.72705000	-2.78874000	0.39456600
H	3.79968400	-0.31990300	1.41030400
H	3.10884500	1.20648100	0.86590800
H	5.58826200	1.17039800	0.98013300
H	5.09147900	1.44284300	-0.54883600
H	1.19025200	2.12581600	-0.65036500
H	-1.09228600	3.08572600	-0.37724900
O	-3.27442000	1.71248200	0.26667600
H	-3.30575600	2.64605900	0.03573000
H	-2.29998000	-0.46824600	1.40403300
O	-2.80888500	-0.95698500	-0.50451700
O	-4.11376300	-0.98259400	0.05433100

H	-4.44400700	-0.09564500	-0.15038700

**TS-C11-RAF**	**$C_{10}H_{12}N_2O + HOO^{\bullet}$**

0 2			
N	-0.63664700	2.55419200	-0.28333200
N	-4.52384900	-1.86602300	0.40983100
C	-1.82763400	0.64853300	-0.45909800
C	-0.46755000	0.32790400	-0.07638300
C	-2.94255800	-0.32886400	-0.65360800
C	0.24123900	1.55909600	0.02484500
C	-1.88027200	2.00146000	-0.57705200
C	-3.44851600	-0.90980100	0.66876600
C	0.19146300	-0.85600500	0.20418300
C	1.59720400	1.63513000	0.37913600
C	1.58107300	-0.82847700	0.48778200
C	2.24595300	0.45239000	0.62011000
H	-3.78155000	0.14507000	-1.16824400
H	-2.59992500	-1.15011200	-1.29506700
H	-2.70718800	2.63587400	-0.85627700
H	-0.41400700	3.53463500	-0.31841400
H	-3.85310000	-0.09595700	1.27577900
H	-2.60109900	-1.33376000	1.22579300
H	2.13710900	2.57104700	0.44342700
H	-4.92575000	-2.20446000	1.27632200

H	-4.16821400	-2.67488200	-0.08900400
H	-0.32376100	-1.80899200	0.17956000
H	2.00363600	-1.64859400	1.05890200
O	3.55452900	0.46764200	0.95177800
H	3.95893300	-0.35274600	0.62806200
O	2.44069200	-1.43492100	-1.06956400
O	3.74738300	-1.77194300	-0.68236400
H	3.71514100	-2.72871600	-0.55720300

**Product-C11-RAF**	**$C_{10}H_{12}N_2OOOH$**

0 2

N	-0.66479800	2.59541100	-0.18907100
N	-4.44672300	-1.92683000	0.41467800
C	-1.83877600	0.69028200	-0.42209900
C	-0.45786900	0.36279800	-0.12464000
C	-2.94877100	-0.29055700	-0.62411400
C	0.23353100	1.59116300	0.01506200
C	-1.91477300	2.04959200	-0.45431900
C	-3.37847800	-0.96375900	0.68120600
C	0.21337300	-0.83605800	0.02790900
C	1.62124500	1.66890800	0.31380500
C	1.67670000	-0.85117700	0.28925500
C	2.30548700	0.51120400	0.45245000
H	-3.81938800	0.19999300	-1.06573600

H	-2.62680000	-1.06414000	-1.33259600
H	-2.76113000	2.68848700	-0.65237700
H	-0.45308700	3.57859900	-0.16864100
H	-3.76875500	-0.19852200	1.35694100
H	-2.49604400	-1.40215100	1.16806500
H	2.13789400	2.61373800	0.43067500
H	-4.79423400	-2.33129600	1.27643400
H	-4.10027200	-2.69235000	-0.15415800
H	-0.28546100	-1.79325500	-0.05899500
H	1.93237800	-1.47007000	1.16375400
O	3.61816500	0.53722900	0.78064500
H	4.01943100	-0.30956900	0.54415000
O	2.27854200	-1.54501900	-0.85220200
O	3.62323800	-1.89712100	-0.50518000
H	3.56654800	-2.85461100	-0.39996900

**TS-C12-RAF**	**$C_{10}H_{12}N_2O + HOO^{\cdot}$**

0 2			
N	0.41075700	2.40214200	-0.09810100
N	4.84620700	-1.50641800	-0.05581900
C	1.79273600	0.69054200	0.39103000
C	0.52007500	0.16013000	0.03892200
C	3.02062100	-0.08711700	0.74864800
C	-0.33681600	1.25371300	-0.25892900

C	1.67946200	2.05972600	0.29684800
C	3.66006300	-0.75870400	-0.46888300
C	0.01372700	-1.16384900	-0.07215300
C	-1.64881000	1.09525600	-0.65235900
C	-1.28431300	-1.34971100	-0.42604400
C	-2.17972200	-0.22370300	-0.61886700
H	3.76344200	0.56259900	1.21726800
H	2.76393900	-0.85679900	1.48622700
H	2.42200800	2.81903300	0.49012900
H	0.06359200	3.34077700	-0.19845800
H	3.97221100	0.01665000	-1.17322700
H	2.90379400	-1.37023300	-0.98122300
H	-2.30641300	1.91550500	-0.90870100
H	5.33033100	-1.89197500	-0.85820900
H	4.58565500	-2.28732100	0.53737400
H	0.66105000	-2.01263600	0.11720800
H	-1.71077200	-2.34241000	-0.51347600
O	-3.33119800	-0.41558400	-1.31760400
H	-3.81281100	-1.13934500	-0.89008600
O	-2.89698200	-0.18720700	1.16479100
O	-4.03433300	-0.98891500	1.12488200
H	-4.75681600	-0.34795900	1.10679400

**Product-C12-RAF**	**$C_{10}H_{12}N_2OOOH$**

0 2			
N	0.48055800	2.43418000	-0.05900800
N	4.78172500	-1.59907900	-0.20607500
C	1.82482300	0.68665100	0.40827700
C	0.52042900	0.19825800	0.17930000
C	3.05267100	-0.11944100	0.69872800
C	-0.32479200	1.30899700	-0.11042000
C	1.76125900	2.06201200	0.25563600
C	3.58389800	-0.83389600	-0.54651900
C	-0.04374000	-1.12271300	0.15681000
C	-1.65635900	1.20206600	-0.39787400
C	-1.35358200	-1.29204100	-0.09618300
C	-2.30404100	-0.14184600	-0.33727800
H	3.84518900	0.51787100	1.09774000
H	2.82895600	-0.86580700	1.46992300
H	2.54599500	2.79605800	0.35567000
H	0.16315900	3.37883200	-0.19599600
H	3.85793200	-0.08235300	-1.29131500
H	2.77742200	-1.43880600	-0.98483900
H	-2.29480300	2.03842600	-0.64973500
H	5.19991800	-2.00677100	-1.03416500
H	4.55016900	-2.36491300	0.41791400
H	0.59788900	-1.98164200	0.32458200

H	-1.78806100	-2.28399300	-0.15320900
O	-3.05817300	-0.33269500	-1.50268100
H	-3.63477100	-1.09153100	-1.35012200
O	-3.21730800	-0.01855000	0.78355300
O	-4.00095400	-1.21130900	0.83998600
H	-3.63689000	-1.64892200	1.61939700

Table S11 Optimized structures of 12-OH-TA forms in water using M06-2X/6-311++G(d,p)

**Stable Cationic form**			**$[C_{10}H_{13}N_2O]^+$**
1 1			
N	0.52159900	2.26521700	0.15166100
N	-4.27612700	-1.23906800	0.27098600
C	-1.11909700	0.79814800	-0.31388300
C	0.13162800	0.08530800	-0.22179500
C	-2.46425600	0.20430200	-0.59285600
C	1.13345000	1.04151200	0.06903200
C	-0.83034800	2.11259900	-0.07948400
C	-2.94901100	-0.62545900	0.58754800
C	0.50245200	-1.25947200	-0.36826500
C	2.48087100	0.69875400	0.21885800
C	1.83153900	-1.61388700	-0.22319300
C	2.80478000	-0.63669400	0.06843100
H	-3.18527200	1.00115800	-0.79416700

H	-2.41773000	-0.43610500	-1.48045900	
H	-1.48837900	2.96842500	-0.06338000	
H	0.98669900	3.14159400	0.33796900	
H	-3.08512900	-0.01321200	1.47706500	
H	-2.26911900	-1.44504100	0.81424200	
H	3.24462900	1.43472000	0.43972700	
H	-4.62119700	-1.80322600	1.05131600	
H	-4.20960200	-1.84441000	-0.55237500	
H	-0.24110500	-2.01670600	-0.59321500	
H	2.14488900	-2.64702600	-0.33035800	
O	4.12951300	-0.98762600	0.21548800	
H	4.22777500	-1.93728600	0.07442900	
H	-4.97291500	-0.51558100	0.07041200	
	**Cation radical**			**$[C_{10}H_{13}N_2O]^{\bullet+}$**
2 2				
N	0.47940800	2.26441700	0.14224800	
N	-4.28386400	-1.17733700	0.32694700	
C	-1.08428100	0.71795000	-0.36226600	
C	0.14189300	0.05904000	-0.26234800	
C	-2.42608600	0.12867100	-0.63480900	
C	1.13291700	1.03581200	0.05770100	
C	-0.81860800	2.08917500	-0.09935800	
C	-2.94919900	-0.57416400	0.61383200	
C	0.53325700	-1.29919700	-0.41306900	

C	2.45151200	0.73345900	0.23698600	
C	1.84830400	-1.63134400	-0.23986800	
C	2.80131600	-0.62991500	0.08688200	
H	-3.12208600	0.91549000	-0.93346500	
H	-2.34980500	-0.59625700	-1.45051600	
H	-1.51400300	2.91640300	-0.08631300	
H	0.92563100	3.15168300	0.34788800	
H	-3.08068000	0.12264000	1.43923000	
H	-2.28941200	-1.38337700	0.92158000	
H	3.20738700	1.46782100	0.48447600	
H	-4.66269300	-1.64686700	1.15375900	
H	-4.21790700	-1.86774600	-0.42681100	
H	-0.20348400	-2.05364400	-0.66263100	
H	2.19204300	-2.65382800	-0.34421700	
O	4.08361500	-0.92774000	0.27155200	
H	4.24543000	-1.87738400	0.16236700	
H	-4.95430700	-0.45949400	0.03578200	
**Radical Form (O24-H)**				**$[C_{10}H_{12}N_2O]^{+\bullet}$**
1 2				
N	0.58607500	2.24791400	0.14487600	
N	-4.24770600	-1.15393600	0.29741900	
C	-1.03243600	0.75711200	-0.34128300	
C	0.18478200	0.05916500	-0.23736100	
C	-2.38538900	0.18282200	-0.62023300	

C	1.20471600	1.01284000	0.06940000
C	-0.73768700	2.09683200	-0.09722400
C	-2.91350500	-0.55392500	0.60306500
C	0.53872500	-1.31599900	-0.38344000
C	2.52084200	0.67217800	0.23660100
C	1.83594800	-1.68572500	-0.22435900
C	2.88361700	-0.71800900	0.09309300
H	-3.07854400	0.98287200	-0.89115600
H	-2.32853900	-0.51459600	-1.46190100
H	-1.40432500	2.94674800	-0.08960500
H	1.05092100	3.12488900	0.33901000
H	-3.05065800	0.11965700	1.44679300
H	-2.25632300	-1.37122700	0.89542300
H	3.29120300	1.39785900	0.46920600
H	-4.63082900	-1.64305900	1.11031900
H	-4.17667000	-1.82578300	-0.47224400
H	-0.22770400	-2.04627700	-0.61988700
H	2.14631800	-2.71901200	-0.32780800
O	4.07698500	-1.09041200	0.23719900
H	-4.91699300	-0.43036300	0.01863500
**Anion (O24-H)**		**[$C_{10}H_{12}N_2O$]**	
2 1			
N	0.50817100	2.23632400	0.10682700
N	-4.31906500	-1.04324100	0.34225000

C	-0.99715200	0.61223900	-0.38697000
C	0.21230200	-0.00322500	-0.26596500
C	-2.33485000	0.01578500	-0.66913100
C	1.20350300	1.01098500	0.05060500
C	-0.75636800	2.02005400	-0.13862300
C	-2.98450800	-0.44188500	0.63262100
C	0.62075200	-1.38933200	-0.39098200
C	2.49615800	0.75581600	0.24639900
C	1.90981500	-1.70473200	-0.20328100
C	2.92349000	-0.67285900	0.12494300
H	-2.97053800	0.75610700	-1.16020900
H	-2.21791100	-0.83990400	-1.33823500
H	-1.47929600	2.82647500	-0.14838700
H	0.93119000	3.14130900	0.29968400
H	-3.14593700	0.38944700	1.31632100
H	-2.38611900	-1.20694200	1.12407300
H	3.24138000	1.50424200	0.48815000
H	-4.76388000	-1.38327600	1.19917400
H	-4.23074600	-1.83434400	-0.30275000
H	-0.12553000	-2.13732800	-0.63093200
H	2.27703600	-2.72056700	-0.28087100
O	4.09162200	-0.96348100	0.29757000
H	-4.94521700	-0.35604100	-0.08781900

Radical Form (N1-H)	$[C_{10}H_{12}N_2O]^{+\bullet}$		
1 2			
N	0.50125300	2.34255900	0.17291100
N	-4.28082500	-1.17001500	0.31331100
C	-1.08806100	0.75089400	-0.34886700
C	0.14480300	0.07241700	-0.25688200
C	-2.43692800	0.18297500	-0.61813200
C	1.10743400	1.07260800	0.06620100
C	-0.76984300	2.13211900	-0.06908800
C	-2.93091100	-0.59695700	0.59683100
C	0.54318800	-1.26499300	-0.41351500
C	2.43491800	0.76192400	0.23870000
C	1.87839600	-1.58798200	-0.24314000
C	2.80822600	-0.58635300	0.08279300
H	-3.14107700	0.98677900	-0.84645100
H	-2.39173800	-0.49348800	-1.47827600
H	-1.49373400	2.93860800	-0.05734800
H	-3.03193300	0.04427500	1.47020300
H	-2.27248100	-1.43096300	0.83190500
H	3.18228400	1.50605600	0.48775200
H	-4.63324700	-1.69926900	1.11500500
H	-4.24743900	-1.80107600	-0.49279600
H	-0.17818300	-2.03488400	-0.66490100

H	2.22575000	-2.60894100	-0.35476500
O	4.12119800	-0.87322800	0.26540500
H	4.27731200	-1.82055100	0.15135600
H	-4.95542700	-0.42844700	0.10211700

**Anion (N1-H)**	**[$C_{10}H_{12}N_2O$]**

2 1

N	0.30030000	2.23918800	0.00750700
N	-4.53112200	-0.85365800	0.32036000
C	-1.02952300	0.35511800	-0.36909500
C	0.23849800	-0.11467500	-0.25420700
C	-2.30350800	-0.35641900	-0.60217600
C	1.07891700	1.03670100	-0.00715700
C	-0.90345600	1.85258600	-0.19409400
C	-3.22536700	-0.19592300	0.60668600
C	0.80429200	-1.42955900	-0.30587700
C	2.40253100	0.92147600	0.18880100
C	2.14206700	-1.57320400	-0.11477600
C	2.94203500	-0.41765100	0.13393100
H	-2.78646800	0.09641700	-1.47732200
H	-2.12015900	-1.41299200	-0.80335400
H	-1.74161700	2.53848900	-0.24287800
H	-3.43377500	0.84984500	0.82516900
H	-2.80715700	-0.67621700	1.48837200

H	3.06691900	1.75350000	0.38440800
H	-5.16500700	-0.76558600	1.11947400
H	-4.40617000	-1.85186200	0.12510900
H	0.16920100	-2.28777000	-0.49356400
H	2.63098300	-2.53931800	-0.13967300
O	4.22059100	-0.50346200	0.32936000
H	4.55202800	-1.41754600	0.28551000
H	-4.98667100	-0.42868000	-0.49343600
**Radical Form (N2-H)**		**$[C_{10}H_{12}N_2O]^{+\bullet}$**	
1 2			
N	-0.38421400	2.24986000	-0.15199900
N	4.30517200	-1.26981400	-0.30209600
C	1.14645200	0.67371800	0.37678700
C	-0.09752000	0.04188900	0.27502600
C	2.47204300	0.05503600	0.64584000
C	-1.06455700	1.03431200	-0.05940400
C	0.90819500	2.04992400	0.09796000
C	3.00197100	-0.67043900	-0.59595000
C	-0.51923500	-1.30305300	0.43484000
C	-2.38914200	0.75917200	-0.24664200
C	-1.84202700	-1.60953800	0.25298400
C	-2.76925600	-0.59358400	-0.08982100
H	3.18386300	0.82852000	0.94540800

H	2.38212900	-0.66466000	1.46384800
H	1.62045200	2.86259300	0.08003700
H	-0.81131500	3.14393800	-0.36832500
H	3.03318200	0.03818800	-1.43192200
H	2.30443100	-1.46563900	-0.86660000
H	-3.12572000	1.50857500	-0.50693400
H	4.62924300	-1.77057800	-1.12439700
H	0.19831800	-2.07184500	0.69675500
H	-2.20598100	-2.62432100	0.36319400
O	-4.05952700	-0.86672400	-0.28684700
H	-4.23919600	-1.81279000	-0.17801900
H	4.97855900	-0.52313100	-0.14932400
**Anion (N2-H)**		**[$C_{10}H_{12}N_2O$]**	
2 1			
N	-0.21611500	2.16352000	-0.02703300
N	4.50223000	-1.02222300	-0.34680400
C	1.09929900	0.35194600	0.40968000
C	-0.17070600	-0.11365000	0.27191300
C	2.37163300	-0.35263500	0.64680100
C	-1.03882600	1.00112300	-0.01175100
C	1.00667100	1.82660300	0.20684200
C	3.22485500	-0.37256000	-0.63372400
C	-0.73128700	-1.43813700	0.34581000

C	-2.35221200	0.89416100	-0.22211600
C	-2.06119100	-1.60158200	0.14506700
C	-2.87986500	-0.46308900	-0.13606900
H	2.92522400	0.18324900	1.42655500
H	2.17647300	-1.37127100	0.98212600
H	1.81306800	2.54983300	0.24774100
H	-0.55140900	3.11437600	-0.18901600
H	3.33681100	0.65048400	-1.01186200
H	2.69731400	-0.94941400	-1.39447800
H	-3.02032100	1.71722600	-0.44211500
H	5.02969900	-1.09587500	-1.21195000
H	-0.08452000	-2.28057800	0.56047300
H	-2.53907700	-2.57254100	0.18776600
O	-4.14458000	-0.55545000	-0.33364300
H	-4.48650000	-1.46687800	-0.26664500
H	5.04683700	-0.41717300	0.26268500

**Inter1-O24-HAT**	**$[C_{10}H_{13}N_2O]^+ + HOO^•$**

1 2

N	1.22800700	2.59640200	0.07330000
N	4.32630800	-2.46340600	-0.33430100
C	2.15410500	0.57111000	0.39199000
C	0.73749700	0.40746800	0.17365000
C	3.14777400	-0.52101200	0.63547300

C	0.19503300	1.70000000	-0.01877600
C	2.40145300	1.91261500	0.31968600
C	3.37219400	-1.34777300	-0.62281600
C	-0.12287900	-0.69897200	0.13048800
C	-1.16710500	1.91822300	-0.24660300
C	-1.47305600	-0.49934200	-0.09577900
C	-1.98539900	0.80316900	-0.27842400
H	4.09989600	-0.09142200	0.95861400
H	2.79243400	-1.18313200	1.43275200
H	3.33299900	2.44681800	0.43121300
H	1.14393400	3.59826300	-0.01831700
H	3.81130700	-0.75111600	-1.42018400
H	2.45054100	-1.80478800	-0.97921400
H	-1.57727900	2.91086300	-0.38957400
H	4.50772900	-3.02561200	-1.16933900
H	3.95263300	-3.08231300	0.39070400
H	0.26159600	-1.70371000	0.27104200
H	-2.15231500	-1.34345600	-0.14377300
O	-3.32758500	1.01068700	-0.49433500
H	-3.80213300	0.16243900	-0.50517300
O	-4.82609600	-1.43572700	-0.38190000
O	-4.65384000	-1.95673400	0.79549000
H	-4.02034700	-1.38682200	1.27884400
H	5.22126200	-2.09571700	0.00204200

TS-O24-HAT	$[C_{10}H_{13}N_2O]^+ + HOO^{\cdot}$

1 2			
N	0.39939500	2.42528300	-0.06680400
N	4.82727500	-1.52152100	-0.03480400
C	1.78210600	0.70981900	0.39508200
C	0.52471600	0.17765400	-0.01303400
C	3.00300800	-0.06561100	0.77843400
C	-0.32714800	1.28034800	-0.29541400
C	1.65480000	2.08184400	0.34532100
C	3.64163700	-0.70805600	-0.44533100
C	0.02439800	-1.13642400	-0.15715300
C	-1.64142900	1.12155700	-0.70321100
C	-1.27181100	-1.31238900	-0.57350300
C	-2.10946700	-0.19242500	-0.85004000
H	3.72602900	0.59702100	1.26055600
H	2.73755800	-0.85042200	1.49425000
H	2.38273700	2.84289800	0.58410000
H	0.05420600	3.36961900	-0.16958600
H	3.99697100	0.04094400	-1.15060100
H	2.95376400	-1.38333800	-0.95165800
H	-2.29722400	1.95939000	-0.90786500
H	5.27117800	-1.96420900	-0.84352400
H	4.55216500	-2.25981700	0.61952800

H	0.65870500	-1.98965100	0.05697400
H	-1.69337800	-2.30275600	-0.70213100
O	-3.36499300	-0.39691300	-1.28066900
H	-3.94827600	-0.72257900	-0.45601000
O	-4.40217300	-0.92226700	0.82622300
O	-3.41303000	-0.36029800	1.56157900
H	-2.75689900	-1.06374900	1.71363100
H	5.52788800	-0.93671800	0.42980300

**Inter2-O24-HAT**	**$[C_{10}H_{13}N_2O]^+ + HOO^•$**

1 2

N	0.14799900	2.08955500	-0.16676400
N	5.31372600	-0.76528500	-0.32060800
C	1.90546700	0.76941500	0.33342000
C	0.76270600	-0.04304100	0.25394600
C	3.31122100	0.33969700	0.61018500
C	-0.34765100	0.80137300	-0.06255500
C	1.47934800	2.07190300	0.06596100
C	3.91334500	-0.33391400	-0.61563900
C	0.54335700	-1.44279200	0.42935800
C	-1.62519400	0.33380600	-0.21177900
C	-0.71369700	-1.93763800	0.28990100
C	-1.84582500	-1.07963400	-0.03455800
H	3.91573500	1.20823100	0.88262900

H	3.32856300	-0.36186700	1.44956100
H	2.06146500	2.98127500	0.03688600
H	-0.40080500	2.91311900	-0.37700900
H	3.96034200	0.34716200	-1.46337900
H	3.35692500	-1.22632000	-0.89779600
H	-2.46312300	0.97801200	-0.45182400
H	5.74081000	-1.21930400	-1.13219200
H	5.33434600	-1.42711000	0.46072000
H	1.37773000	-2.09191000	0.67203700
H	-0.92471800	-2.99303800	0.41571300
O	-3.00233700	-1.57665400	-0.15617100
H	-4.27626800	-0.57039200	-0.55417200
O	-5.03228700	0.05668900	-0.69059000
O	-4.94811900	0.87294600	0.47243900
H	-5.57505600	0.45577200	1.07975100
H	5.89544900	0.03758100	-0.06418400

**TS-C7-RAF**	**$[C_{10}H_{13}N_2O]^+$ + HOO$^{\bullet}$**

1 2

N	0.47256600	1.64169800	1.17571700
N	-3.61238600	-2.53109500	0.16964500
C	-0.92343200	0.14032700	0.22156000
C	0.41321300	-0.27377000	-0.01992300
C	-2.17693500	-0.55396200	-0.19221300

C	1.26738600	0.69133300	0.57852000
C	-0.84565800	1.37795700	0.89201700
C	-2.37740300	-1.82441700	0.62609400
C	0.98588000	-1.36880200	-0.69380900
C	2.65288100	0.59684500	0.52582800
C	2.35907900	-1.47714200	-0.75056000
C	3.17923300	-0.50009300	-0.14222400
H	-3.03383900	0.10876400	-0.04918500
H	-2.12376500	-0.81965400	-1.25430100
H	0.80858400	2.48418700	1.62041800
H	-2.50795400	-1.60136700	1.68323500
H	-1.54854100	-2.51910100	0.50207700
H	3.30402700	1.33303400	0.98066400
H	-3.76855500	-3.38753800	0.70717700
H	-3.54211100	-2.78625200	-0.81982900
H	0.35647300	-2.11717600	-1.16247000
H	2.83137300	-2.30859400	-1.26192300
O	4.54177300	-0.60007500	-0.18866500
H	4.80202000	-1.40413400	-0.65595500
H	-1.63567600	1.87730000	1.43143600
O	-1.12037800	2.59160200	-0.70156800
O	-2.45132700	2.57635400	-1.09667600
H	-2.49245800	1.90722600	-1.79602900
H	-4.43685600	-1.93363100	0.28066700

Product-C7-RAF			[$C_{10}H_{13}N_2O$]$^+$OOH
1 2			
N	0.28967000	1.81133200	0.50327200
N	-3.52708100	-2.73369300	0.33516100
C	-0.91238600	-0.04827700	-0.22617600
C	0.45461900	-0.35275700	-0.25661200
C	-2.05402100	-0.95009900	-0.54230700
C	1.17516500	0.77574100	0.24194600
C	-1.06833600	1.34372600	0.31089300
C	-2.38177100	-1.82965700	0.66153600
C	1.16544600	-1.51156000	-0.62474800
C	2.54432600	0.75313200	0.40298700
C	2.54308300	-1.53789600	-0.47584300
C	3.21707600	-0.42100600	0.03917800
H	-2.93847500	-0.36620100	-0.81230800
H	-1.80075500	-1.59285900	-1.39171100
H	0.48050800	2.42711900	1.28254600
H	-2.67990900	-1.23531300	1.52345900
H	-1.53998200	-2.46494600	0.93115100
H	3.09606800	1.60093500	0.79074700
H	-3.76892600	-3.33008200	1.13036200
H	-3.29716400	-3.33864800	-0.45848500
H	0.63779600	-2.37778000	-1.00788000

H	3.11530200	-2.41912900	-0.74471200
O	4.57399700	-0.42540700	0.20853400
H	4.93659200	-1.27456200	-0.07487500
H	-1.66365100	1.42221000	1.22698600
O	-1.76124800	2.09862900	-0.69529900
O	-2.09802400	3.36816400	-0.14793300
H	-1.37509200	3.93682100	-0.45423600
H	-4.35743600	-2.19020600	0.08238400

**TS-C9-RAF**	**$[C_{10}H_{13}N_2O]^+$ + HOO$^\bullet$**

1 2

N	0.25476000	-2.55809600	0.64882300
N	-4.33132400	0.85850900	-0.93512600
C	-1.25982900	-0.91034000	0.43202700
C	0.00653900	-0.42324200	0.00567400
C	-2.55286500	-0.15675900	0.44612400
C	0.94017000	-1.47098900	0.16233500
C	-1.06052400	-2.22290100	0.80886600
C	-3.06667900	0.06136900	-0.96985400
C	0.46902600	0.86155500	-0.40836400
C	2.28846300	-1.33180300	-0.14868300
C	1.82404500	0.97008800	-0.81929200
C	2.70372500	-0.08639000	-0.64828000
H	-3.29837000	-0.71041100	1.02248400

H	-2.41073200	0.81666600	0.92666800	
H	-1.77181100	-2.94299200	1.18463200	
H	0.65831900	-3.46191600	0.85289600	
H	-3.29708700	-0.88147300	-1.46244000	
H	-2.35567800	0.62136900	-1.57502800	
H	3.00119000	-2.13806400	-0.02391600	
H	-4.69723000	1.01519700	-1.87739200	
H	-4.17449200	1.77176200	-0.49948200	
H	-0.24229100	1.59719600	-0.76444500	
H	2.19142500	1.90792400	-1.22136200	
O	4.03351700	0.01325100	-0.97437600	
H	4.23216500	0.91003600	-1.27228900	
O	0.46018200	1.71111900	1.36071700	
O	0.78710700	3.05056700	1.20006500	
H	1.75440200	3.07444400	1.25186700	
H	-5.05272600	0.37435100	-0.39265700	
**Product-C9-RAF**				**$[C_{10}H_{13}N_2O]^+OOH$**
1 2				
N	-0.36919500	-2.62837200	-0.41700200	
N	4.29804600	0.78332900	0.82967500	
C	1.18295600	-1.01210100	-0.39842200	
C	-0.07300700	-0.43721600	-0.09772500	
C	2.50720700	-0.31487300	-0.47206400	

C	-1.02959000	-1.46070300	-0.11910100
C	0.95900300	-2.36751400	-0.58461300
C	3.01941600	0.01260200	0.92340000
C	-0.46743600	0.97676500	0.14824900
C	-2.38941800	-1.26309500	0.14620600
C	-1.91145800	1.11239500	0.52578800
C	-2.78089600	0.06694200	0.47776800
H	3.23389600	-0.95032300	-0.98453100
H	2.41042000	0.61342700	-1.04289200
H	1.65603500	-3.15459000	-0.82784100
H	-0.80055200	-3.53986300	-0.48839200
H	3.23088900	-0.88982900	1.49401700
H	2.31654500	0.63324300	1.47658000
H	-3.11700600	-2.06369400	0.12305600
H	4.66966200	1.00051300	1.75770400
H	4.15385100	1.66557900	0.33028900
H	0.18111700	1.46108600	0.88656700
H	-2.27056400	2.09409300	0.81689900
O	-4.11812600	0.20483900	0.77133900
H	-4.31684500	1.13354500	0.94637000
O	-0.18902200	1.66758000	-1.11478300
O	-0.28656200	3.06734400	-0.89207800
H	-1.20991100	3.26507400	-1.11074000
H	5.00953600	0.24972100	0.32145900

TS-C10-RAF	$[C_{10}H_{13}N_2O]^+ + HOO^{\cdot}$

1 2			
N	-0.10571200	1.99679700	-0.67308600
N	5.01243400	-0.87444200	0.14481200
C	1.60979600	0.81890200	0.16836400
C	0.45793300	-0.03545200	0.07194200
C	2.97244200	0.42960400	0.64946200
C	-0.58494600	0.73785700	-0.46066700
C	1.21739300	2.04647800	-0.29818400
C	3.66911600	-0.46314600	-0.36830400
C	0.22381200	-1.39470500	0.37553500
C	-1.90664900	0.24461400	-0.62885800
C	-1.02593200	-1.94104400	0.12932200
C	-2.05176900	-1.15613900	-0.40872000
H	3.57563200	1.32530000	0.81858400
H	2.89672900	-0.10822400	1.60033500
H	1.78159400	2.96154900	-0.39323300
H	-0.64026300	2.77469500	-1.03515300
H	3.83382800	0.05823300	-1.30945100
H	3.10845100	-1.37752800	-0.55553500
H	5.49658900	-1.47784800	-0.52446000
H	4.92379800	-1.38632600	1.02743100
H	1.01519900	-2.01139400	0.78683400

H	-1.22410800	-2.98785100	0.33211300	
O	-3.27994500	-1.66075200	-0.68229200	
H	-3.31823600	-2.59635900	-0.43973900	
H	-2.60945300	0.74364700	-1.28533500	
O	-2.78943100	0.92660200	0.93377000	
O	-4.16697200	0.81341700	0.73869900	
H	-4.38498900	-0.07523800	1.05653300	
H	5.60178100	-0.05508600	0.31822800	
**Product-C10-RAF**				**$[C_{10}H_{13}N_2O]^+OOH$**
1 2				
N	-0.15280200	-2.01419400	0.40236400	
N	5.02353500	0.83573500	0.10102200	
C	1.61661400	-0.81293900	-0.25196500	
C	0.46916200	0.04847500	-0.18015000	
C	3.01028600	-0.40617100	-0.61771900	
C	-0.60159900	-0.73915200	0.22653900	
C	1.18860400	-2.06601400	0.11158700	
C	3.65205600	0.39585200	0.50539000	
C	0.29334100	1.44490300	-0.40935000	
C	-1.98969700	-0.26491100	0.40749900	
C	-0.97895900	1.99436500	-0.19475600	
C	-2.03863500	1.22065400	0.21816200	
H	3.61550100	-1.29398100	-0.81920200	

H	2.99817300	0.20351100	-1.52746400
H	1.73475800	-2.99386100	0.18222200
H	-0.72362000	-2.80487100	0.66957500
H	3.76267800	-0.20069900	1.40906000
H	3.08503100	1.29674300	0.73482000
H	5.47832900	1.36698700	0.84774800
H	4.98443100	1.42953700	-0.73249600
H	1.11686100	2.06806800	-0.73408900
H	-1.14863300	3.05605900	-0.34509300
O	-3.29141500	1.71713200	0.43666400
H	-3.31570900	2.66276300	0.23443400
H	-2.43090100	-0.56313600	1.36572600
O	-2.80650700	-0.92243500	-0.61738300
O	-4.17019300	-0.90751500	-0.21740100
H	-4.44812800	0.00574300	-0.39645700
H	5.61398600	0.02851800	-0.11893900

**TS-C11-RAF**	**$[C_{10}H_{13}N_2O]^+ + HOO^{\cdot}$**

1 2

N	0.53500700	2.57571500	0.28918200
N	4.49291700	-1.79821400	-0.42867600
C	1.76783800	0.69789100	0.45321700
C	0.41500800	0.34361400	0.07518800
C	2.90690500	-0.24927700	0.65759300

C	-0.31757500	1.56564900	-0.01933900
C	1.78953600	2.05309400	0.57332300
C	3.37638900	-0.83297200	-0.66807500
C	-0.21732500	-0.85321000	-0.20936700
C	-1.67502300	1.61651100	-0.38159000
C	-1.61074400	-0.85124400	-0.49170900
C	-2.29132700	0.42016200	-0.63238700
H	3.73869200	0.27320900	1.13709300
H	2.59968400	-1.06811200	1.31705100
H	2.60181000	2.70868400	0.84808300
H	0.29333000	3.55676300	0.31477400
H	3.75929000	-0.05939500	-1.33112600
H	2.58453900	-1.38328000	-1.17350900
H	-2.21755700	2.55083600	-0.45530500
H	4.83006000	-2.20534300	-1.30479700
H	4.18629400	-2.56399300	0.17813600
H	0.31949000	-1.79484000	-0.19514900
H	-2.02509700	-1.68550400	-1.04849600
O	-3.60757300	0.40918900	-0.98603400
H	-3.97703000	-0.46190600	-0.76657400
O	-2.43172600	-1.45750000	1.09134700
O	-3.75318300	-1.79283600	0.75115600
H	-3.69458900	-2.70681500	0.43423200
H	5.28338200	-1.33494600	0.02915900

Product-C11-RAF	$[C_{10}H_{13}N_2O]^+OOH$

1 2

N	-0.49793500	2.59429900	-0.25500300
N	-4.46408500	-1.78497000	0.43649100
C	-1.75596300	0.73925400	-0.42605900
C	-0.39555700	0.35499900	-0.10437000
C	-2.90793100	-0.19189200	-0.63162600
C	0.34821000	1.55930200	-0.00387300
C	-1.76664400	2.10085600	-0.51005800
C	-3.34726900	-0.82109900	0.68341900
C	0.21754200	-0.86658100	0.11147400
C	1.72623400	1.58895300	0.35140100
C	1.68891800	-0.93150400	0.32288800
C	2.34917200	0.40434700	0.54378900
H	-3.74945300	0.35042900	-1.07068100
H	-2.62356400	-0.98946800	-1.32707400
H	-2.57809700	2.77331300	-0.74260100
H	-0.24273600	3.57202600	-0.25380800
H	-3.72002900	-0.07199800	1.37953300
H	-2.54263500	-1.38352900	1.15427600
H	2.25476800	2.52365200	0.49840200
H	-4.78540900	-2.21614200	1.30686800
H	-4.16334800	-2.53316000	-0.19475000

H	-0.32742700	-1.80226500	0.07981100
H	1.96286400	-1.61771900	1.13352100
O	3.64788300	0.38465000	0.97192100
H	4.00306600	-0.50990900	0.87089900
O	2.21292400	-1.55641200	-0.90155700
O	3.57869400	-1.91998700	-0.67609100
H	4.05326200	-1.33267300	-1.28380000
H	-5.26338800	-1.31368500	0.00288800
**TS-C12-RAF**		**$[C_{10}H_{13}N_2O]^+$ + $HOO^{\cdot}$**	
1 2			
N	0.33751900	2.43746700	-0.05421300
N	4.73455800	-1.53517300	-0.12019200
C	1.72163300	0.71918000	0.39633400
C	0.45673200	0.18860600	0.03458000
C	2.95249700	-0.05688100	0.74715800
C	-0.40432200	1.29105900	-0.24133400
C	1.60120900	2.09473100	0.32734200
C	3.55800900	-0.69291000	-0.49656400
C	-0.04086100	-1.13504800	-0.11340000
C	-1.71113000	1.13847800	-0.64675400
C	-1.33966500	-1.31761400	-0.47621200
C	-2.22832100	-0.18774100	-0.64910600
H	3.68777300	0.60238300	1.21484900

H	2.70276800	-0.84498800	1.46477600
H	2.34100500	2.85450800	0.53092600
H	-0.00798500	3.38166200	-0.15657400
H	3.91421600	0.05965700	-1.19761600
H	2.84860700	-1.34766600	-0.99997100
H	-2.35957000	1.97086600	-0.89041700
H	5.15813800	-1.97069400	-0.94360400
H	4.45577200	-2.28044900	0.52455300
H	0.61256300	-1.98461700	0.05142400
H	-1.76180200	-2.30926800	-0.59844000
O	-3.39924600	-0.36401200	-1.32068700
H	-3.66789000	-1.29202600	-1.27322400
O	-2.94882400	-0.13552700	1.18102200
O	-3.85161700	-1.19999000	1.25832500
H	-4.67861700	-0.83782100	0.90829100
H	5.45329300	-0.97253500	0.34430800

**Product-C12-RAF**	**$[C_{10}H_{13}N_2O]^+OOH$**

1 2

N	-0.40797200	2.46223700	0.06276600
N	-4.69845700	-1.61303000	0.21902000
C	-1.77448300	0.73256200	-0.39565300
C	-0.47811800	0.22355700	-0.17600700
C	-3.01140600	-0.05533500	-0.69754300

C	0.38096200	1.32719300	0.10890800
C	-1.69095900	2.10954200	-0.24058900
C	-3.50552400	-0.77434500	0.55036500
C	0.06927300	-1.10453200	-0.16666200
C	1.71500600	1.20295700	0.38419600
C	1.37948600	-1.29159400	0.07930600
C	2.33585800	-0.15319900	0.33062600
H	-3.79581800	0.60906200	-1.06845500
H	-2.80146000	-0.79515700	-1.47636100
H	-2.46527200	2.85609900	-0.33357100
H	-0.07941000	3.40702300	0.20511100
H	-3.81305400	-0.07079100	1.32178100
H	-2.74904200	-1.44510300	0.95441700
H	2.35659600	2.04179400	0.62411600
H	-5.05162000	-2.10167100	1.04552800
H	-4.46347100	-2.31534400	-0.48864300
H	-0.58233900	-1.95341800	-0.34681500
H	1.80964100	-2.28713000	0.10937600
O	3.08182000	-0.35738900	1.51065400
H	3.47488300	-1.24081200	1.47343000
O	3.27745800	-0.04535800	-0.77699200
O	4.04665400	-1.24289600	-0.85297700
H	3.59434500	-1.74931800	-1.54491400
H	-5.45699700	-1.03536300	-0.15465500

Table S12 Optimized structure forms of HOO in gas phase and water using □□□□□X/6-311++g(d,p)

<table>
<tr><th>Radical $HOO^{\bullet}$-Gas</th><th>$[HOO]^{\bullet}$</th></tr>
<tr><td colspan="2">0 2
O 0.05493500 -0.59902000 0.00000000
H -0.87895400 -0.87292300 0.00000000
O 0.05493500 0.70813500 0.00000000</td></tr>
<tr><th>HOOH-Gas</th><th>HOOH</th></tr>
<tr><td colspan="2">0 1
O 0.00000000 0.71202700 -0.05308300
H 0.81565400 0.90168500 0.42466700
O 0.00000000 -0.71202700 -0.05308300
H -0.81565400 -0.90168500 0.42466700</td></tr>
<tr><th>Radical $HOO^{\bullet}$-Water</th><th>$[HOO]^{\bullet}$</th></tr>
<tr><td colspan="2">0 2
O 0.05492900 -0.59527600 0.00000000
H -0.87885700 -0.88428400 0.00000000
O 0.05492900 0.70581200 0.00000000</td></tr>
<tr><th>HOOH-Water</th><th>HOOH</th></tr>
<tr><td colspan="2">0 1
O 0.00000000 0.71025600 -0.06975600</td></tr>
</table>

H	0.70427600	0.92763500	0.55804600
O	0.00000000	-0.71025600	-0.06975600
H	-0.70427600	-0.92763500	0.55804600
